# Magneto-Optical Spectroscopy Across Three Oxidation States of a Europium Terpyridine Complex

**DOI:** 10.1021/acs.inorgchem.6c02413

**Published:** 2026-08-18

**Authors:** Che Wu, Elizabeth L. Fosnocht, Nicholas A. Moriglioni, Wesley J. Transue

**Affiliations:** Department of Chemistry, 6614 University of Pittsburgh, Pittsburgh, Pennsylvania 15213, United States

## Abstract

We report the magneto-optical characterization of a homoleptic europium terpyridine complex in +3, +2, and formally 0 oxidation states. Magnetic circular dichroism (MCD) analysis of the f–f transitions of the Eu­(III) complex revealed an effective *D*_3h_ local symmetry in solution, higher than suggested by the solid-state structure, through the splitting pattern of its ^7^F_0_→^5^D_2_ transition. The Eu­(II) complex, by contrast, exhibited broad MCD features spanning the visible region arising from 4f^7^[^8^S_7/2_]→4f^6^[^7^F_*J*_]×ψ^1^ transitions (ψ = 5d or Lπ*, *J* = 0–6). We show theoretically and experimentally that when individual components of this *J* progression are unresolved, MCD and magnetic linear dichroism (MLD) spectra display characteristic “pseudo-*A*_1_” and “pseudo-*A*_2_” lineshapes, respectively. Variable-temperature variable-field measurements of both techniques reveal a near-zero Eu­(II) zero-field splitting by monitoring MCD *C*_0_ and MLD *G*_0_ signal intensities. The formally Eu(0) complex was serendipitously isolated and its properties are consistent with a redox noninnocent [Eu^II^(mtpy)­(mtpy^•–^)_2_] formulation. Together, these results establish MCD and MLD spectroscopy as information-rich tools for probing f-block electronic structure across multiple oxidation states and transition types.

## Graphical abstract

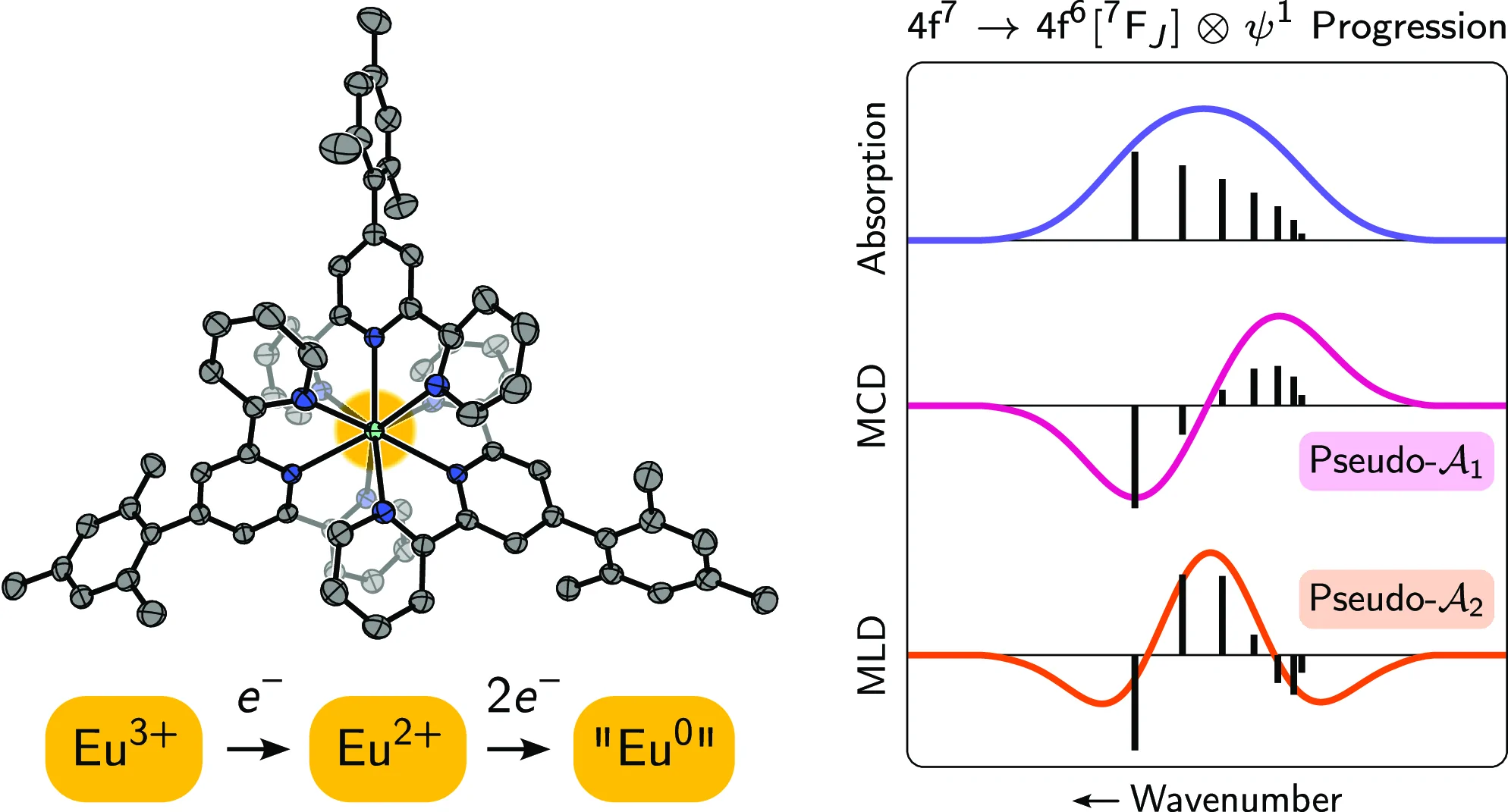

## 1 Introduction

The chemistry of europium is dominated by the +3 (4f^6^) and +2 (4f^7^) oxidation states. The single-electron difference between these states produces a large change in orbital angular momentum (^7^F vs ^8^S) and causes dramatic differences in reactivity, photophysical properties, and magnetism. These sharp differences in optical and magnetic properties make the Eu^II/III^ couple an interesting platform to study the impact of electronic structure on light–matter interactions with implications on excited state quenching, photoexcited spin polarization, and the design of donor–acceptor antenna compounds.

Magneto-optical spectroscopies like magnetic circular dichroism (MCD) and magnetic linear dichroism (MLD) offer precise insight into the interplay of electronic structure, symmetry, and magnetism.^1^ These two techniques measure the differential absorption of polarized light in the presence of an externally applied magnetic field. The complementary selection rules of MCD and MLD mean that joint acquisition of both spectra in the UV–vis–NIR window can greatly facilitate assignment of electronic transitions.^2,3^ MCD has been extensively used in the f-block to characterize electronic structure,^4,5^ but we are only aware of a handful of MLD studies on f-block ions,^6,7^ and none but our recent report have focused on molecular complexes.^2^

We have selected a homoleptic europium terpyridine system (Scheme 1) to explore these magneto-optical behaviors in a *D*_3_-symmetric coordination environment. A unifying feature of the electronic structures of this system across oxidation states is the central role of ^7^F_*J*_ (*J* = 0–6) states in the spectra: Eu^III^ displays sharp 4f–4f transitions involving ^7^F_*J*_ levels, while Eu^II^ displays broader 4f–5d and metal-to-ligand charge transfer (MLCT) transitions with underlying 4f^6^[^7^F_*J*_]⊗ψ^1^ fine structure (*J* = 0–6, ψ = 5d or ligand π*). We develop theoretical expressions for the MCD and MLD intensity patterns of the Eu^II^ ion both in presence and absence of magnetic saturation. This framework describes most of our experimental observations, with deviations likely arising from strong spin–orbit coupling (SOC) between energetically proximal 4f^6^⊗5d^1^ states. We additionally report the serendipitous isolation and preliminary characterization of the formally europium(0) species, in which redox noninnocent terpyridine ligands are implicated by crystallographic and spectroscopic evidence. The noninnocent behavior is well-known for oligopyridines and phenanthroline in d-block complexes but less explored for lanthanide complexes, especially in the case of terpyridine.^8–14^

**Scheme 1:**
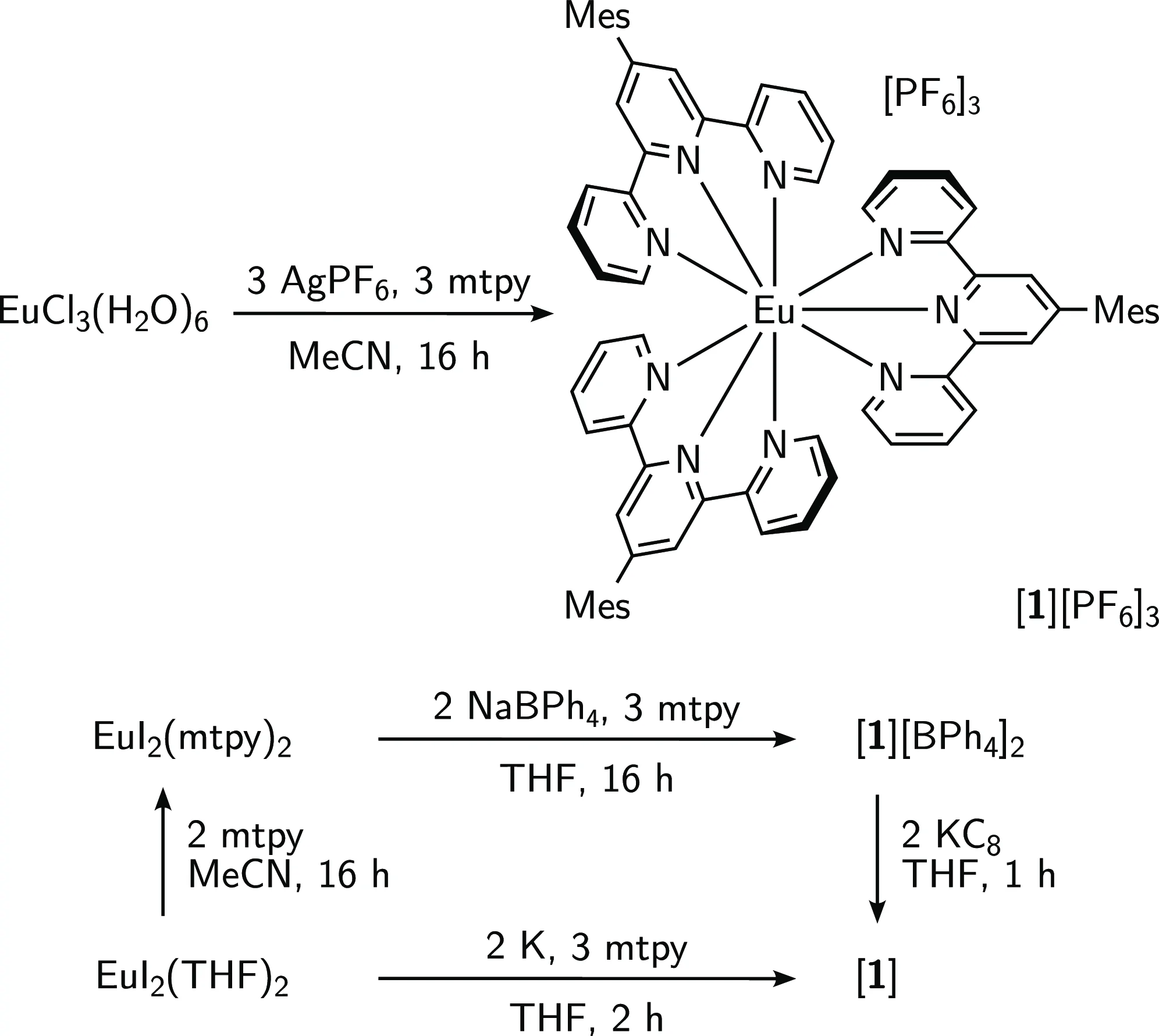
The tris­(mesitylterpyridine)europium System [**1**]^*n*+^ can be Accessed in Three Oxidation States (mtpy = 4′-mesityl-2,2′:6′,2^″^-Terpyridine)

## 2 Theory

### 2.1 Basis of (Magneto-)­Optical Spectroscopies

The absorption, MCD, and MLD intensities of a transition between an initial state *A* and a final state *J* all depend on the transition dipole moments between the states. We begin by summarizing their intensity expressions, orientational averaging, and lineshapes.

#### 2.1.1 Absorption

The absorptivity (ϵ) of a molecule with light traveling along the molecular *z* axis can be expressed

1 $$\frac{\epsilon }{E}=\frac{\gamma }{2}\sum_{\begin{array}{l} a\in A\\ j\in J \end{array}}N_{a}\left({\left|\langle a|m_{x}|j\rangle \right|}^{2}+{\left|\langle a|m_{y}|j\rangle \right|}^{2}\right)f\left(E\right)$$

Here, the magnetic sublevels of states *A* and *J* are indexed using lowercase *a* and *j*, respectively, and we have denoted the fractional Boltzmann population of a sublevel *a* as *N*_*a*_, the energy of incident light as *E*, the line shape function for the transition as *f*(*E*), the transition dipole operator as *m⃗*, and we have used γ as a proportionality constant. We assume negligible population of the *J* excited state and that vibronic complications can be ignored.

Molecules dissolved in solution or frozen glasses generally have an isotropic distribution of molecular orientations, so eq 1 must be averaged over all possible directions of light propagation in the molecular frame. Doing so yields

2 $$\begin{array}{ll} \frac{\left\langle \epsilon \right\rangle }{E} & =\int \frac{\gamma }{2}\sum_{\begin{array}{l} a\in A\\ j\in J \end{array}}N_{a}\langle a|\mathrm{m⃗}|j\rangle \left(1-{ẑ}_{lab}{ẑ}_{lab}^{T}\right)\langle j|\mathrm{m⃗}|a\rangle f\left(E\right)\frac{d{ẑ}_{lab}}{4\pi }\\ & =\frac{\gamma }{3}\sum_{\begin{array}{l} a\in A\\ j\in J \end{array}}N_{a}{\left|\langle a|\mathrm{m⃗}|j\rangle \right|}^{2}f\left(E\right)=\gamma D_{0}f\left(E\right), \end{array}$$

where the chevrons around ⟨ϵ⟩ indicate an orientationally averaged quantity, ${ẑ}_{lab}$ is the direction of light propagation in the laboratory frame, and we have introduced the dipole strength $D_{0}$ for the *A* → *J* transition.

#### 2.1.2 MCD

MCD spectroscopy measures the differential absorption of left- and right-circularly polarized light (Δϵ_MCD_ = ϵ_LCP_–ϵ_RCP_) upon application of an external magnetic field parallel to the direction of propagation of light. Unlike natural circular dichroism, MCD can be observed for all matter and it is, in fact, insensitive to the handedness of a chiral system.^15^ Its intensity expression is

3 $$\frac{\Delta \epsilon_{MCD}}{E}=\gamma \sum_{\begin{array}{l} a\in A\\ j\in J \end{array}}N_{a}\left({\left|\langle a|m_{-}|j\rangle \right|}^{2}-{\left|\langle a|m_{+}|j\rangle \right|}^{2}\right)f\left(E\right)$$

where $m_{\pm }=\mp \left(m_{x}\pm im_{y}\right)/\sqrt{2}$ such that *m*_–_ indicates left circular polarization (LCP) and *m*_+_ indicates right circular polarization (RCP). When comparing MCD spectra between reports, care must be taken to ensure use of the same convention for definition of LCP/RCP light: we use the convention in Piepho and Schatz,^15^ but the opposite convention can sometimes be encountered in the literature.^16^

Following the standard treatment laid out by Piepho and Schatz^15^ and others, orientationally averaged MCD intensity can be expressed

4 $$\frac{\left\langle \Delta \epsilon_{MCD}\right\rangle }{E}=\int i\gamma \sum_{\begin{array}{l} a\in A\\ j\in J \end{array}}N_{a}{ẑ}_{lab}\cdot \langle a|\mathrm{m⃗}|j\rangle \times \langle j|\mathrm{m⃗}|a\rangle f\left(E\right)\frac{d{ẑ}_{lab}}{4\pi }$$

where the directions of light propagation and the external magnetic field are both along the laboratory ${ẑ}_{lab}$ axis. When the system is far from magnetic saturation (μ_*B*_ *B*/*k*_*B*_ *T* ≪ 1), Taylor expansion in the applied magnetic field *B* provides the standard linearized MCD equation^17^

$$\frac{\left\langle \Delta \epsilon_{MCD}\right\rangle }{E}=\gamma \mu_{B}B\left[-A_{1}\frac{\partial f}{\partial E}+\left(B_{0}+\frac{C_{0}}{k_{B}T}\right)f\right]$$ 5

where μ_*B*_ is the Bohr magneton, *k*_*B*_ is the Boltzmann constant, and *T* is temperature. The $A_{1}$ , $B_{0}$ , and $C_{0}$ constants are the so-called Faraday A-, B-, and C-term parameters, and they describe the line shape (Figure 1) and sensitivity to field and temperature. The $A_{1}$ term produces a bisignate (derivative-shaped) signal that arises from field-induced splitting of the excited state sublevels *j* ∈ *J*. The $B_{0}$ term produces a temperature-independent monosignate (absorption-shaped) signal that arises from Zeeman-induced mixing of states. The $C_{0}$ term produces a temperature-dependent (*T* ^–1^) monosignate signal that arises from unequal Boltzmann population of Zeeman-split ground state sublevels *a* ∈ *A*; it is the dominant contributor for paramagnetic systems at cryogenic temperatures.

**Figure 1:**
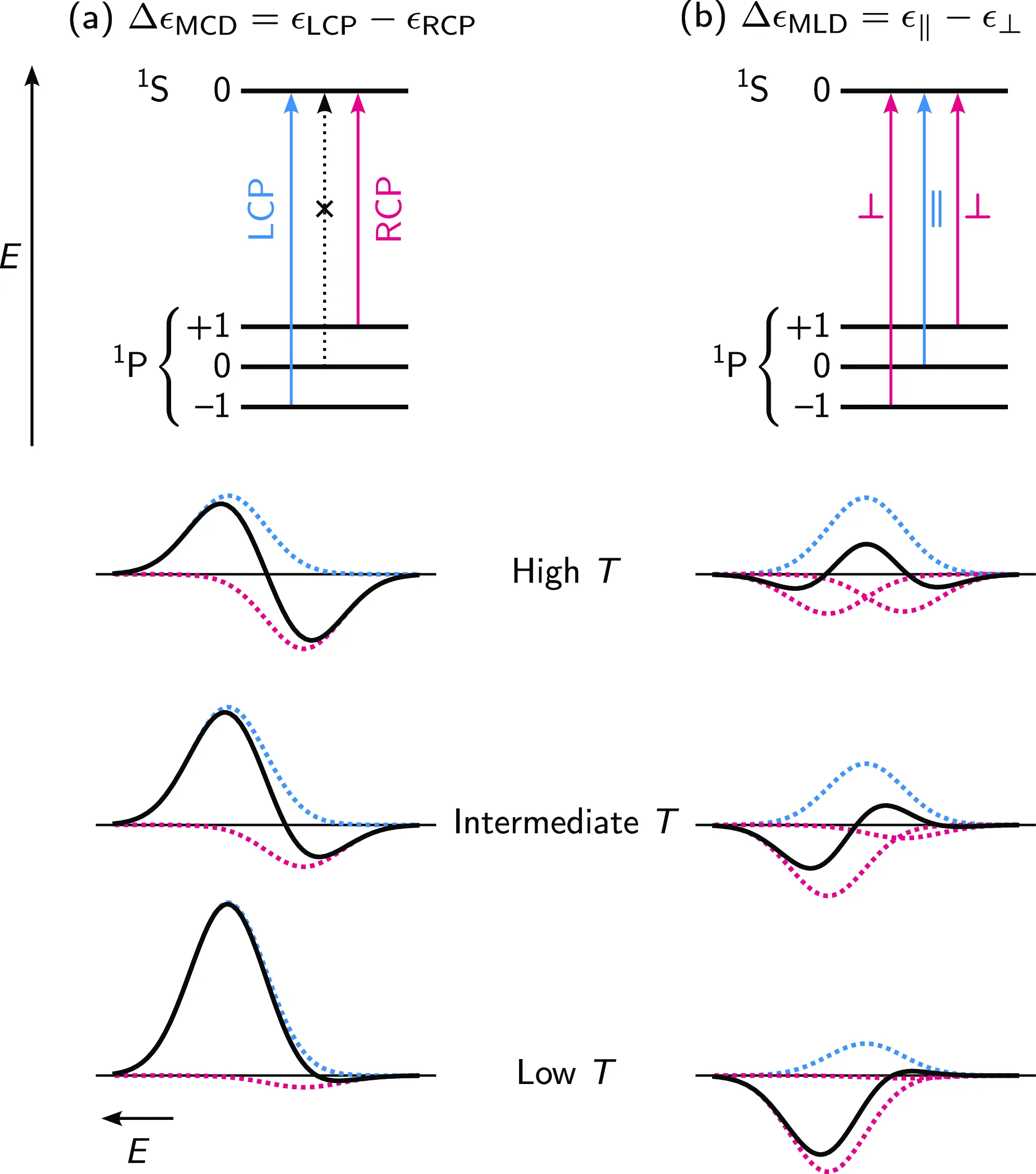
The lineshapes seen in (a) MCD and (b) MLD spectra vary with temperature and field according to the Boltzmann populations of the magnetic sublevels. Features are generally monosignate (MCD $C_{0}$ and MLD $G_{0}$ ) at very low temperatures, and they can develop more complicated bi- and trisignate profiles as the temperature rises.

**Figure 2:**
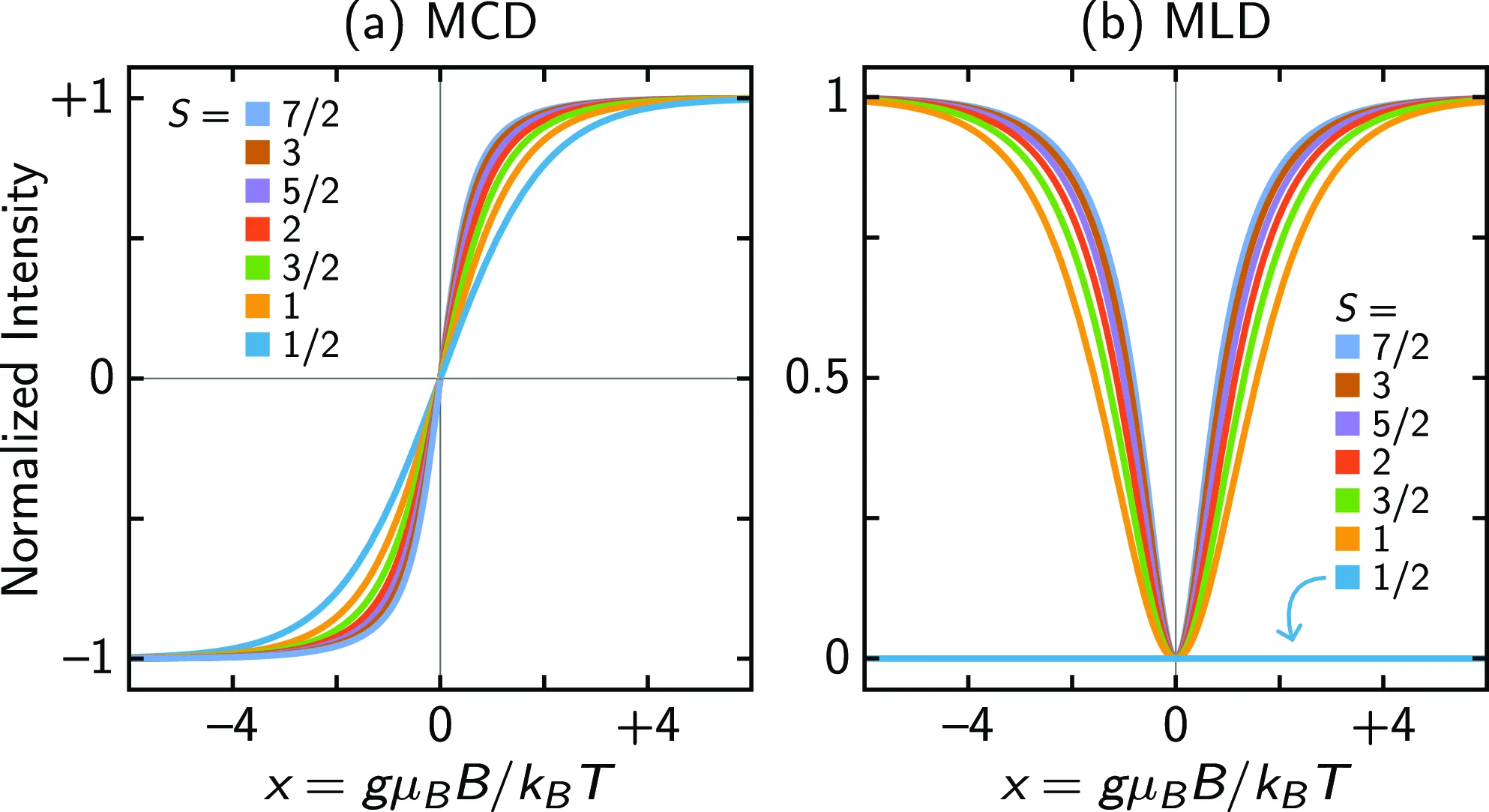
Low temperatures and high fields cause the system to experience magnetic saturation effects, which are seen as deviations from (a) a linear $C_{0}$ MCD response and (b) a quadratic $G_{0}$ MLD response. The saturation curves plotted here, eqs 10 and 11, assume no zero-field splitting and an isotropic *g* value.

#### 2.1.3 MLD

MLD spectroscopy measures the differential absorption of light polarized parallel and perpendicular to the direction of an external magnetic field, Δϵ_MLD_ = ϵ_∥_–ϵ_⊥_, where the magnetic field must be applied 90° to the direction of propagation of light. Its intensity can be expressed^1^

6 $$\frac{\Delta \epsilon_{MLD}}{E}=\gamma \sum_{\begin{array}{l} a\in A\\ j\in J \end{array}}N_{a}\left({\left|\langle a|m_{∥}|j\rangle \right|}^{2}-{\left|\langle a|m_{⊥}|j\rangle \right|}^{2}\right)f\left(E\right)$$

We seek an orientationally averaged expression, so we begin by assuming the field is applied along +*z* (∥) and light propagates in the *xy* plane (⊥). We next average over all directions of light propagation within the *xy* plane by setting *m*_∥_ = *m*_*z*_ and *m*_⊥_ = (cos ψ)*m*_*x*_ + (sin ψ)*m*_*y*_ then integrating over ψ to obtain

7 $$\frac{\langle \Delta {\epsilon MLD\rangle }_{xy}}{E}=\gamma \sum_{\begin{array}{l} a\in A\\ j\in J \end{array}}N_{a}\left({\left|\langle a|m_{z}|j\rangle \right|}^{2}-\frac{1}{2}{\left|\langle a|m_{x}|j\rangle \right|}^{2}-\frac{1}{2}{\left|\langle a|m_{y}|j\rangle \right|}^{2}\right)f\left(E\right)$$

We finally rewrite in the molecular frame assuming the applied field is along the laboratory ${ẑ}_{lab}$ direction, yielding

8 $$\frac{\left\langle \Delta \epsilon_{MLD}\right\rangle }{E}=\int \gamma \sum_{\begin{array}{l} a\in A\\ j\in J \end{array}}N_{a}\langle a|\mathrm{m⃗}|j\rangle \cdot \left(\frac{3{ẑ}_{lab}{ẑ}_{lab}^{T}-1}{2}\right)\cdot \langle j|\mathrm{m⃗}|a\rangle f\left(E\right)\frac{d{ẑ}_{lab}}{4\pi }$$

We stress that this expression is only valid when averaging over an isotropic orientational distribution of molecules. In the absence of magnetic saturation (μ_*B*_ *B*/*k*_*B*_ *T* ≪ 1), Taylor expansion gives

$$\frac{\left\langle \Delta \epsilon_{MLD}\right\rangle }{E}=\gamma \mu_{B}^{2}B^{2}\left[A_{2}\left(\frac{1}{2}\frac{\partial^{2}f}{\partial E^{2}}\right)-\left(B_{1}+\frac{C_{1}}{k_{B}T}\right)\frac{\partial f}{\partial E}+\left(E_{0}+\frac{F_{0}}{k_{B}T}+\frac{G_{0}}{{\left(k_{B}T\right)}^{2}}\right)f\right]$$ 9

Six Faraday terms are required to parametrize the MLD response, yielding trisignate (second-derivative-shaped, $A_{2}$ ), bisignate ( $B_{1}$ , $C_{1}$ ), and monosignate ( $E_{0}$ , $F_{0}$ , $G_{0}$ ) lineshapes (Figure 1). Note the *B* ^2^ quadratic response of MLD reflects its second-order origin and contrasts with the *B* linear response of first-order MCD. As with MCD Faraday terms, intensity mechanisms can be ascribed to each of the MLD Faraday terms;^18^ however, the only one of importance to us in this study is the $G_{0}$ term, which arises from the unequal Boltzmann population of three-or-more ground state magnetic sublevels *a* ∈ *A* (vide infra).

### 2.2 Saturation Magnetization Behavior

The dominant intensity mechanisms for paramagnetic metal complexes, $C_{0}$ in MCD and $G_{0}$ in MLD, encode information about the ground state spin Hamiltonian, such as spin multiplicity, *g* values, and zero-field splitting (ZFS). The sensitivities to these parameters are most obvious when sufficiently cold temperatures and strong fields are used such that the sample is no longer in the weak-field limit. Under such conditions, the MCD and MLD signals deviate from their respective linear (*B*/*T*) and quadratic (*B* ^2^/*T* ^2^) responses as the system begins to experience magnetic saturation (Figure 2). Variable-temperature/variable-field (VTVH) measurements of the saturation curves can provide detailed magnetic insight into the molecular magnetism.

The MCD $C_{0}$ saturation response has been reported many times. Systems with an approximately isotropic magnetic response follow a simple Brillouin curve^19^

10 $$\frac{\left\langle \Delta \epsilon_{MCD}\right\rangle }{E}=\frac{3\gamma C_{0}}{g\left(S+1\right)}\left\{\frac{2S+1}{2S}\coth\left(\frac{2S+1}{2}x\right)-\frac{1}{2S}\coth\left(\frac{1}{2}x\right)\right\}f\left(E\right)$$

where *x* = *g*μ*_B_B*/*k*_*B*_ *T*. This equation can be expected to describe magnetization well for systems with isotropic *g* values that either lack ZFS (i.e., *S* = 1/2) or have particularly small ZFS values, as is often the case for f^7^ (Eu^II^, Gd^III^) and many high-spin d^5^ (Mn^II^, Fe^III^) complexes. Systems that do not meet these criteria exhibit more complicated saturation responses that are best modeled using a spin Hamiltonian approach.^20–23^

When present, MLD $G_{0}$ intensity is expected to be the dominant response for paramagnetic ions at low temperature; however, it has an additional constraint in its appearance beyond those of MCD $C_{0}$ intensity. Systems with isolated doublets or isolated effective doublets as their ground states cannot produce nonzero $G_{0}$ intensity.^24,25^ This follows from the $G_{0}$ intensity expression, which vanishes upon orientational averaging unless there are at least three distinct ground state sublevels *a* ≠ *a*′ ≠ *a*″. As such, $G_{0}$ intensity will be seen only for *S* ≥ 1 (or *J* ≥ 1) systems and it will be largest for those with little-to-no ZFS (|*D*| ≲ 5 cm^–1^). The $G_{0}$ intensity can be expected to gradually decrease in intensity as the size of the ZFS increases because large ZFS will isolate an effective doublet or singlet as the ground magnetic state. In the limit of no ZFS, the MLD $G_{0}$ saturation response can be analytically predicted to be

11 $$\frac{\left\langle \Delta \epsilon_{MLD}\right\rangle }{E}=\frac{30\gamma G_{0}}{g^{2}\left(S+1\right)\left(2S+3\right)}\frac{\sum_{M=-S}^{S}\frac{3M^{2}-S\left(S+1\right)}{S\left(2S-1\right)}e^{Mx}}{\sum_{M=-S}^{S}e^{Mx}}$$

where we still use *x* = *g*μ*_B_B*/*k*_*B*_ *T* and we require *S* ≥ 1 (see Supporting Information for derivation).^26^ Nonzero ZFS can be expected to lead to nesting in the VTVH MLD magnetization curves analogously to nesting in VTVH MCD curves. This more complicated behavior would require a spin Hamiltonian approach.

### 2.3 Non-f–f Transition Dipole Moments for 4f^7^ Ions

There are two types of likely transitions for a Eu­(II) coordination complex: 4f–5d transitions (f^7^ → f^6^ ⊗ d^1^) and metal-to-ligand charge transfer transitions $\left(f^{7}\to f^{6}⊗{\left(L\pi^{*}\right)}^{1}\right)$ . Both types of excitations leave six electrons within the f orbitals, and the strong 4f SOC of Eu^2+^ (ζ_4*f*_ = 1181 cm^–1^ in the 4f^6^5d^1^ manifold of its atomic ion)^27^ will cause intrastate splitting into a series of ^7^F_*J*_ states (*J* = 0–6). This fine structure can be experimentally observed in the absorption and/or luminescence spectra of systems with sufficiently narrow line widths, giving a ‘staircase’ pattern of features.^28–31^

The theoretical pattern of absorption $D_{0}$ , MCD $C_{0}$ , and MLD $G_{0}$ intensities within each 4f^6^[^7^F_*J*_]⊗ψ^1^ (ψ = 5d or Lπ*) progression for *J* = 0–6 can be predicted from the transition dipole moment associated with the single electron undergoing the 4f → ψ excitation. This can be accomplished using coefficients of fractional parentage (CFP), which allow any 4f^*N*^ wave function to be expressed in a |4f^*N*–1^⟩|4f^1^⟩ basis, effectively coupling the 4f^*N*–1^ manifold to the newly introduced electron. The transition dipole matrix element between the 4f^7^[^8^S] ground state and a 4f^6^[^7^F_*J*_]⊗ψ^1^ excited state is^32,33^

⟨f6[F7]J′M′,ψms|m⃗|f7[S8]M⟩=∑M̅SM̅L⟨f6⁢⁡F7|}f7⁡S8⟩⟨3M̅S,12ms|72M⟩⟨3M̅L,3−M̅L|00⟩⟨3M̅S,3M̅L|J′M′⟩⟨ψ|m⃗|f−M̅L⟩, 12

where ⟨f^6 7^F|}­f^7 8^S⟩ is a CFP (and happens to equal 1 here)^34^ and the $\left\langle J_{1}M_{1},J_{2}M_{2}|J_{3}M_{3}\right\rangle$ symbols are vector coupling (Clebsch–Gordan) coefficients. The single-electron reduced matrix elements ⟨ψ|m⃗|f⟩ are common to all *J* and we assume the f → ψ transition is allowed.

Combining eq 12 with eqs 2, 4, and 8 predicts the relative intensities for absorption $D_{0}$ , MCD $C_{0}$ , and MLD $G_{0}$ summarized in Table 1, where $D_{0}$ for the *J* = 0 transition has been normalized to 1. These values make no assumption of the identity of ψ, meaning they can be used for both 4f^6^[^7^F_*J*_]⊗5d^1^ and 4f6[FJ7]⊗(Lπ*)1 excited states. It is important to note that eq 12 does not take into account SOC within the 5d manifold. We anticipate this may often be reasonable because the Eu^2+^ 5d SOC constant (ζ_5*d*_ = 898 cm^–1^ in the 4f^6^5d^1^ manifold)^27^ is generally a weaker perturbation than crystal field interactions (e.g., Δ_Oct_ = 15 000–20 000 cm^–1^).^29^ The larger sizes of 5d orbitals also enable greater metal–ligand covalency and thus reduction in effective SOC through relativistic nephelauxesis. When molecular symmetry or accidental degeneracies cause two or more 4f^6^[^7^F_*J*_]⊗5d^1^ levels to approach in energy, SOC likely cannot be ignored in this way and deviations from Table 1 should be expected.

**Table 1:** Absorption, MCD, and MLD Intensities for f^7^ → f^6^ ⊗ ψ^1^ Transitions^a^

	f^6^[^7^F_*J*_]⊗ψ^1^		
^7^F_*J*_	$D_{0}$	$C_{0}$	$G_{0}$
*J* = 0	1	36/8	–36/16
*J* = 1	3	99/8	–81/16
*J* = 2	5	135/8	–57/16
*J* = 3	7	126/8	42/16
*J* = 4	9	54/8	162/16
*J* = 5	11	–99/8	165/16
*J* = 6	13	–351/8	–195/16

a Values are tabulated relative to $D_{0}$ for the *J* = 0 level. Fractions have been left unsimplified for ease of comparison of their magnitudes. These values assume that SOC within the ψ^1^ manifold can be ignored.

The “staircase” appearance previously reported in absorption and luminescence spectra is directly explained by Table 1. There are clearly also characteristic alternations in the signs and magnitudes of MCD and MLD features associated with these staircases. The MCD $C_{0}$ pattern changes sign from positive to negative once between J = 4–5; whereas, the MLD $G_{0}$ pattern changes sign twice from negative to positive between J = 2–3 and then from positive to negative between J = 5–6.

## 3 Results and Discussion

### 3.1 Synthesis and Crystallographic Characterization

This series of europium terpyridine complexes was synthesized as outlined in Scheme 1. The Eu­(III) tricationic complex was prepared by treating an acetonitrile solution of EuCl_3_·6H_2_O and mtpy (3 equiv) with excess AgPF_6_ (3.5 equiv), providing [**1**]­ ${\left[{PF}_{6}\right]}_{3}\cdot$ MeCN as a colorless and air-stable solid in good yield (71%) after recrystallization via layered diffusion of ether into a near-saturated acetonitrile solution.

A similar one-pot approach starting from Eu­(THF)_2_I_2_ did not yield the analogous Eu­(II) complex cleanly; instead, we first prepared an intermediate compound Eu­(mtpy)_2_I_2_·2MeCN from Eu­(THF)_2_I_2_ and mtpy (2 equiv). Subsequent treatment of Eu­(mtpy)_2_I_2_·2MeCN with mtpy (1 equiv) and excess NaBPh_4_ (2.2 equiv) in THF yielded crystals of [**1**]­ ${\left[{BPh}_{4}\right]}_{2}\cdot$ 2THF·Et_2_O in good yield (54% over two steps) after layered diffusion of ether into a THF solution. The use of NaBPh_4_ rather than a silver salt for halide abstraction was important due to undesired redox side reactions. The ease of oxidation of [**1**]^2+^ species was underscored by both its air sensitivity and by cyclic voltammetry, which showed a reversible Eu­(II/III) event at *E*_1/2_ = –0.784 V vs Fc/Fc^+^ (SI Figure S18).

Unexpectedly, a single crystal of the neutral, formally Eu(0) complex [**1**]^0^·2PhMe was obtained while exploring unrelated Eu­(II) chemistry. With this serendipitous crystal structure in hand, we pursued a purposeful synthetic route and found two convenient approaches. In the first, [**1**] ${\left[{BPh}_{4}\right]}_{2}$ was reduced in THF using excess KC_8_; an intense blue/green color developed rapidly, and full conversion was achieved after 1 h. The neutral complex was isolated as dark purple solids in 47% yield after recrystallization from pentane diffusion into a near-saturated toluene solution at –30 °C. In the second route, the neutral complex could be accessed directly by treating Eu­(THF)_2_I_2_ with mtpy (3 equiv) and potassium (2 equiv), giving the product in 24% yield. Crystals and solutions of [**1**]^0^·2PhMe were found to decompose rapidly in the presence of traces of oxygen or moisture. So far, attempts to access the formally Eu­(I) species through comproportionation of the neutral and dicationic species have not been successful.

X-ray crystallographic analysis was performed on all new compounds. The average Eu–N distances increased from the [**1**]^3+^ cation (2.562(2) Å) to the [**1**]^2+^ cation (2.698(3) Å), consistent with a metal-centered reduction lowering the positive charge at the europium ion. This trend of increasing Eu–N distance would be expected to continue in neutral [**1**]^0^ if its reduction were also metal-centered; instead, [**1**]^0^ showed a contracted distance of 2.555(2) Å. The break in the trend is not consistent with further metal-centered reduction but rather with ligand-centered reduction, the additional two electrons residing in the mtpy π systems. The resulting anionic ligands should bind the Eu­(II) center more strongly than the neutral mtpy ligands in [**1**]^2+^, decreasing the Eu–N distances. Radical character of the ligands was indicated by the decrease in the average length of the C(2)–C(2′) and C(6′)–C­(2^″^) bonds (denoted by C_py_–C_py_) and the flattened average N–C–C–N torsion angle.^10,35,36^ It was interesting to note that the heteroleptic Eu­(mtpy)_2_I_2_·2MeCN exhibited slightly elongated Eu–N distances of 2.717(2) Å, which may be attributable to the structural trans influence of the anionic iodide ligands across from the terpyridine ligands in the coordination sphere.^37^ All structures of [**1**]^*n*+^ species adopted distorted tricapped trigonal prismatic geometries (Figure 3). We have quantified the deviations from the ideal geometry using Continuous Shape Measure (CShM) as implemented in SHAPE 2.1 ^38,39^ (Figure 3b–d). As the formal oxidation state of europium decreased, the geometry of [**1**]^*n*+^ further deviated from the ideal tricapped trigonal prismatic geometry as indicated by the increasing CShM values.

**Figure 3:**
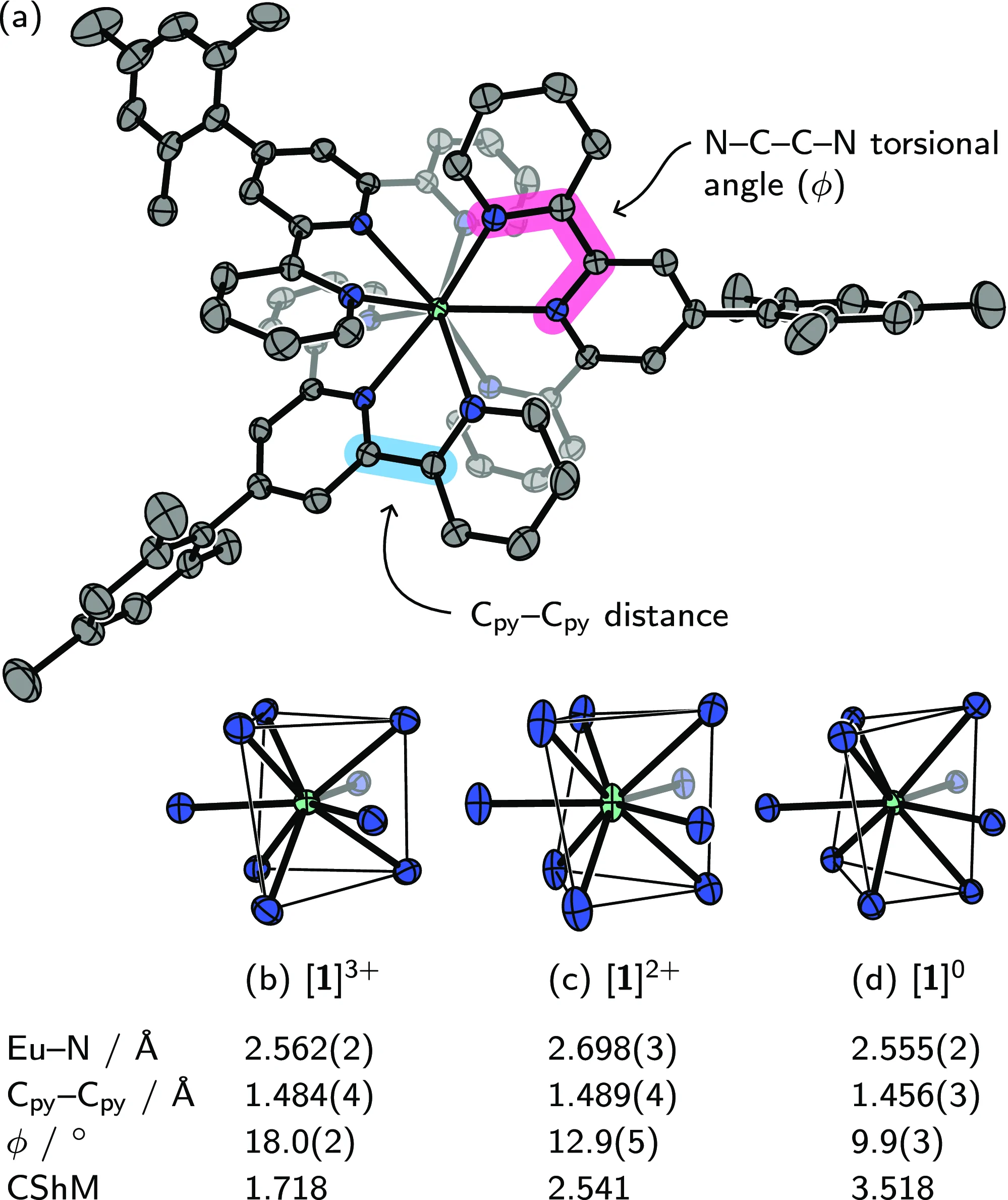
X-ray crystallography revealed all three [**1**]^*n*+^ systems to have approximate *D*_3_ point group symmetry, but deviations increased as the charge lowered from 3+ to 2+ to 0. (a) The structure of neutral [**1**]^0^·2PhMe is shown with 50% probability ellipsoids; hydrogens and solvents of crystallization are omitted for clarity. The coordination environment of the central Eu ion is roughly tricapped trigonal prismatic, and deviations are characterized by increases in the CShM value: (b) 1.718 for [**1**]­ ${\left[{PF}_{6}\right]}_{3}\cdot$ MeCN, (c) 2.541 for [**1**]­ ${\left[{BPh}_{4}\right]}_{2}\cdot$ 2THF·Et_2_O, and (d) 3.518 for [**1**]^0^·2PhMe.

### 3.2 Eu­(III): MCD, Luminescence, and Symmetry Assignment

While X-ray diffraction showed a geometry that was roughly *D*_3_ in symmetry, this does not necessarily mean such a geometry is retained in solution, where molecules tumble and flex due to time-varying interactions with solvent, counterions, and other solutes. Spectroscopic analysis performed directly on solutions can provide symmetry-based insight into the dynamic molecular structure, and different techniques capture that structure on different time scales. Our ^1^H NMR spectrum showed eight resonances, which implied a *D*_3_ molecular symmetry averaged over the NMR time scale (≥ms).^42^ The Franck–Condon principle suggests electronic transitions are effectively instantaneous in relation to nuclear motion, so absorption and MCD spectroscopies are ideal for providing direct snapshot of molecular symmetry rather than a time-averaged one. The narrow linewidths of f–f transitions further mean that MCD spectroscopy of lanthanide systems can achieve precision in symmetry determination through optical excitations that is seldom achieved elsewhere in the periodic table.

Axially symmetric Eu^III^ has been particularly well studied by MCD.^4,5,43–46^ Its nondegenerate ^7^F_0_ ground state causes the $A_{1}$ mechanism to dominate the MCD spectrum rather than the more common $C_{0}$ mechanism. The selection rules for $A_{1}$ MCD signals from the ^7^F_0_ ground state have been well developed:^43^ transitions will only exhibit $A_{1}$ intensity if (1) the triple product Γ_*i*_ ⊗Γ_*x*,*y*,*z*_ ⊗Γ_*f*_ contains the totally symmetric representation and (2) Γ_*f*_ is at least doubly degenerate; here, Γ_*i*_ and Γ_*f*_ are the irreps of the initial and final levels, respectively, and Γ_*x*,*y*,*z*_ is the (often reducible) representation for *x*, *y*, and *z*. When these conditions are met, the sign of the $A_{1}$ signal can be predicted within axial point groups using Judd–Ofelt theory as outlined by Görller-Walrand and Fluyt-Adriaens.^4^

The ^7^F_0_→^5^D_1_ and ^7^F_0_→^5^D_2_ transitions provide the most direct insight into molecular symmetry in the MCD spectrum. A *D*_3_-symmetric ion like [**1**]^3+^ is expected to show one positively signed $A_{1}$ -term signal in the ^7^F_0_→^5^D_1_ region, along with two oppositely signed $A_{1}$ -term signals in the ^7^F_0_→^5^D_2_ region. While Figure 4b does show a single positively signed $A_{1}$ signal at 19 115 cm^–1^ for the ^7^F_0_→^5^D_1_ transition, it unexpectedly shows only a single negative $A_{1}$ signal at 21 577 cm^–1^ for the ^7^F_0_→^5^D_2_ transition. The absence of a second ^5^D_2_ signal indicates that the metal center experiences a higher effective symmetry than that of the molecular geometry. Specifically, the spectrum indicates an effective *D*_3h_ symmetry, wherein only the A_1_^′^→E^′^ transition is allowed (Figure 4a). A true *D*_3h_ geometry is obviously not possible for the [**1**]^3+^ cation; nonetheless, the nine Eu–N interactions appear to be distributed sufficiently close to *D*_3h_ that the 4f electrons experience it as such. The closeness of the geometry to *D*_3h_ can be shown by finding the root-mean-square deviation of the central EuN_9_ atoms before and after application of each *D*_3h_ symmetry operation (SI Section S4.2): the deviations were ≤0.14 Å for the proper operations (*E*, 2*C*_3_, 3*C*_2_^′^) and ≤0.37 Å for the improper ones (σ_*h*_, 2*S*_3_, 3σ_*v*_), indicating close local adherence to *D*_3h_ despite the lower symmetry of the broader molecule.

**Figure 4:**
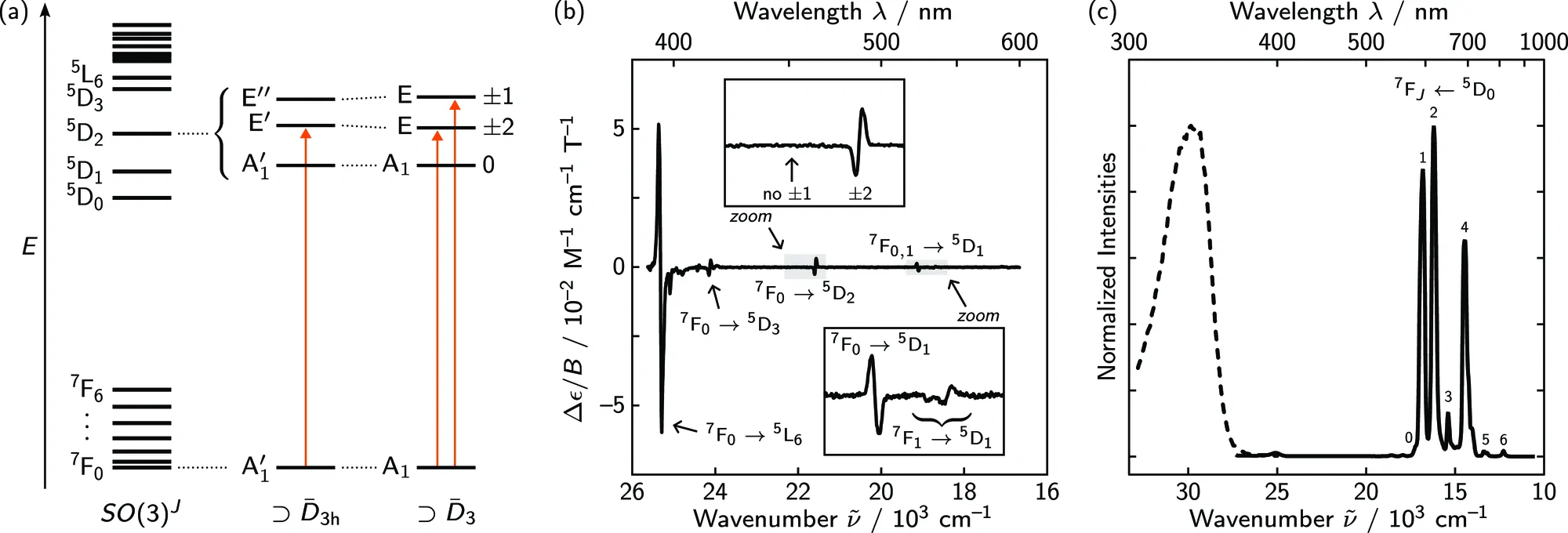
(a) The electronic structure of a free lanthanide ion like Eu^III^ is usually described using Lie symmetry groups,^40,41^ most importantly *SU*(2)^*S*^ ⊗ *SO*(3)^*L*^ ⊃ *SO*(3)^*J*^.^33^ The crystal field (CF) splits these states (CF splitting not to scale) according to irreducible representations (irreps) of the double group. Here, the ${\mathrm{D̅}}_{3h}$ double group is shown, where there is only one allowed transition (orange arrow) within the ^5^D_2_ state. Descent in symmetry to ${\mathrm{D̅}}_{3}$ yields two allowed transitions; thus, the ^7^F_0_→^5^D_2_ transition provides direct symmetry insight. (b) The room temperature MCD spectrum of [**1**] ${\left[{PF}_{6}\right]}_{3}$ in MeCN shows multiple $A_{1}$ -term features. The insets zoom into the ^7^F_0_→^5^D_2_ region and the ^7^F_0_→^5^D_1_ region, which also shows a ^7^F_1_→^5^D_1_ hot band. (c) The normalized emission (λ_exc_ = 356 nm, solid) and excitation (λ_em_ = 618 nm, dashed) spectra of [**1**] ${\left[{PF}_{6}\right]}_{3}$ in MeCN at room temperature.

The luminescence of [**1**] ${\left[{PF}_{6}\right]}_{3}$ in acetonitrile solution provided supporting evidence of the effective *D*_3h_ symmetry established by MCD. The complex exhibited characteristic orange-red emission matching that of [Eu­(tpy)_3_]­[ClO_4_],^47^ and the three most intense bands were assigned to be ^7^F_1_←^5^D_0_ at 16 807 cm^–1^, ^7^F_2_←^5^D_0_ at 16 207 cm^–1^, and ^7^F_4_←^5^D_0_ at 14 451 cm^–1^ (Figure 4c). In *D*_3h_ symmetry, the ^7^F_2_←^5^D_0_ transition is expected to comprise a single emission band rather than the two bands expected of *D*_3_ symmetry.^48^ The 16 207 cm^–1^ band assigned to this transition showed no splitting within the resolution of the experiment, consistent with the effective *D*_3h_ assignment.

### 3.3 Eu­(II): Spectroscopy and Magnetometry

#### 3.3.1 Room Temperature Spectroscopy

The room temperature absorption spectrum of [**1**]^2+^ in acetonitrile is dominated by a broad, relatively flat band spanning the entire visible region with an absorptivity around ϵ ∼ 1000 M^–1^ cm^–1^. The flat absorption feature is absent in the spectrum of [**1**]^3+^ and can therefore be ascribed to the Eu^2+^ center. This compound was not found to be luminescent in solution at room temperature or 77 K.

There are two types of possible 4f^7^→4f^6^[^7^F_*J*_]⊗ψ^1^ transitions that could make up this absorption feature (Figure 5a): 4f–5d transitions and metal-to-ligand charge transfer (MLCT) transitions. Both types of transitions can appear in the visible region. The lowest energy 4f–5d transition in the atomic Eu^2+^ ion appears around 34 000 cm^–1^;^27^ however, the strong-field ligand environment on the 5d orbitals is expected to lower this energy substantially,^49^ and it is not uncommon to see such transitions at or below 20 000 cm^–1^.^50^ We can estimate an approximate energy for an MLCT transition by comparing the redox potentials for the [**1**]^2/3^^+^ couple (−0.784 V vs Fc^0/+^, vide supra) with that of a substituted terpyridine (4′-*p*-tolylterpyridine −2.564 V vs Fc^0/+^ in DMF).^51^ The difference in potentials (1.78 V) suggests an MLCT feature could be observed as low as 14 000 cm^–1^. Both estimates are therefore consistent with the observed absorption spectrum.

**Figure 5:**
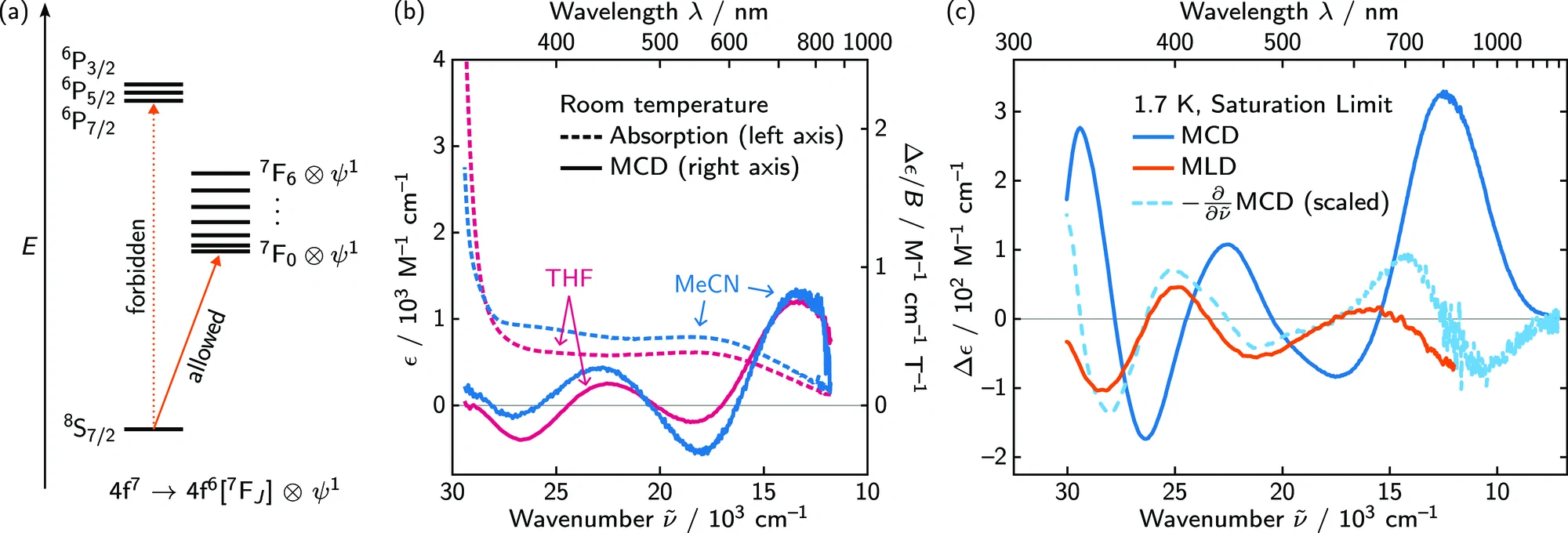
(a) The f–f transitions of Eu^II^ are high energy and forbidden (e.g., ^8^S_7/2_→^6^P_*J*_ is shown); instead, allowed 4f–5d (ψ = 5d) and MLCT (ψ = Lπ*) transitions dominate the absorption, MCD, and MLD spectra. After excitation, the residual 4f^6^ electrons will experience SOC-driven splitting into ^7^F_*J*_ states, which are not resolved in [**1**]^2+^ due to the large linewidths. Nonetheless, the presence of these *J* states has an impact on the appearance of the MCD and MLD spectra. (b) The room temperature absorption and MCD spectra of [**1**] ${\left[{BPh}_{4}\right]}_{2}$ are similar in THF and MeCN. A slight solvatochromic shift is revealed in the MCD spectra in the 350–500 nm region. (c) Cryogenic MCD and MLD spectra of [**1**]^2+^ in a frozen butyronitrile glass are shown after projection to the saturation limit. The MLD spectrum roughly follows the negative of the derivative of the MCD spectrum (shown dashed) except for the lowest energy feature, where there is significant deviation.

Room temperature MCD studies provided greater insight by showing that the broad absorption feature actually comprises several overlapping transitions (Figure 5b). To distinguish between 4f–5d and MLCT character, we measured the solvatochromism of the MCD features, as charge transfer transitions should show appreciable changes in energy with solvent polarity but metal-centered (like 4f–5d) transitions should not. The MCD features below 20 000 cm^–1^ (>500 nm) showed minimal change between THF (*E*_*T*_(30) = 37.4 kcal/mol) and acetonitrile (*E*_*T*_(30) = 45.6 kcal/mol),^52,53^ while a hypsochromic shift of ∼2000 cm^–1^ was seen for the features in the 20–28 000 cm^–1^ region upon switching to the more polar acetonitrile solvent. These results suggest the lower energy features are likely predominantly 4f–5d in character, while the higher energy features may be MLCT or mixed 4f–5d/MLCT in character.

#### 3.3.2 Cryogenic MCD and MLD Lineshapes

We acquired both MCD and MLD spectra (Figure 5c) on frozen glassy solutions of [**1**] ${\left[{BPh}_{4}\right]}_{2}$ in butyronitrile at 1.7 K. The MCD spectra showed a slight bathochromic shift relative to the room-temperature spectra but was otherwise qualitatively similar. Unexpectedly, the MLD spectra coarsely appeared to follow the negative derivative of the MCD spectra, an observation that prompted us to analyze the lineshapes of 4f^7^[^8^S]→4f^6^[^7^F_*J*_]⊗ψ^1^ transitions (*J* = 0–6, ψ = 5d or Lπ*) more closely.

While Eu^2+^ materials with narrow lineshapes are known to show individual staircase components for each *J* level,^29,32,54,55^ such features were not resolved here due to broad linewidths even at cryogenic temperatures (fwhm ∼ 3–4 000 cm^–1^). Because the line width meaningfully exceeds the splittings between *J* levels within the 4f^6^[^7^F_*J*_]⊗ψ^1^ progression, we can apply Taylor expansion. Assuming the levels are roughly split by $\zeta_{4f}\mathrm{L⃗}\cdot \mathrm{S⃗}$ , we start with

$$\frac{\left\langle \Delta \epsilon_{MCD}\right\rangle }{E}=\frac{\gamma \mu_{B}B}{k_{B}T}\overset{6}{\underset{J=0}{\sum }}C_{0}\left(J\right)f\left(E-\frac{\zeta_{4f}}{12}\left[J\left(J+1\right)-24\right]\right)$$ 13

$$\frac{\left\langle \Delta \epsilon_{MCD}\right\rangle }{E}=\frac{\gamma \mu_{B}B}{k_{B}T}\overset{6}{\underset{J=0}{\sum }}G_{0}\left(J\right)f\left(E-\frac{\zeta_{4f}}{12}\left[J\left(J+1\right)-24\right]\right)$$ 14

under nonsaturating conditions. Taylor expansion in ζ_4*f*_ and substitution of the values from Table 1 yields

15 $$\frac{\left\langle \Delta \epsilon_{MCD}\right\rangle }{E}=\frac{\gamma \mu_{B}B}{k_{B}T}\left(-147D_{0}\left(0\right)\zeta_{4f}\right)\left(-f^{\prime }\left(E\right)\right)$$

$$\frac{\left\langle \Delta \epsilon_{MLD}\right\rangle }{E}=\frac{\gamma \mu_{B}^{2}B^{2}}{k_{B}^{2}T^{2}}\left(-\frac{441}{8}D_{0}\left(0\right)\zeta_{4f}^{2}\right)\left(\frac{1}{2}f^{\prime \prime }\left(E\right)\right)$$ 16

These equations establish that even when individual staircase components are unresolved, the 4f^6^[^7^F_*J*_]⊗ψ^1^ progression still impacts the spectra through the formation of what is called a “pseudo- $A_{1}$ ” MCD signal and a corresponding “pseudo- $A_{2}$ ” MLD signal (Figure 6). The “pseudo” designation indicates that these lineshapes arise from the $C_{0}$ and $G_{0}$ intensity mechanisms rather than the $A_{1}$ and $A_{2}$ mechanisms normally associated with these first- and second-derivative features. This is the origin of the derivative-like relationship between the observed MCD and MLD spectra.

**Figure 6:**
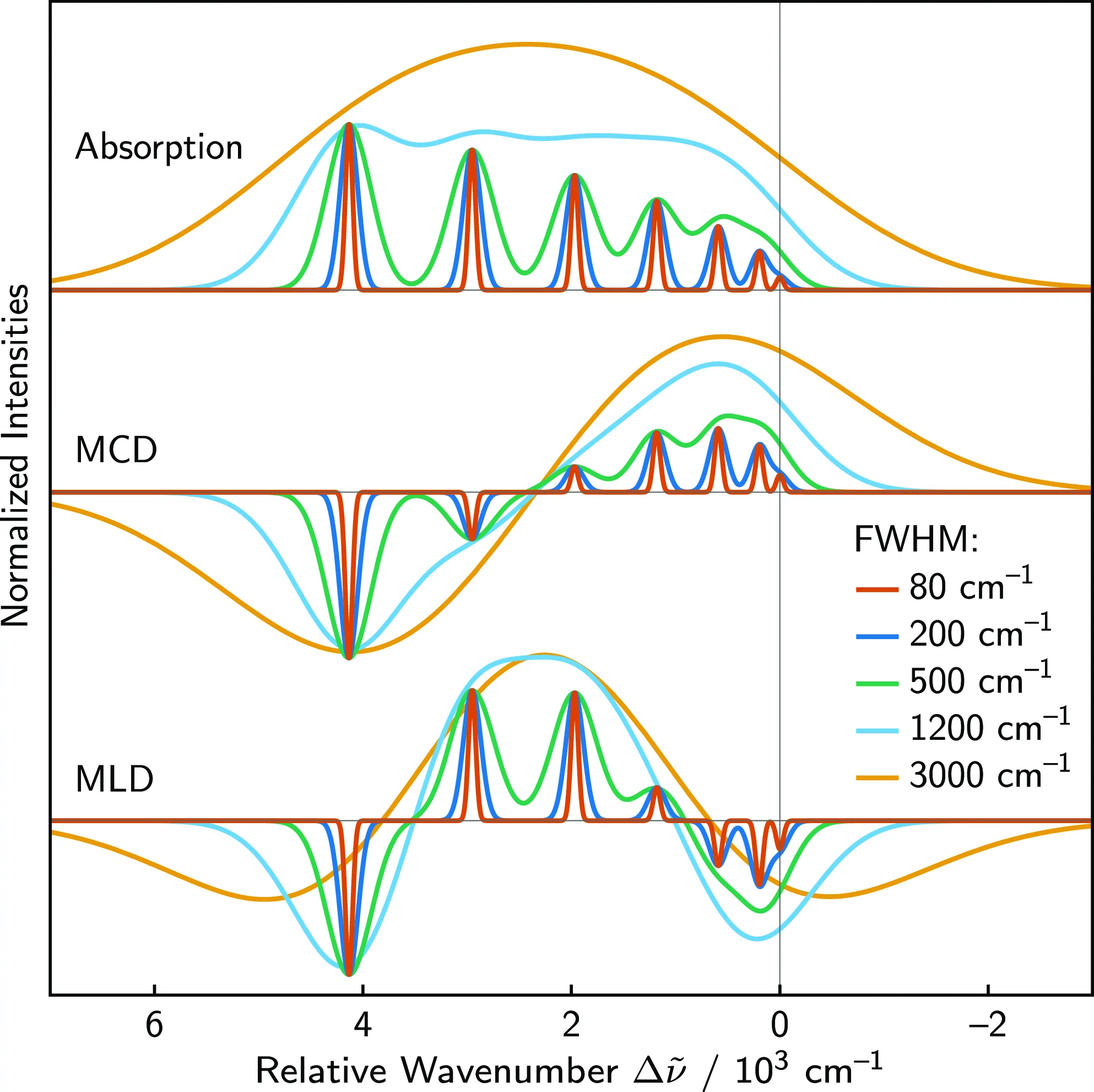
Observation of an underlying staircase pattern for a 4f^7^→4f^6^[^7^F_*J*_]⊗ψ^1^ transition is possible when the line width is sufficiently small. This is demonstrated here using Gaussian lineshapes of increasing full-width-at-half-maximum (fwhm) values and with intensities described by Table 1. The *x* axis shows the relative energy versus the ^7^F_0_⊗ψ^1^ level. As the fwhm gets larger, the features blur, causing a broad and flat absorption feature when the fwhm is similar to the Eu^2+^ SOC constant (ζ = 1181 cm^–1^ in the 4f^6^5d^1^ excited state)^27^ and then becoming roughly Gaussian in shape at higher fwhm. Large fwhm values cause the MCD and MLD spectra to show “pseudo- $A_{1}$ ” and “pseudo- $A_{2}$ ” features, respectively.

Our pseudo- $A_{1}$ /pseudo- $A_{2}$ framework describes most of the observed spectral features well, but the largest deviation is observed in the lowest energy feature below 15 000 cm^–1^. The MLD spectrum noticeably deviates from the derivative of the MCD spectrum, and the MCD of this feature shows a positive lobe that is far larger than the negative lobe. The asymmetry of the MCD feature reveals a breakdown in the assumptions underlying eqs 15 and 16. Most likely, this indicates that the transition is experiencing non-negligible SOC with a nearby state, something that was explicitly neglected when deriving Table 1. Strong SOC is consistent with a 4f–5d transition, a conclusion consistent with the absence of room temperature solvatochromism observed for this feature. A second noteworthy deviation from the idealized model of Figure 6 appears in the room temperature data of Figure 5b, where the MCD crossing points do not align exactly with absorption maxima; this is likely a consequence of not only SOC but also the superposition of several overlapping transition envelopes caused by the broadness of the room temperature features.

#### 3.3.3 Magnetometry

VTVH MCD and MLD measurements of [**1**]^2+^ confirmed that the intensities of the observed features scale with the saturation expressions in eqs 10 and 11 (Figure 7a,b). These VTVH MCD and MLD behaviors conclusively demonstrate that the signals arise exclusively from overlapping $C_{0}$ (MCD) and $G_{0}$ (MLD) term contributions, as any alternative Faraday parameter intensity mechanisms would produce characteristically different field and temperature dependences. The VTVH data therefore also confirm a lack of appreciable ZFS by magneto-optical spectroscopy as indicated by the lack of “nesting” behavior—a term that describes the spreading of saturation curves across different temperatures.^56^ To the best of our knowledge, this is the first observation of $G_{0}$ MLD signal in any f-block molecular complex.

**Figure 7:**
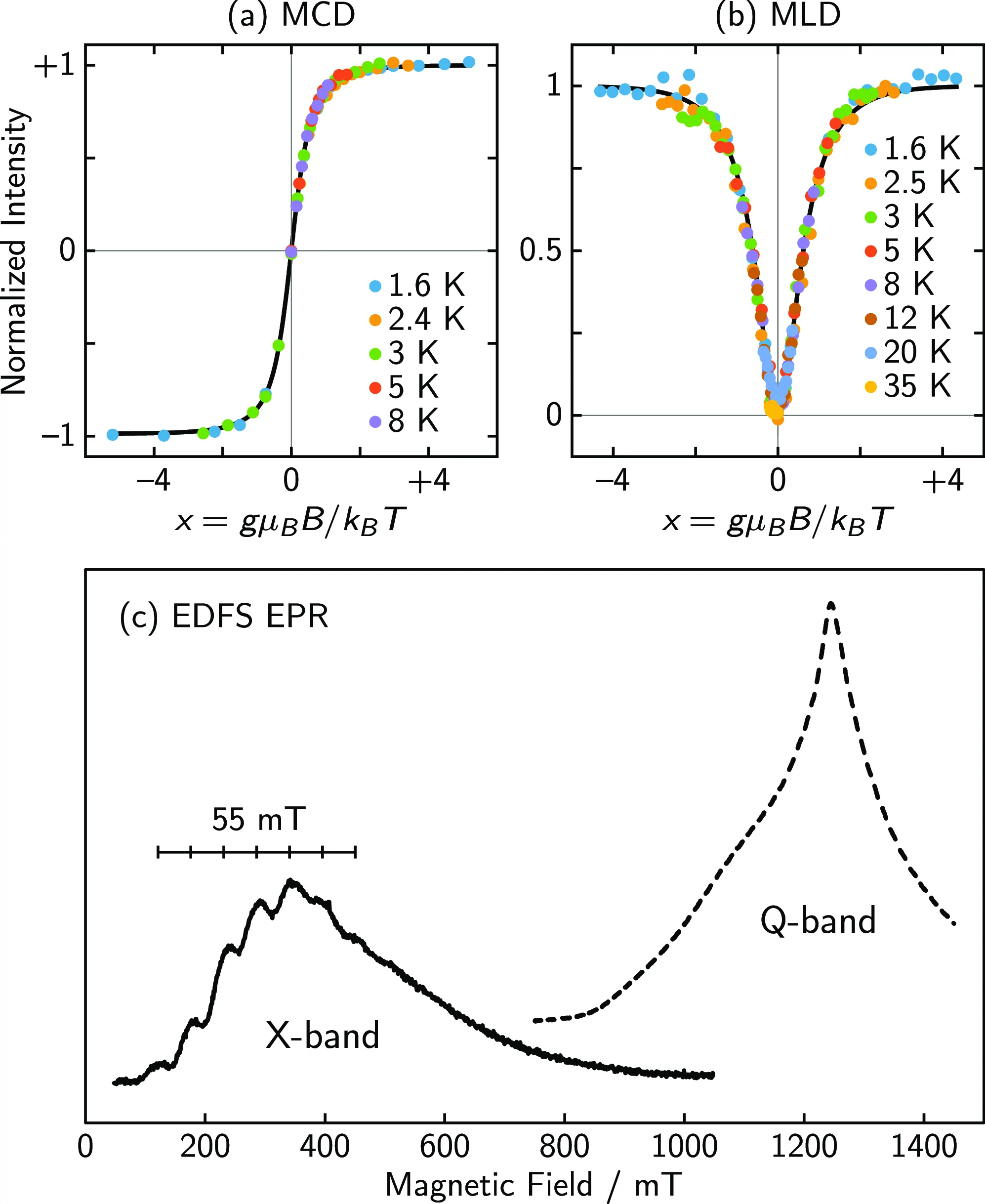
Effects of magnetic saturation in the (a) VTVH MCD and (b) VTVH MLD measurements are well modeled by eqs 10 and 11, respectively (shown in black using *g* = *g*_*e*_ = 2.0023). (c) The echo-detected field-swept (EDFS) EPR spectra of [**1**]­ ${\left[{BPh}_{4}\right]}_{2}$ in butyronitrile were acquired at 5 K. The X-band spectrum shows seven peaks roughly spaced by 55 mT (corresponding to a ZFS of *D* ∼0.052 cm^–1^ or ∼1.54 GHz).

The presence of $G_{0}$ MLD signal itself requires a ground state with three or more energetically proximal magnetic sublevels. This condition is satisfied by the ^8^S_7/2_ ground states of Eu^2+^ and Gd^3+^ ions, which tend to have small ZFS (*D*). X-band and Q-band EPR spectroscopy supported the near-zero *D* that was suggested by our VTVH MCD/MLD measurements for [**1**]^2+^. The X-band echo-detected field-swept (EDFS) spectrum at 5 K in butyronitrile (Figure 7c) revealed a series of seven peaks evenly spaced by ∼55 mT. This seven-line pattern is consistent with ZFS splitting for an S = 7/2 ion of *D* ∼ 0.052 cm^–1^ (∼1.54 GHz) or equivalently *B*_2_^0^ ∼ 0.017 cm^–1^ (∼0.51 GHz). This value is similar in order of magnitude to other Eu­(II) centers,^57,58^ and is smaller than can be reasonably resolved through VTVH MCD/MLD magnetometry.

### 3.4 Neutral Europium Complex: Ligand Non-Innocence

Formulation of neutral [**1**]^0^ as a true Eu(0) compound is chemically unreasonable, so it is clear that the terpyridine ligands are engaging with the metal in a redox noninnocent manner. A redox noninnocent description assigns radical anion character to one or more of the terpyridine ligands, which should be reflected in the bond metrics, magnetism, and spectroscopic signatures of the molecule.

Crystallographic metrics (Figure 3d) revealed contracted C_py_–C_py_ distances and flattened N–C–C–N torsional angles, both consistent with partial reduction of the terpyridine ligands, and thus an interpretation of this compound as [Eu^II^(mtpy)­(mtpy^•–^)_2_]. Its magnetic moment measured by the Evans method, μ_eff_ = 6.02(10) μ_B_, provided further insight into the spin state. An S = 5/2 spin state, representing both two organic mtpy^•–^ radicals each antiferromagnetically coupled to the Eu­(II) center, should exhibit μ_eff_ = 5.92 μ_B_, which lies within one standard deviation from the measured value. Other possible spin states can be ruled out: a ferromagnetically coupled S = 9/2 system would give μ_eff_ = 9.96 μ_B_ and an intermediate S = 7/2 spin system would give μ_eff_ = 7.95 μ_B_, both inconsistent with our measurement.

The (magneto-)­optical spectra of neutral [**1**]^0^ also reflect the presence of redox noninnocent terpyridine ligands; however, complete spectroscopic analysis and VTVH MCD/MLD magnetometry were prevented by rapid reaction of [**1**]^0^ with the O-rings of our cryogenic sample holder. Thus, we were only able to obtain room temperature absorption and MCD spectra. These spectra were interpreted through comparison (Figure 8) with [**1**]^2+^ and with K­(mtpy), an independently synthesized sample of the 4′-mesitylterpyridine radical anion (Supporting Information Section S1.6). Relative to [**1**]^2+^, the absorption spectrum of the neutral complex shows prominent new features around 16 000, 22 000, and 24–27 000 cm^–1^, the lowest two of which appear at energies similar to features present in the K­(mtpy) spectrum. We tentatively assign these lowest two features as carrying substantial intraligand character. This assignment rests primarily on their large absorptivities (ϵ ∼ 10^4^ M^–1^ cm^–1^) paired with comparatively weak MCD signals (Δϵ ∼ 1 M^–1^ cm^–1^ T^–1^), a mismatch that is characteristic of transitions with diminished metal character. We note that these features are far weaker in the MCD spectrum of K­(mtpy); their comparative prominence in [**1**]^0^ could reflect either Eu^2+^ character mixed into these transitions in the complex that is absent in K­(mtpy), or an underlying Eu^2+^-centered transition that is obscured in the congested absorption spectrum. A slight bathochromic shift in the MCD feature near 16 000 cm^–1^ upon switching from toluene to THF (*E*_*T*_(30) = 33.9 and 37.4 kcal/mol, respectively)^59^ indicates some degree of charge transfer character associated with these largely radical ligand-based transitions (SI Figure S10).

**Figure 8:**
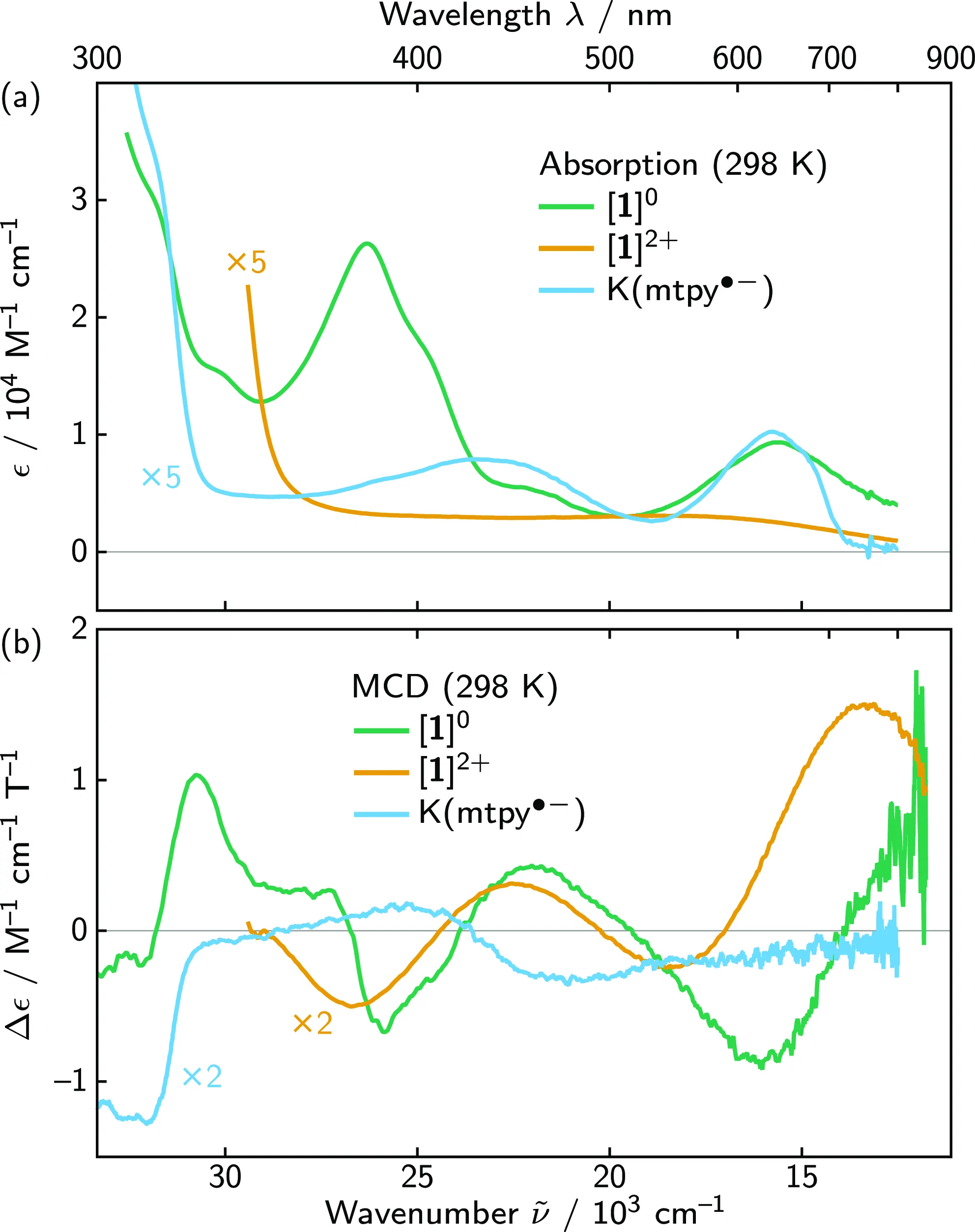
Absorbance (a) and MCD (b) spectra of [**1**] (green), [**1**] ${\left[{BPh}_{4}\right]}_{2}$ (orange), and K­(mtpy) (blue) in THF at ambient temperature. Spectra of [**1**] ${\left[{BPh}_{4}\right]}_{2}$ and K­(mtpy) were magnified.

## 4 Conclusion

We have prepared a series of homoleptic terpyridine complexes of europium in three formal oxidation states and characterized them by magneto-optical spectroscopy. For the Eu­(III) complex, MCD spectroscopy revealed an effective *D*_3h_ local symmetry around the metal center that is higher than the *D*_3_ geometry observed crystallographically. The number of $A_{1}$ -term features resolved in the ^7^F_0_→^5^D_2_ region provides this assignment directly, extending a long-established use of Eu­(III) MCD for site-symmetry determination by Görller-Walrand and co-workers.^4,43–46^ Our work contrasts with these earlier studies by revealing an effective local symmetry that differs from the broader molecular symmetry. In this way, our MCD analysis of f–f transitions provides insight into the impact of local symmetry on electronic structure that is inaccessible through crystallography and most other solution-state techniques.

For the Eu­(II) complex, we developed theoretical expressions for the MCD and MLD responses of unresolved 4f^7^→4f^6^[^7^F_*J*_]⊗ψ^1^ progressions and showed that this framework accounts for most of the observed spectral intensity. Deviations at low energy are consistent with interstate SOC within the 4f–5d excited state manifold. VTVH measurements of both MCD and MLD intensities confirmed the ZFS of the ^8^S_7/2_ ground state of the [**1**]^2+^ cation is near-zero, and to the best of our knowledge these measurements constitute the first observation of $G_{0}$ MLD signals in any f-block molecular complex. A prior MCD study of Ln­(II) complexes has shown that transitions within the 4f^*N*^ and 4f^*N*–1^5d^1^ manifolds span the UV–vis–NIR region,^60^ and we expect that the joint MCD/MLD methodology explored here could prove useful in assigning such features. Our neutral [**1**]^0^ complex has properties most consistent with a [Eu^II^(mtpy)­(mtpy^•–^)_2_] formulation, and thus adds terpyridine to the library of redox noninnocent ligands known within the f block.^35,61–65^

Together, our magneto-optical characterizations of [**1**]^3+^, [**1**]^2+^, and [**1**]^0^ demonstrate the power of MCD and MLD spectroscopy as complementary tools for probing f-block electronic structure in solution. Combined UV–vis–NIR MCD and MLD spectroscopy may be particularly useful techniques when probing lanthanide ions with unresolved or overlapping ^2*S*+1^ *L*_*J*_ levels, as the different lineshapes can provide insight into the nature of the transitions. These techniques connect with the growing body of literature on related (magneto-)­chiroptical properties (circularly polarized luminescence, magnetochiral dichroism)^66–73^ of the f-block, whose distinct sensitivities to molecular chirality can augment the symmetry and magnetic information accessed by MCD and MLD in chiral materials.

## 5 Experimental Section

Absorption, MCD, and MLD spectra were acquired using a JASCO J-1700 spectrophotometer equipped with a S-20 photocathode-equipped photomultiplier tube (PMT) detector (λ = 163–950 nm, JASCO Model PM-539), a S-1 photocathode-equipped PMT detector (λ = 400–1250 nm, JASCO Model EXPM-531), or an InGaAs detector (λ = 800–1600 nm JASCO Model EXIG-542 or λ = 1600–2500 nm JASCO Model EXIG-543). Emission and excitation spectra of [**1**] ${\left[{PF}_{6}\right]}_{3}$ (6.47 μM in MeCN) were collected with a Jobin Yvon-Horiba Fluoromax 3 spectrometer. Compounds analyzed were synthesized as described in the Supporting Information document.

Room temperature MCD data were acquired using a JASCO MCD-581 electromagnet (|*B*| ≤ 1.5 T). Samples held within the electromagnet were generally found to be 20 ± 2 °C. Cuvettes were purchased from Spectrocell (10 mm “NIR-grade” quartz cells, 220–3500 nm range) and were adapted to have a Young’s valve to allow analysis of air-free samples. MCD analysis of each cuvette was performed to ensure there were no artifacts introduced during the glassblowing process of attaching this valve. The J-1700 instrument is a single-beam spectrometer, so solvent blanks needed to be subtracted from absorption data. To do so, room temperature absorption spectra were acquired using identical collection parameters immediately after collecting the absorption spectrum of the sample and these data were subtracted to yield the final data.

Cryogenic MCD and MLD data were acquired using an Oxford SpectromagPT magnet (T = 1.7–300 K, |*B*| ≤ 7 T) equipped with four Suprasil windows. The JASCO J-1700 spectrometer and detector were positioned on opposite sides of the magnet, either parallel to the direction of the field (MCD) or perpendicular to the direction of the field (MLD). The detector was placed outside the 50 G line. Three planoconvex CaF_2_ lenses (ThorLabs) were used to focus light between the spectrometer and the detector, focusing the light on the sample and taking care to ensure that any cracks in the frozen glass were avoided. The sample of interest was sandwiched between two quartz windows (Spectrocell NIR quartz, 1 cm diameter) using a fluorosilicone O-ring to contain the solution, then flash-frozen in a liquid nitrogen bath. Solvents were chosen to ensure formation of an optically transparent glass. Glasses were inspected after freezing to ensure light could be focused through the sample without passing through any cracks. Samples of sufficient quality were loaded into the magnet cold (∼80 K), ensuring that the sample never warmed near/above the glass transition temperature.

## Supplementary Material



## Notes

Synthesis information, NMR spectra, additional UV–vis–NIR absorbance and MCD/MLD spectra, EPR spectra, X-ray crystallographic data, electrochemical data, theoretical derivation of MLD saturation equation, and detailed MCD/MLD data processing information. (PDF)
