# Supplementary material for: Magneto-Optical Spectroscopy Across Three Oxidation States of a Europium Terpyridine Complex
Source: Inorg Chem. 2026 Aug 18;65(34):19799–810. doi: 10.1021/acs.inorgchem.6c02413 (PMC13557445; doi:10.1021/acs.inorgchem.6c02413)
Supplement: Supplementary file 1 [file ic6c02413_si_001.pdf]

Supporting Information

Magneto-Optical Spectroscopy Across Three Oxidation States of a Europium Terpyridine Complex

Che Wu, Elizabeth L. Fosnocht, Nicholas A. Moriglioni, Wesley J. Transue*

Department of Chemistry, University of Pittsburgh, Pittsburgh, PA 15213, USA

**Email: wtransue@pitt.edu*

Contents

S1 Synthesis **S2**

S1.1 General Remarks . . . S2

S1.2 Synthesis of $[Eu(mtpy)_3][PF_6]_3 \cdot MeCN$ ($[\mathbf{1}][PF_6]_3 \cdot MeCN$) . . . S2

S1.3 Synthesis of $Eu(mtpy)_2I_2 \cdot 2MeCN$ . . . S2

S1.4 Synthesis of $[Eu(mtpy)_3][BPh_4]_2 \cdot 2THF \cdot Et_2O$ ($[\mathbf{1}][BPh_4]_2 \cdot 2THF \cdot Et_2O$) . . . S3

S1.5 Synthesis of $Eu(mtpy)_3 \cdot 2PhMe$ ($[\mathbf{1}]^0 \cdot 2PhMe$) . . . S3

S1.6 Synthesis of K(mtpy) . . . S5

S2 MCD and MLD Spectroscopy **S7**

S2.1 MLD $\mathcal{G}_0$ Saturation Behavior . . . S7

S2.2 Room Temperature Data Collection and Processing . . . S8

S2.3 Cryogenic Data Collection and Processing . . . S8

S3 Electron Paramagnetic Resonance **S13**

S3.1 Pulsed EPR Experiments . . . S13

S3.2 Continuous Wave (CW) EPR Experiment on K(mtpy) . . . S14

S4 X-ray Crystallography **S15**

S4.1 Refinement Details . . . S15

S4.2 Symmetry Analysis . . . S18

S5 Electrochemistry **S21**

S1 Synthesis

S1.1 General Remarks

Unless specified, all manipulations were performed in an MBraun LabMaster Pro SP glovebox under an inert atmosphere of purified N_2 or using standard air-free Schlenk techniques. Hexanes, acetonitrile, toluene, Et_2O, and THF were obtained anhydrous and oxygen-free by bubble degassing (Ar), purification through alumina columns (Pure Process Technology Solvent Purification System). Butyronitrile was heated at 75 °C thrice for 3–4 h over Na_2CO_3 and $KMnO_4$ (0.77 g and 1.15 g per 100 mL solvent, respectively) under Ar to remove isonitriles;[1] it was vacuum distilled over $KMnO_4$ and degassed by freeze–pump–thaw cycles. 2-Methyltetrahydrofuran was stirred over excess Na/benzophenone at ambient temperature overnight before being vacuum distilled and degassed by freeze–pump–thaw cycles. All solvents, protic and deuterated, were stored in the glovebox over activated 3 Å molecular sieves for at least 2 days prior to use. Glassware was oven dried for at least 2 h at temperatures greater than 150 °C.

Silver hexafluorophosphate (TCI America), $EuCl_3{\cdot}6H_2O$, and sodium tetraphenylborate (Thermo Scientific) were purchased and used as received. Potassium graphite (KC_8),[2] 4′-mesityl-2,2′:6′,2″-terpyridine (mtpy),[3] and $EuI_2(THF)_2$[4,5] were synthesized according to literature procedures. Prior to use, mtpy was repeatedly recrystallized from boiling methanol until a white crystalline product was obtained.

All NMR experiments were conducted using a Bruker Avance III 400 MHz instrument. ^{1}H NMR chemical shifts (δ) are reported in parts per million (ppm) relative to tetramethylsilane, using residual proton signals from the deuterated solvent as an internal reference.[6] Evans method measurements on $[\mathbf{1}]^0{\cdot}2PhMe$ and $[\mathbf{1}][BPh_4]_2{\cdot}2THF{\cdot}Et_2O$ were conducted following literature procedures.[7,8] Initial samples were prepared using recrystallized materials and corresponding NMR solvents (d6-benzene for $[\mathbf{1}]^0{\cdot}2PhMe$; d3-MeCN for $[\mathbf{1}][BPh_4]_2{\cdot}2THF{\cdot}Et_2O$). Replicate measurements were done on samples obtained by diluting the initial sample. Frequency shifts were measured using residual proton signals of NMR solvents. Molar susceptibilities were corrected using Pascal's constants.[9] Temperature of data collection was assumed to be 300 K. Under these circumstances, the measured μ_{eff} for $[\mathbf{1}][BPh_4]_2{\cdot}2THF{\cdot}Et_2O$ at ambient temperature is $7.52(39)\mu_B$.

S1.2 Synthesis of $[Eu(mtpy)_3][PF_6]_3{\cdot}MeCN$ ($[\mathbf{1}][PF_6]_3{\cdot}MeCN$)

In a 20 mL scintillation vial, $EuCl_3{\cdot}6H_2O$ (0.0577 g, 0.158 mmol) and mtpy (0.174 g, 0.494 mmol) were combined as solids in open air. To the mixture, 5 mL MeCN was added, and the mixture was heated for 10 min till boiling, during which time the solids dissolved. To the warm solution, $AgPF_6$ (0.138 g, 0.547 mmol) was added as as a 3 mL MeCN solution. White precipitates developed immediately, and the mixture was then left to stir at room temperature overnight. The reaction mixture was filtered; the colorless filtrate was concentrated to 2 mL before being layered with 6 mL Et_2O. After ∼2 days, the diffusion yielded colorless crystals, among which X-ray quality samples were found. The product was isolated as a fine colorless crystals after recrystallizing twice from MeCN/Et_2O and washing the crystals with 2×5 mL THF and 3×3 mL Et_2O (0.184 g, 71.1% yield). ^{1}H NMR (400 MHz, d3-MeCN, Figs. S1–S2) δ 16.96 (s, 6H, *H3*), 6.85 (t, $J = 7.6$ Hz, 6H, *H5*), 6.57 (s, 6H, *mesityl CH*), 6.21 (d, $J = 7.6$ Hz, 6H, *H4*), 2.11 (s, 6H, *H3′*), 2.08 (d, $J = 8.1$ Hz, 6H, *H6*), 2.00 (s, 9H, *mesityl p-CH$_3$*), 0.93 (s, 18H, *mesityl o-CH$_3$*).

S1.3 Synthesis of $Eu(mtpy)_2I_2{\cdot}2MeCN$

In a 20 mL scintillation vial, $EuI_2(THF)_2$ (0.103 g, 0.188 mmol) was combined with 3 mL MeCN. To the mixture, mtpy (0.137 g, 0.389 mmol) was added as solids. The reaction mixture changed from bright yellow to deep reddish purple upon the addition of mtpy, and precipitates emerged within 10 min. The mixture was allowed to stir at room temperature overnight before being filtered. The precipitates were dissolved in MeCN and layered with three volumes of Et_2O, yielding 0.118 g product as deep purple crystals. Layering the filtrate with three times the volume of Et_2O yielded additional crude products, which, upon recrystallization using the same condition, yielded 0.069 g product (0.184 g in total, 82.0% yield). X-ray quality crystals were grown through layered diffusion of Et_2O into the MeCN solution of the product.

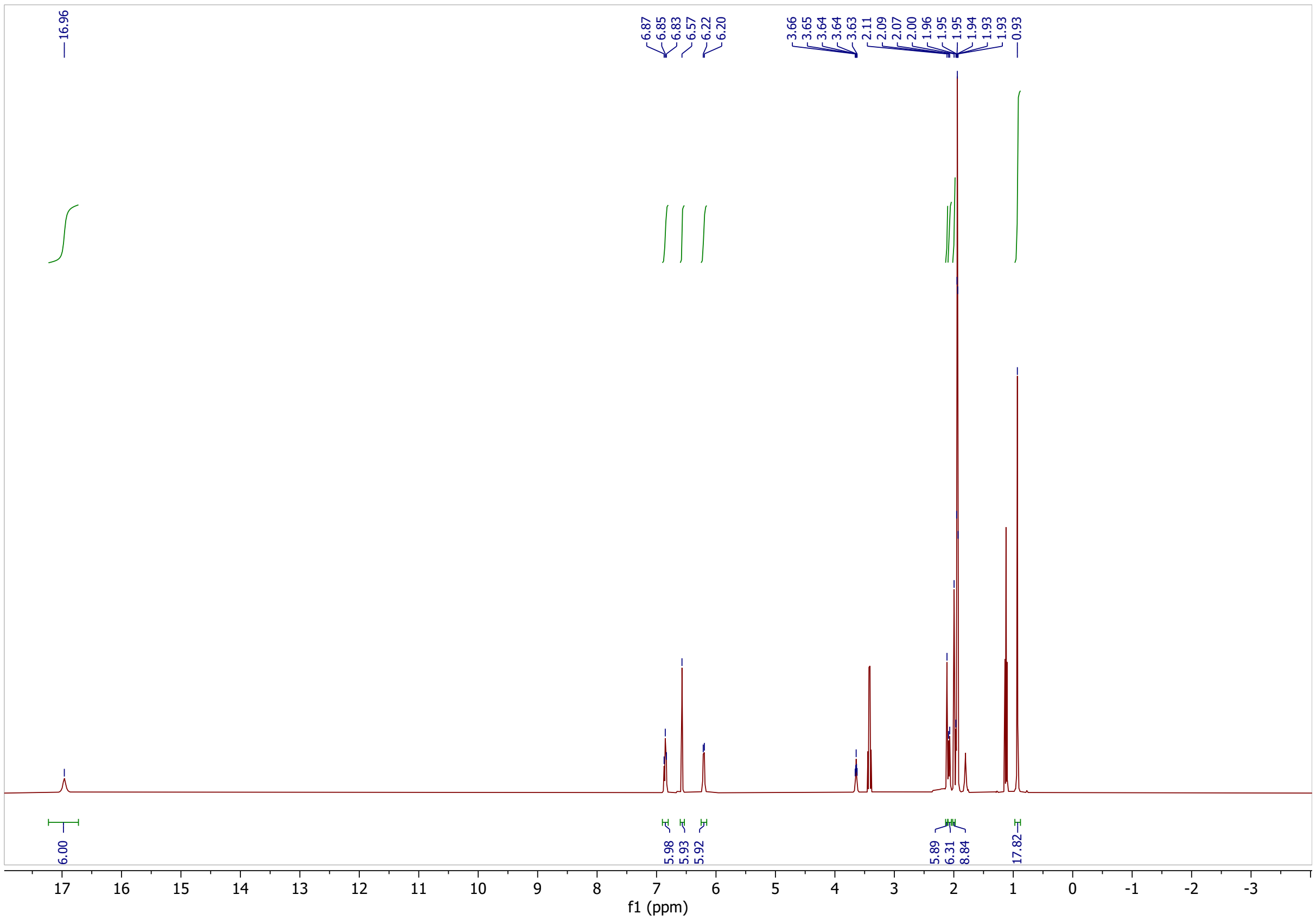

Figure S1: ^{1}H NMR spectrum of [**1**]$[PF_6]_3$·MeCN in d3-acetonitrile.

S1.4 Synthesis of $[Eu(mtpy)_3][BPh_4]_2$·2THF·Et_2O ([1]$[BPh_4]_2$·2THF·Et_2O)

In a 20 mL scintillation vial, $(mtpy)_2EuI_2$·MeCN (0.0650 g, 0.0546 mmol)and mtpy (0.0250 g, 0.0711 mmol) was combined as solids. To the mixture, 2 mL THF was added. The white mtpy crystals completely dissolved while the purple crystals of $(mtpy)_2EuI_2$·MeCN only partly dissolved, yielding a light purple liquid phase. To the mixture, $NaBPh_4$ (0.0411 g, 0.120 mmol) was added as solids. Upon the addition of $NaBPh_4$, the color of the liquid phase intensified immediately. The reaction mixture was then left to stir at room temperature overnight before being filtered. The isolated precipitates were exhaustively extracted with THF until the wash is no longer visibly colored. The collected liquid phase was concentrated to ∼3 mL and layered with 3× Et_2O, yielding deep purple crystalline solids. Further recrystallizations (thrice) yielded the product as purple crystalline powders (0.0670 g, 66.3% yield). X-ray quality crystals were grown by diffusing Et_2O vapor into the THF solution of the product. Alternatively, the compound can be prepared in the same route with $KBPh_4$ (2.2 equiv.) with one round of recrystallization (45.2% yield). ^{1}H NMR (400 MHz, d3-acetonitrile, Fig. S3) δ 7.26 (br s, 16H, BPh_4 anion *o*- or *m*-CH), 6.98 (br s, 16H, BPh_4 anion *o*- or *m*-CH), 6.83 (br s, 8H, BPh_4 anion *p*-CH), 2.58 (br s, anion). Only one resonance associated with the cation was found. It integrated to 7.12H when the sum of the $[BPh_4]_2$ resonances was set to 40H, but integrations of such broad features are heavily influenced by choices in baseline correction so this could plausibly be a 6H or 9H feature. Lacking observations of additional resonances, we do not make an assignment.

S1.5 Synthesis of $Eu(mtpy)_3$·2PhMe ([1]^0·2PhMe)

K(mtpy) route. In a 20 mL scintillation vial, a 2:1 K(mtpy)/mtpy mixture was prepared by adding potassium metal (0.0091 g, 0.23 mmol) to a solution of mtpy (0.116 g, 0.330 mmmol) in 3 mL THF. To the

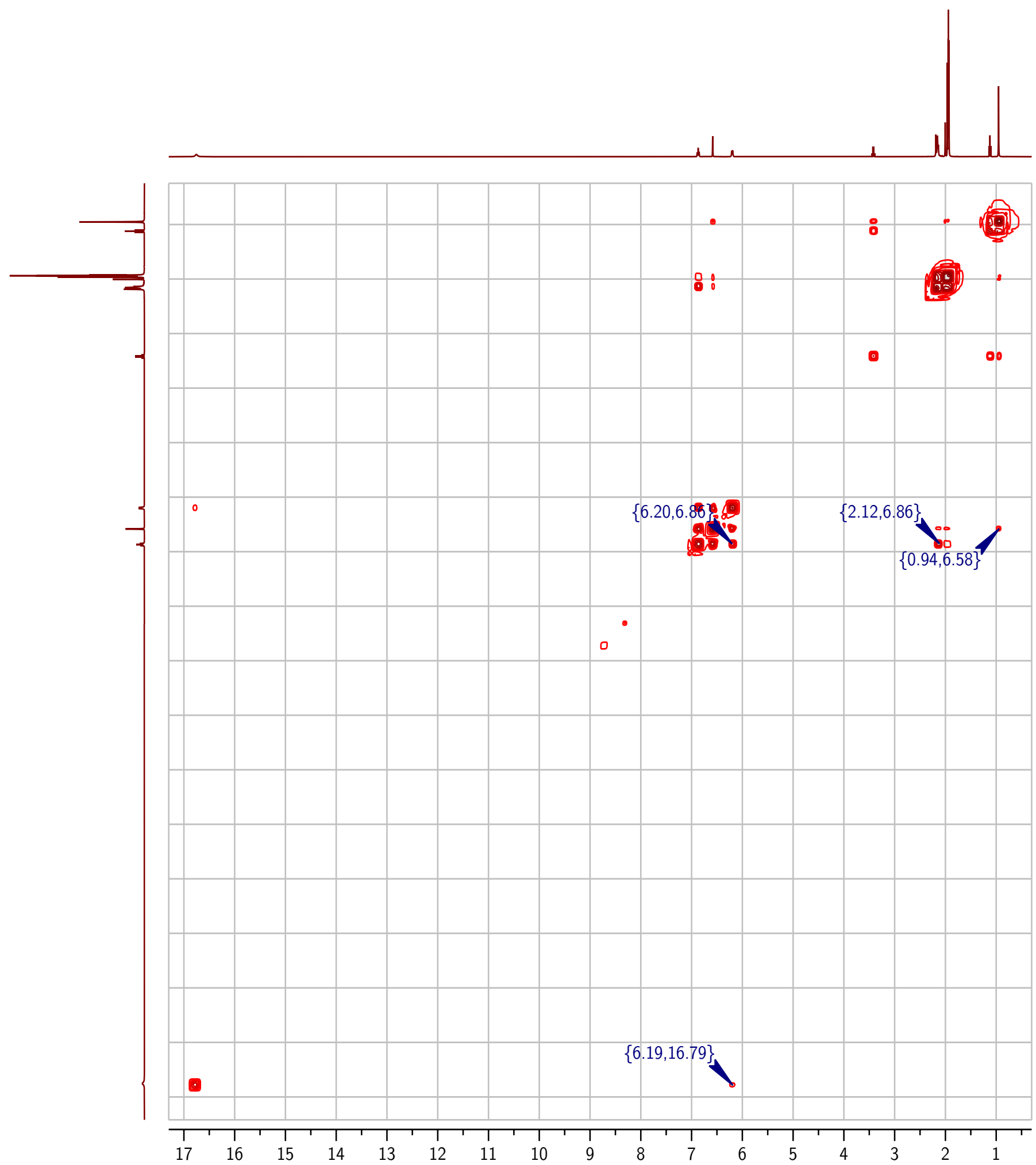

Figure S2: ^{1}H COSY NMR spectrum of [**1**]$[PF_6]_3$·MeCN in d3-acetonitrile.

mixture, $(THF)_2EuI_2$ (0.043 g, 0.078 mmol) was added as solids. The mixture was left to stir at ambient temperature for 2 h before being filtered. The filtrate was then dried in vacuo, and the resulting solid residue was repeated extracted first with Et_2O and then with toluene until the color of the extract became almost colorless. The combined extracts were then dried in vacuo. The solid residue was dissolved in minimal amount of THF (∼1 mL), which was layered with ∼16 mL hexane in darkness at −30 °C. Crystalline precipitates were collected as the crude product. Further purification was achieved by layer diffusion of hexanes into the near-saturated toluene solution of the crude product, leaving deep blue crystalline solids as the product (0.0253 g, 24% yield). ^{1}H NMR (400 MHz, d6-benzene, Fig. S4) δ 13.25 (br s, FWHM 18 Hz, 6H, *mesityl CH*), 6.49 (br s, FWHM 12 Hz, 9H, *mesityl p-CH$_3$*), 4.69 (br s, FWHM 22 Hz, 18H, *mesityl o-CH$_3$*). Only three resonances were observed and their integration of 1:1.5:3 led us to assign them as arising from the mesityl substituents.

Chemical reduction route. In a 20 mL scintillation vial, $[Eu(mtpy)_3][BPh_4]_2$·2THF·Et_2O (0.126 g, 0.0643 mmol) was combined with 3 mL THF. To the stirring mixture, KC_8 (0.0180 g, 0.133 mmol) was added as solids, and the mixture was left to stir in darkness at room temperature for 1 h. The reaction mixture was then filtered; the filter cake was washed extensively with THF until the wash was no longer colored. The combined THF solution was dried in vacuo. The solid residue was again washed with Et_2O and minimal amount of toluene until a very pale green filter cake was found. The filtrate was again dried in vacuo, and the solid residue was dissolved in minimal amount of THF and layered with hexanes at −30 °C, yielding dark

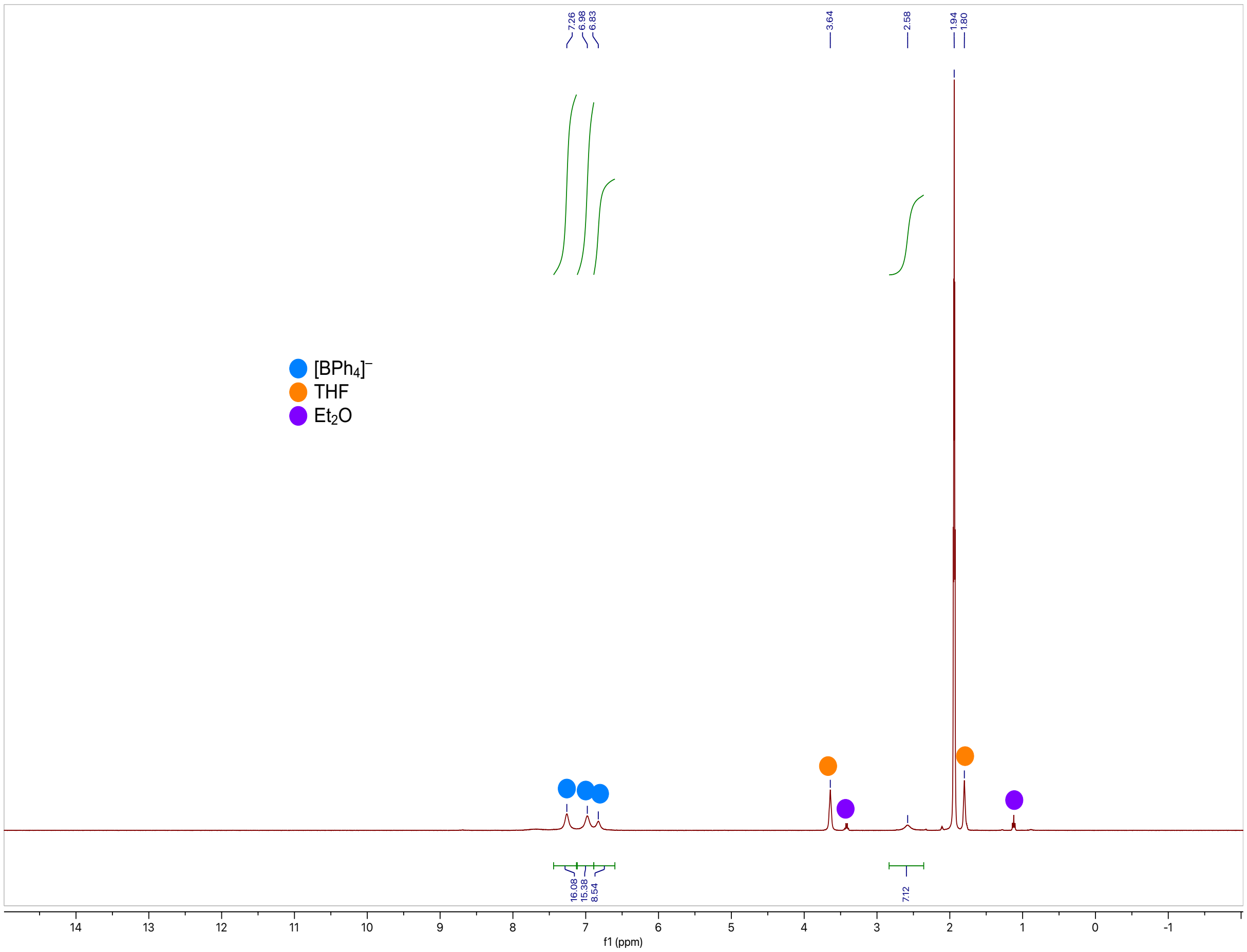

Figure S3: ^{1}H NMR spectrum of [**1**][BPh_4]$_2$·2THF·Et_2O in d3-acetonitrile.

crystalline solids as the crude product. The final product was obtained as deep blue crystals through layered diffusion of hexane into the near-saturated solution of the crude product in toluene at −30 °C (0.0421 g, 47.1% yield). X-ray quality single crystal samples were obtained in the same manner.

S1.6 Synthesis of K(mtpy)

In a 20 mL scintillation vial, potassium (0.0138 g, 0.353 mmol) was added to a 2 mL THF solution of mtpy (0.120 g, 0.340 mmol). The reaction mixture turned green immediately. After ∼30 min, the potassium piece almost completely dissolved while the reaction mixture turned turbid. The reaction mixture was diluted to ∼10 mL using THF and filtered. Drying the filtrate in vacuo yielded deep green solids as the product (0.118 g, 99.2% yield).

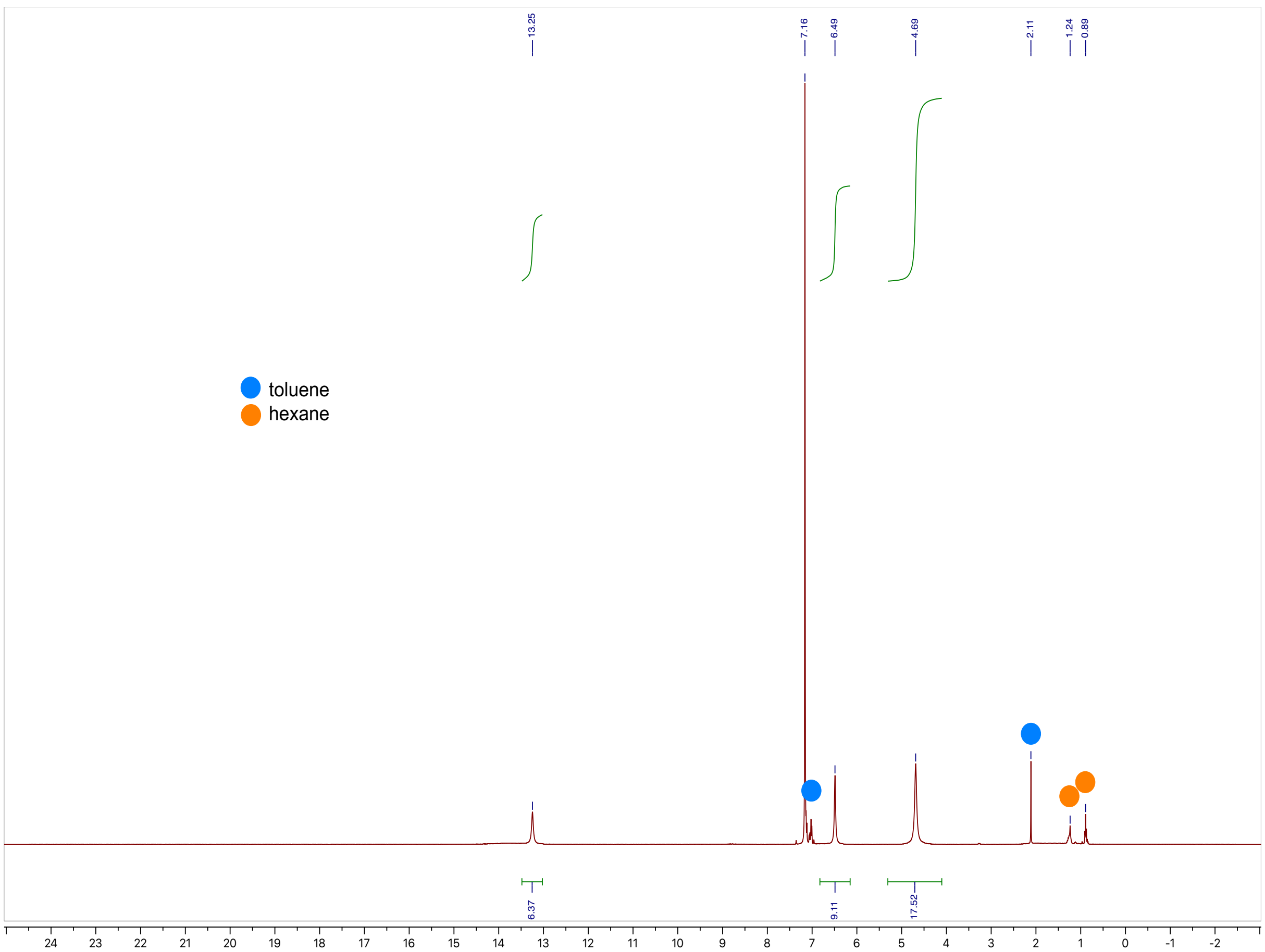

Figure S4: ^{1}H NMR spectrum of $[\mathbf{1}]^0 \cdot 2PhMe$ in d6-benzene.

S2 MCD and MLD Spectroscopy

S2.1 MLD $\mathcal{G}_0$ Saturation Behavior

To model a system with an isotropic magnetic response, we consider a system with an isotropic ground state g value that lacks any zero-field splitting. For simplicity, an atomic ${}^{2S+1}\mathrm{S} \rightarrow {}^{2S+1}\mathrm{P}_J$ transition meets these criteria so we will analyze this system. We will indicate levels as $|LM_LSM_S\rangle$ or $|LSJM_J\rangle$, depending on whether we are in the uncoupled or coupled basis, respectively. The transition dipole moment between these two levels can be calculated using the Wigner–Eckart theorem to be

$$\begin{aligned}
\langle 00SM_S|m_q^1|1SJM_J\rangle &= \sum_{\bar{M}_L\bar{M}_S} \left\langle 00SM_S\middle|m_q^1\middle|1\bar{M}_LS\bar{M}_S\right\rangle \left\langle 1\bar{M}_LS\bar{M}_S\middle|JM_J\right\rangle \\
&= \sum_{\bar{M}_L} \left\langle 00\middle|m_q^1\middle|1\bar{M}_L\right\rangle \left\langle 1\bar{M}_LSM_S\middle|JM_J\right\rangle \\
&= \sum_{\bar{M}_L} \left\langle 1\bar{M}_L1q\middle|00\right\rangle \langle \mathrm{S}\|m\|\mathrm{P}\rangle \left\langle 1\bar{M}_LSM_S\middle|JM_J\right\rangle \\
&= \langle 1(-q)1q|00\rangle \langle \mathrm{S}\|m\|\mathrm{P}\rangle \langle 1(-q)SM_S|JM_J\rangle \\
&= -\frac{(-1)^q}{\sqrt{3}} \langle \mathrm{S}\|m\|\mathrm{P}\rangle \langle 1(-q)SM_S|JM_J\rangle
\end{aligned} \tag{S1}$$

where $\langle JMJ'M'|J''M''\rangle$ is a vector coupling (Clebsch–Gordan) coefficient and $\langle \mathrm{X}\|Y\|\mathrm{Z}\rangle$ is a reduced matrix element. Atoms are isotropically symmetric, so we can treat $\vec{B}$ along the z axis without integration over all directions. Thus, the equation from the main manuscript becomes

$$\begin{aligned}
\frac{\langle \Delta\epsilon_{\mathrm{MLD}}\rangle}{E} &= \int \gamma \sum_{\substack{a\in A\\ j\in J}} N_a \langle a|\vec{m}|j\rangle \cdot \left(\frac{3\hat{z}_{\mathrm{lab}}\hat{z}_{\mathrm{lab}}^{\mathsf{T}} - 1}{2}\right) \cdot \langle j|\vec{m}|a\rangle f(E) \frac{d\hat{z}_{\mathrm{lab}}}{4\pi} \\
&\rightarrow \gamma \sum_{M_SM_J} N_{M_S} \langle 00SM_S|\vec{m}|1SJM_J\rangle \cdot \begin{pmatrix} -\frac{1}{2} & 0 & 0 \\ 0 & -\frac{1}{2} & 0 \\ 0 & 0 & 1 \end{pmatrix} \cdot \langle 1SJM_J|\vec{m}|00SM_S\rangle f(E)
\end{aligned} \tag{S2}$$

and we can evaluate $N_{M_S} = e^{-\mu_B gBM_S/k_BT} / \sum_{M_S'} e^{-\mu_B gBM_S'/k_BT}$. Combining Eqs. (S1) and (S2), we get the following expressions for the MLD intensity as a function of field and temperature:

$$\begin{aligned}
S = 1:& \qquad \frac{\langle \Delta\epsilon_{\mathrm{MLD}}\rangle}{E} \propto \sum_{M=-S}^{S} \left(3M^2 - 2\right) e^{Mx} \Big/ \sum_{M=-S}^{S} e^{Mx} \\
S = \frac{3}{2}:& \qquad \frac{\langle \Delta\epsilon_{\mathrm{MLD}}\rangle}{E} \propto \sum_{M=-S}^{S} \left(\frac{4M^2 - 5}{4}\right) e^{Mx} \Big/ \sum_{M=-S}^{S} e^{Mx} \\
S = 2:& \qquad \frac{\langle \Delta\epsilon_{\mathrm{MLD}}\rangle}{E} \propto \sum_{M=-S}^{S} \left(\frac{M^2 - 2}{2}\right) e^{Mx} \Big/ \sum_{M=-S}^{S} e^{Mx} \\
&\qquad \vdots \\
\text{any } S \geq 1:& \qquad \frac{\langle \Delta\epsilon_{\mathrm{MLD}}\rangle}{E} \propto \sum_{M=-S}^{S} \left(\frac{3M^2 - S(S+1)}{S(2S-1)}\right) e^{Mx} \Big/ \sum_{M=-S}^{S} e^{Mx}
\end{aligned} \tag{S3}$$

where the expressions after the proportionality sign ($\propto$) have been normalized to asymptotically approach 1 as $x \rightarrow \infty$. We have abbreviated $x = g\mu_B B/k_BT$.

The proportionality constant itself will be a function of $\langle \mathrm{S}\|m\|\mathrm{P}\rangle$ and S and J. An expression for the constant is not of particular use for us for this abstract atomic ${}^{2S+1}\mathrm{S} \rightarrow {}^{2S+1}\mathrm{P}_J$ transition; instead, it is useful to see how it relates to the MLD Faraday $\mathcal{G}_0$ parameter. Let's call the proportionality constant itself

K. To find the relationship between K and $\mathcal{G}_0$, we take a Taylor expansion of Eq. (S3) to second order in B:

$$\frac{\langle \Delta\epsilon_{\mathrm{MLD}} \rangle}{E} \approx \frac{1}{30} K(S+1)(2S+3) \frac{g^2 \mu_B^2 B^2}{k_B^2 T^2} + O(B^4) \tag{S4}$$

and we equate this with the $\mathcal{G}_0$ portion of the weak-field expression for MLD intensity, $\gamma \mu_B^2 B^2 \mathcal{G}_0 / k_B^2 T^2$. Solving for K yields the MLD saturation expression in the main text.

S2.2 Room Temperature Data Collection and Processing

The sample of [**1**]$[PF_6]_3$ was a ~ 0.1 M solution in acetonitrile. The samples of [**1**]$[BPh_4]_2$ for room temperature measurements were ~ 0.1 mM solutions in acetonitrile or THF; the complex reacts deleteriously with methanol. The samples of $[\mathbf{1}]^0$ were ~ 0.03 mM solutions in THF and toluene, respectively. A 0.11 mM solution of K(mtpy) in THF was used.

Room temperature spectra were acquired at a series of field strengths (e.g. 0, ± 1, ± 1.5 T). These experimental conditions are in the linear limit ($\mu_B B / k_B T \ll 1$), giving MCD intensity strictly proportional to the applied field. Under these linear conditions, the field-independent and field-dependent components of the measured ellipticity data were separated using a Moore–Penrose pseudoinverse. For example, collection of five spectra over the 300–800 nm region at $B = 0, \pm 1, \pm 1.5$ T field strengths allows extraction of the baseline (field-independent $I^{(0)}$) and MCD (field-dependent $I^{(1)}$) components through

$$\begin{pmatrix} 1 & 1.5 \\ 1 & 1 \\ 1 & 0 \\ 1 & -1 \\ 1 & -1.5 \end{pmatrix}^{\ominus} \begin{pmatrix} I_{300\text{ nm}}^{+1.5\text{ T}} & I_{301\text{ nm}}^{+1.5\text{ T}} & I_{302\text{ nm}}^{+1.5\text{ T}} & \cdots & I_{800\text{ nm}}^{+1.5\text{ T}} \\ I_{300\text{ nm}}^{+1.0\text{ T}} & I_{301\text{ nm}}^{+1.0\text{ T}} & I_{302\text{ nm}}^{+1.0\text{ T}} & \cdots & I_{800\text{ nm}}^{+1.0\text{ T}} \\ I_{300\text{ nm}}^{0.0\text{ T}} & I_{301\text{ nm}}^{0.0\text{ T}} & I_{302\text{ nm}}^{0.0\text{ T}} & \cdots & I_{800\text{ nm}}^{0.0\text{ T}} \\ I_{300\text{ nm}}^{-1.0\text{ T}} & I_{301\text{ nm}}^{-1.0\text{ T}} & I_{302\text{ nm}}^{-1.0\text{ T}} & \cdots & I_{800\text{ nm}}^{-1.0\text{ T}} \\ I_{300\text{ nm}}^{-1.5\text{ T}} & I_{301\text{ nm}}^{-1.5\text{ T}} & I_{302\text{ nm}}^{-1.5\text{ T}} & \cdots & I_{800\text{ nm}}^{-1.5\text{ T}} \end{pmatrix} = \begin{pmatrix} I_{300\text{ nm}}^{(0)} & I_{301\text{ nm}}^{(0)} & I_{302\text{ nm}}^{(0)} & \cdots & I_{800\text{ nm}}^{(0)} \\ I_{300\text{ nm}}^{(1)} & I_{301\text{ nm}}^{(1)} & I_{302\text{ nm}}^{(1)} & \cdots & I_{800\text{ nm}}^{(1)} \end{pmatrix},$$

where "$\ominus$" indicates the pseudoinverse. The first column of ones in the leftmost matrix indicates that the baseline is constant among the data sets, and the second column of the leftmost matrix contains the strengths of the applied fields. The output gives the field-independent ellipticity in its first row (i.e. the baseline and any natural CD), and the field-dependent ellipticity in its second row (i.e. MCD expressed in millidegrees per tesla). The field-dependent ellipticity $[\theta]$ was converted to $\Delta\epsilon$ (M^{-1} cm^{-1} T^{-1}) MCD intensity by $\Delta\epsilon = [\theta\,(\mathrm{mdeg})]/(32982cl)$ using concentration c (M) and path length l (cm).

S2.3 Cryogenic Data Collection and Processing

MCD and MLD spectra were collected with the applied field parallel and perpendicular, respectively, to the direction of propagation of light at a series of fields and temperatures. The temperatures used for MCD were 1.611(7), 2.39(4), 3.26(3), 5.17(4), 7.88(2), and 11.97(2) K. The temperatures used for MLD were 1.618(10), 2.47(6), 3.02(8), 5.01(4), 8.05(3), 12.01(2), 20.00(1), and 35.2(1) K. The collection of spectra at both positive and negative field allowed for more confident extraction of MLD (even in field) from MCD bleedthrough (odd in field). All temperature/field combinations used are shown in Fig. S5.

The saturation behavior of the MCD and MLD features were found to follow the saturation curves shown in the main document for systems lacking appreciable ZFS. The MCD and MLD curves projected to the saturation limit were then extracted from the experimental data using a similar pseudoinverse technique to that described in Section S2.2. Instead of a two-column matrix with a column of ones (for the baseline) and a column of field strengths, we instead used a two-column matrix with a column of ones (for the baseline) and a column of the predicted saturation intensity at each temperature/field; for example in extracting MCD in the saturation limit we used

$$\begin{pmatrix} 1 & \frac{2S+1}{2S} \coth\left(\frac{2S+1}{2} \frac{g\mu_B B_1}{k_B T_1}\right) - \frac{1}{2S} \coth\left(\frac{1}{2} \frac{g\mu_B B_1}{k_B T_1}\right) \\ 1 & \frac{2S+1}{2S} \coth\left(\frac{2S+1}{2} \frac{g\mu_B B_2}{k_B T_2}\right) - \frac{1}{2S} \coth\left(\frac{1}{2} \frac{g\mu_B B_2}{k_B T_2}\right) \\ 1 & \frac{2S+1}{2S} \coth\left(\frac{2S+1}{2} \frac{g\mu_B B_3}{k_B T_3}\right) - \frac{1}{2S} \coth\left(\frac{1}{2} \frac{g\mu_B B_3}{k_B T_3}\right) \\ \vdots & \vdots \end{pmatrix}^{\ominus} \tag{S5}$$

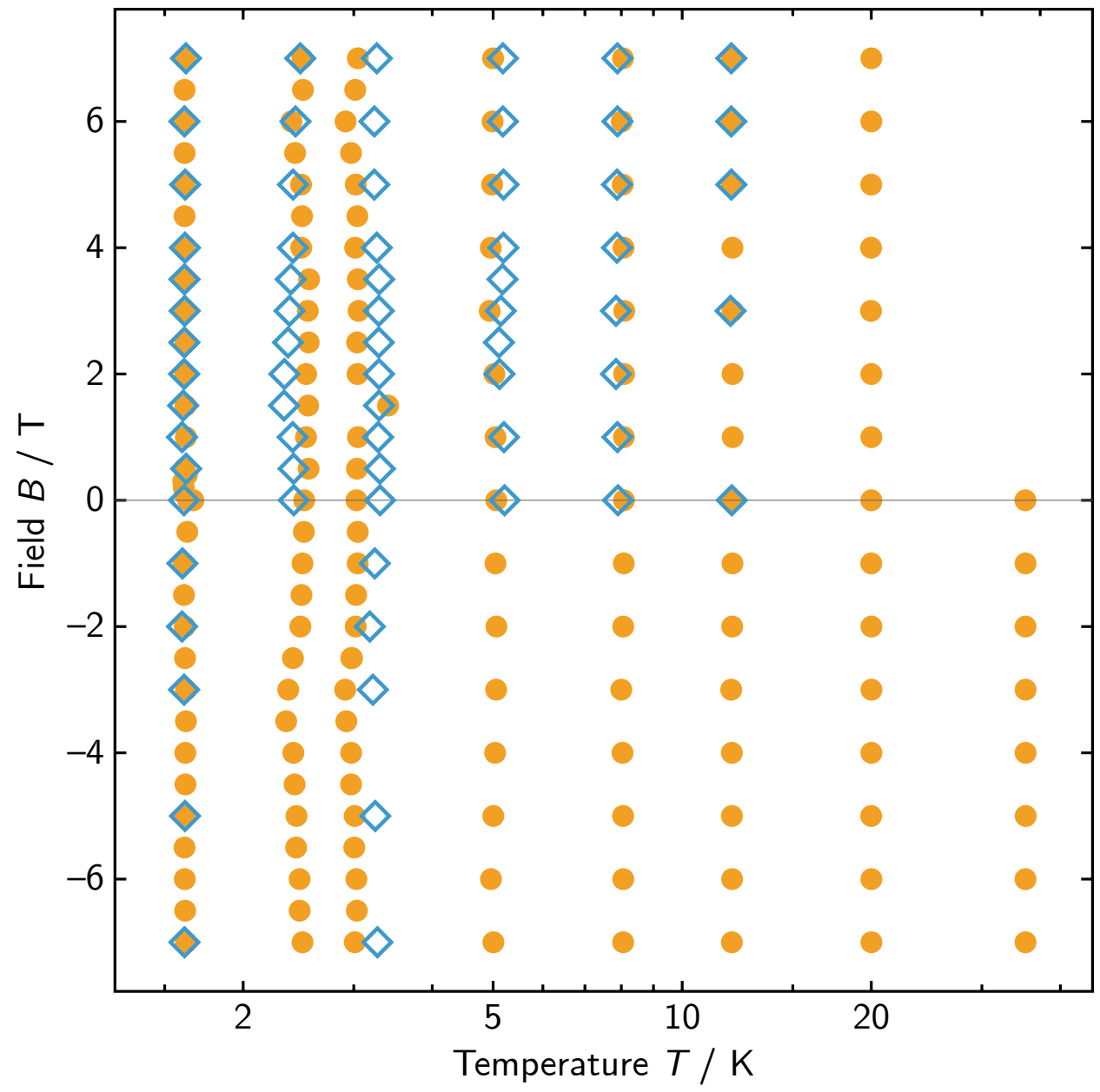

Figure S5: A grid showing all temperature/field combinations at which MCD (blue diamonds) and MLD (orange circles) spectra were acquired.

where (T_1, B_1) is the temperature/field pair for the first spectrum, (T_2, B_2) is the pair for the second, etc. Left multiplying this matrix pseudoinverse against a matrix of our MCD spectra provided two spectra: the field-independent baseline (which was near-zero due to our earlier baseline subtraction efforts) and the MCD spectra calculated at 100% magnetic saturation. The same technique was used to extract the MLD spectrum in the saturation limit, but the MLD saturation equation, $\sum_{M=-S}^{S} \left(\frac{3M^2-S(S+1)}{S(2S-1)}\right) e^{Mx} \Big/ \sum_{M=-S}^{S} e^{Mx}$ $(x = g\mu_B B/k_B T)$, was used instead of the MCD saturation equation.

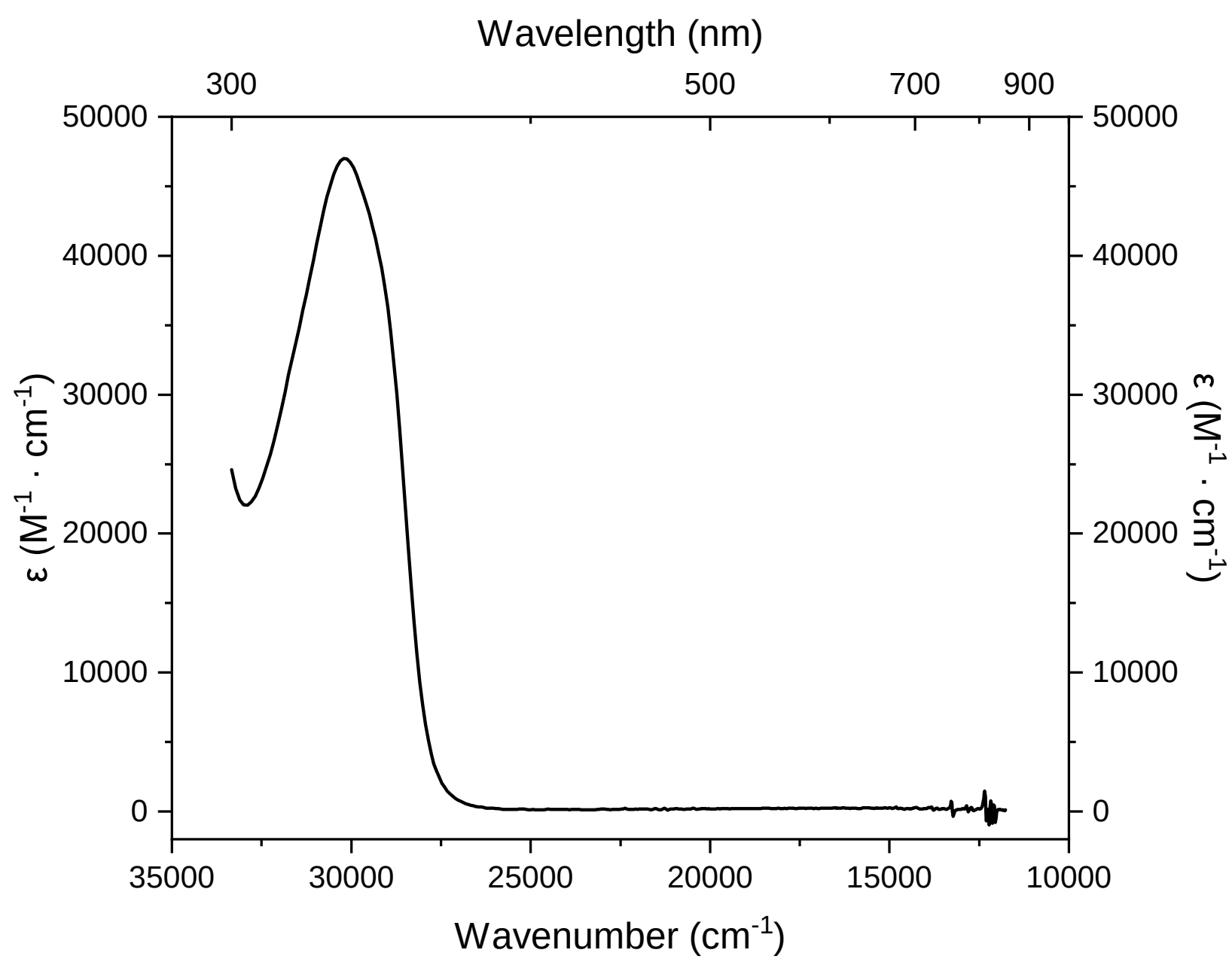

Figure S6: UV-vis absorbance spectrum of [**1**][PF_6]$_3$·MeCN in MeCN at ambient temperature.

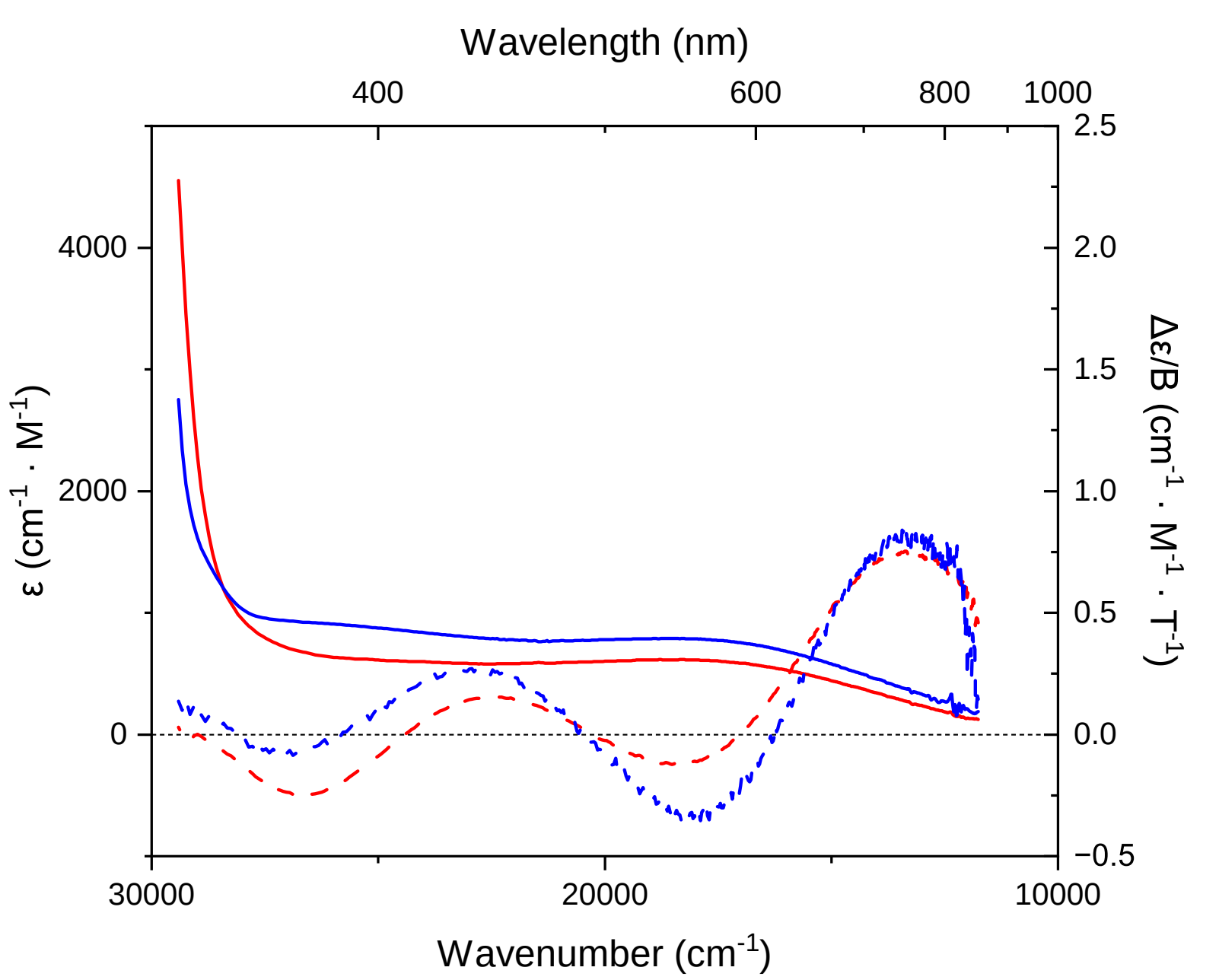

Figure S7: UV-vis Absorbance (solid) and MCD (dashed) spectra of [**1**][BPh_4]$_2$·2THF·Et_2O in THF (red) and acetonitrile (blue).

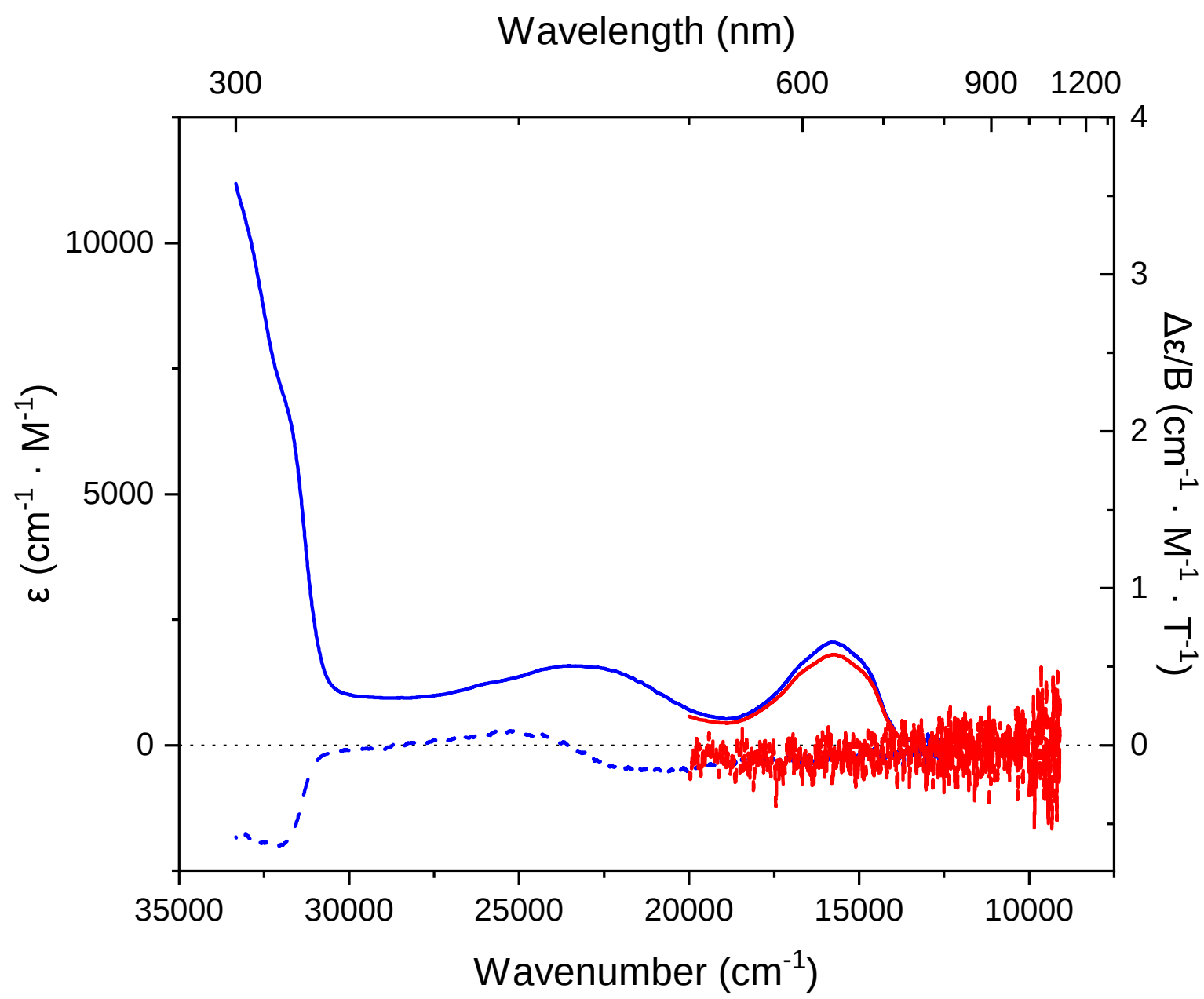

Figure S8: Absorbance (solid) and MCD (dashed) spectra of K(mtpy) in THF at ambient temperature. UV-vis spectra are in blue; visible-NIR spectra are in red.

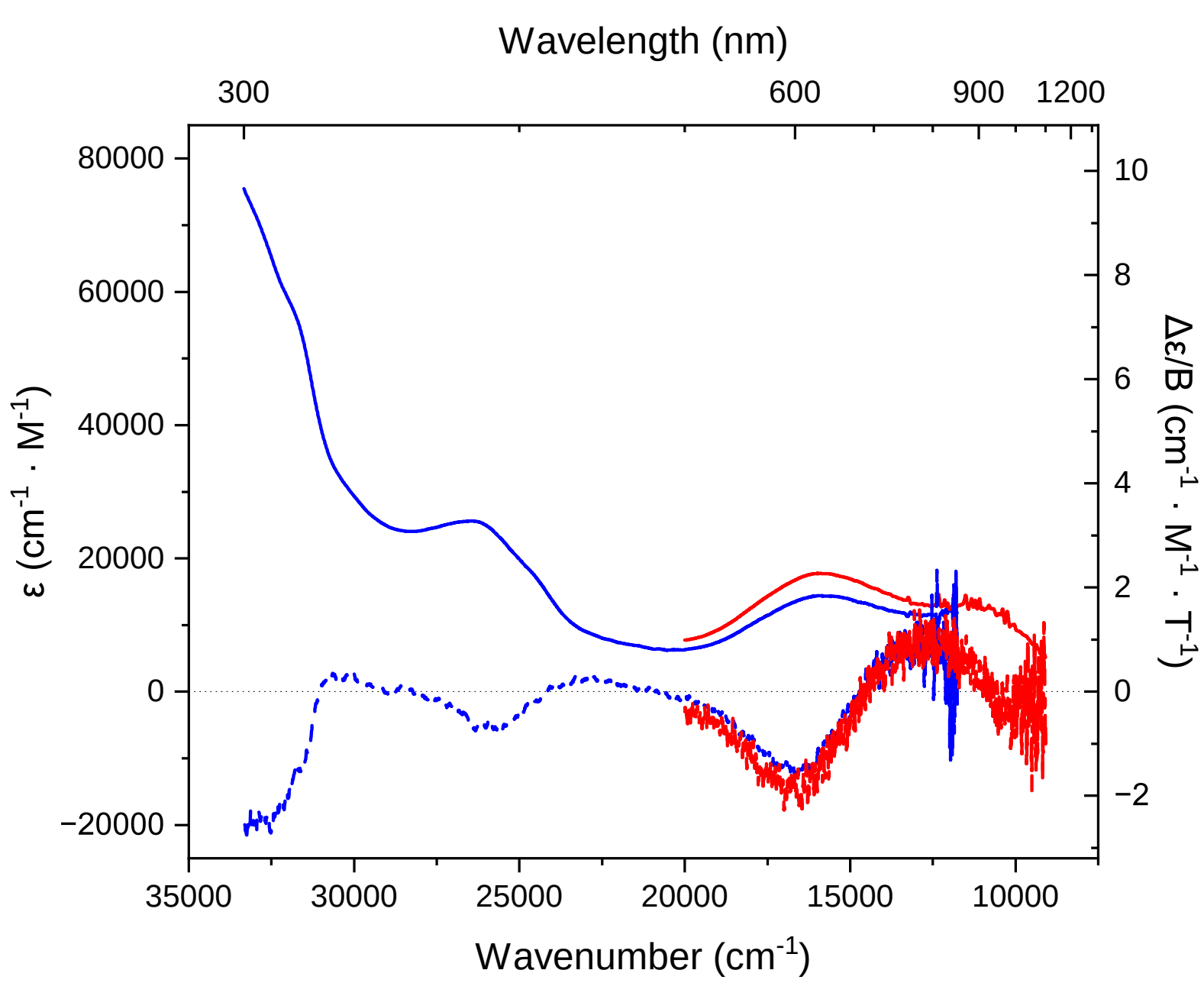

Figure S9: Absorbance (solid) and MCD (dashed) spectra of $[\mathbf{1}]^0$·2PhMe in toluene at ambient temperature. UV-vis spectra are in blue; visible-NIR spectra are in red.

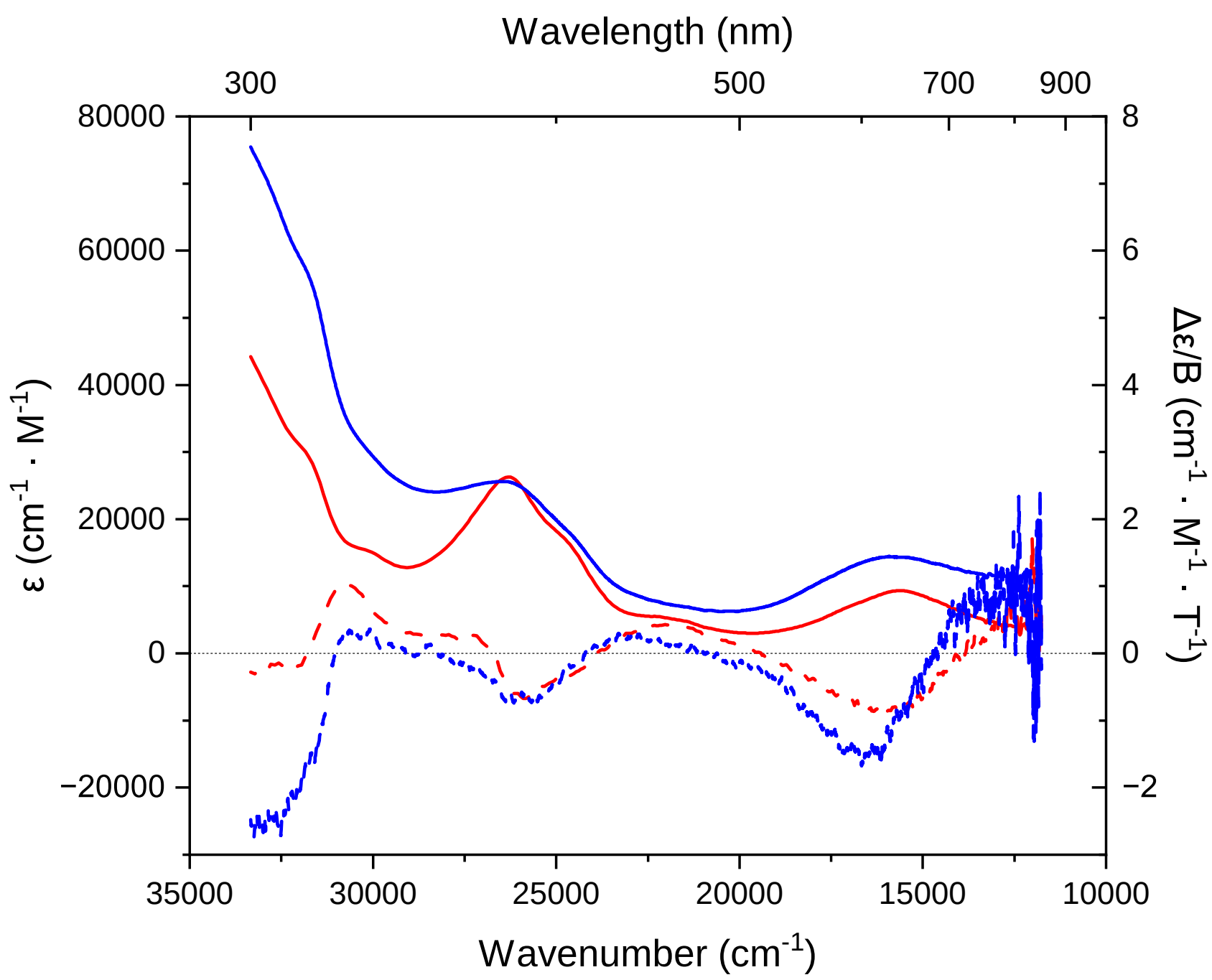

Figure S10: UV-vis absorbance (solid lines) and MCD (dashed lines) of $[\mathbf{1}]^0{\cdot}2PhMe$ in toluene (blue) and THF (red).

S3 Electron Paramagnetic Resonance

S3.1 Pulsed EPR Experiments

Dication Electron Spin Echo detected (FS-ESE) EPR experiments were performed on a Bruker ElexSys E680 CW/FT X-band spectrometer. All experiments were run at 5 K, maintained with an Oxford ITC503 temperature controller and an Oxford CF935 dynamic continuous-flow cryostat. The pulse sequence (Hahn-Echo) was $\pi/2 - \tau - \pi - \tau -$ echo, sweeping the magnetic field. The X-Band FS-ESE spectrum was collected with a Bruker ER4118-MD5 resonator with a 1 kW TWT amplifier. The $\pi/2$ and π pulses were rectangular with a length of 8/16 ns and the interpulse delay τ was set to 300 ns. Data was collected for 1024 points with a sweep width of 10000 G centered at 5480 G. The shot repletion time (SRT) was 100 µs and 100 shots per point were recorded (SPP). A total of five scans was collected.

The Q-Band FS-ESE spectrum was collected with a Bridge12 Technologies QLP Q-band resonator with a 300 W TWT amplifier. The $\pi/2$ and π pulses were rectangular with a length of 48/96 ns and the interpulse delay τ was set to 300 ns. Data was collected for 2048 points with a sweep width of 7000 G centered at 11000 G. At Q-band the shot repletion time (SRT) was 100 µs and 20 shots per point were recorded (SPP). Again, a total of five scans was collected.

Neutral The X-Band FS-ESE spectrum for the neutral species was collected with a Bruker ER4118-MD5 resonator with a 1 kW TWT amplifier. The $\pi/2$ and π pulses were rectangular with a length of 8/16 ns and the interpulse delay τ was set to 300 ns. Data was collected for 1024 points with a sweep width of 10000 G centered at 5480 G. The utilized shot repletion time (SRT) was 3000 µs and 100 shots per point were recorded (SPP). A total of 138 scans were collected to distinguish the spectrum from the noise.

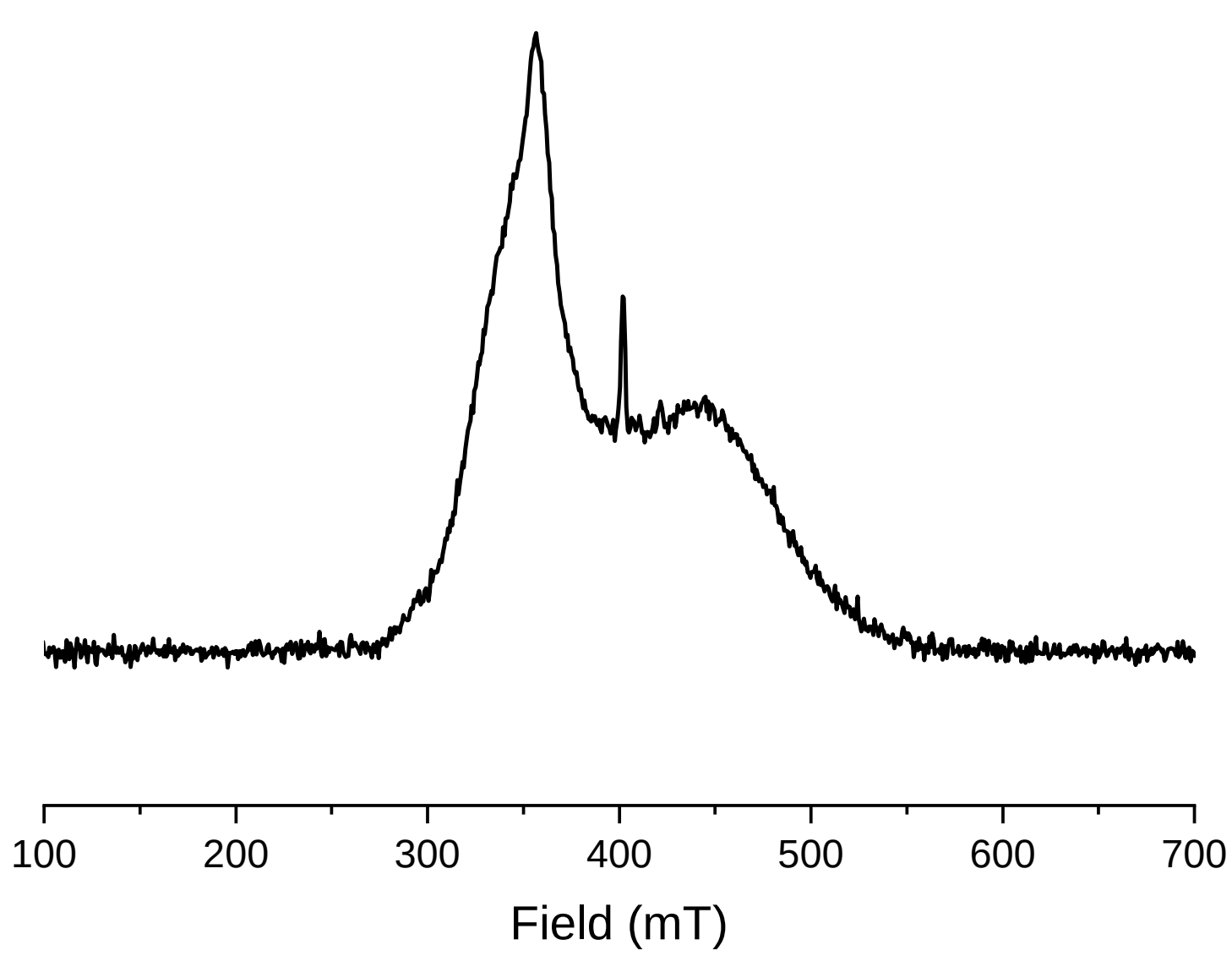

Figure S11: EDFS EPR spectrum of $[\mathbf{1}]^0 \cdot 2$PhMe in 2-methyltetrahydrofuran at 5K.

S3.2 Continuous Wave (CW) EPR Experiment on K(mtpy)

The room temperature CW-EPR experiment was performed with a X-Band Bruker EMX CW EPR spectrometer. Prior to the experiment, K(mtpy) was purified by recrystallization (hexane/THF layer diffusion at −30 °C). The sample (1.0 mM in 2-methyltetrahydrofuran) was contained in a 4 mm quartz EPR tube with a J Young tap. Data collection was initiated by conducting a power sweep, and a non-saturating power setting was selected to avoid any lineshape distortions. The spectrum was accumulated and averaged over eight scans. The spectrum were acquired using 25 dB attenuation (0.3162 mW power), a sweep width of 200 G, and a sweep time of 30 s, a modulation frequency of 100 kHz, a modulation amplitude of 1.0 G, a conversion time of 15.00 ms, a time constant of 1.28 ms, and 2 000 total data points.

The spectrum was simulated using the `garlic` function (isotropic/fast motion) in the EasySpin software.[10] The `esfit` subroutine was used to perform the fitting using a flat offset baseline and least squares autoscaling. Initial g value was estimated to be that of the free electron ($g_e = 2.0023$); the linewidth value was estimated by holding A values constant arbitrarily at 10 MHz and then floating the `g` and `lw` parameters against the spectrum as-is. A particle swarm algorithm was then used to float all values (`g`, `A`, and `lw`) simultaneously against the data. The best values obtained from the particle swarm were further refined using the Nelder–Mead simplex algorithm to obtain the final values listed in Table S1.

Table S1: EasySpin fit parameters for K(mtpy).

S	g	Nucs	n	A	lw
0.5	2.00546	‘N,N’	[1 2]	5.59701, 7.38996	0.30815

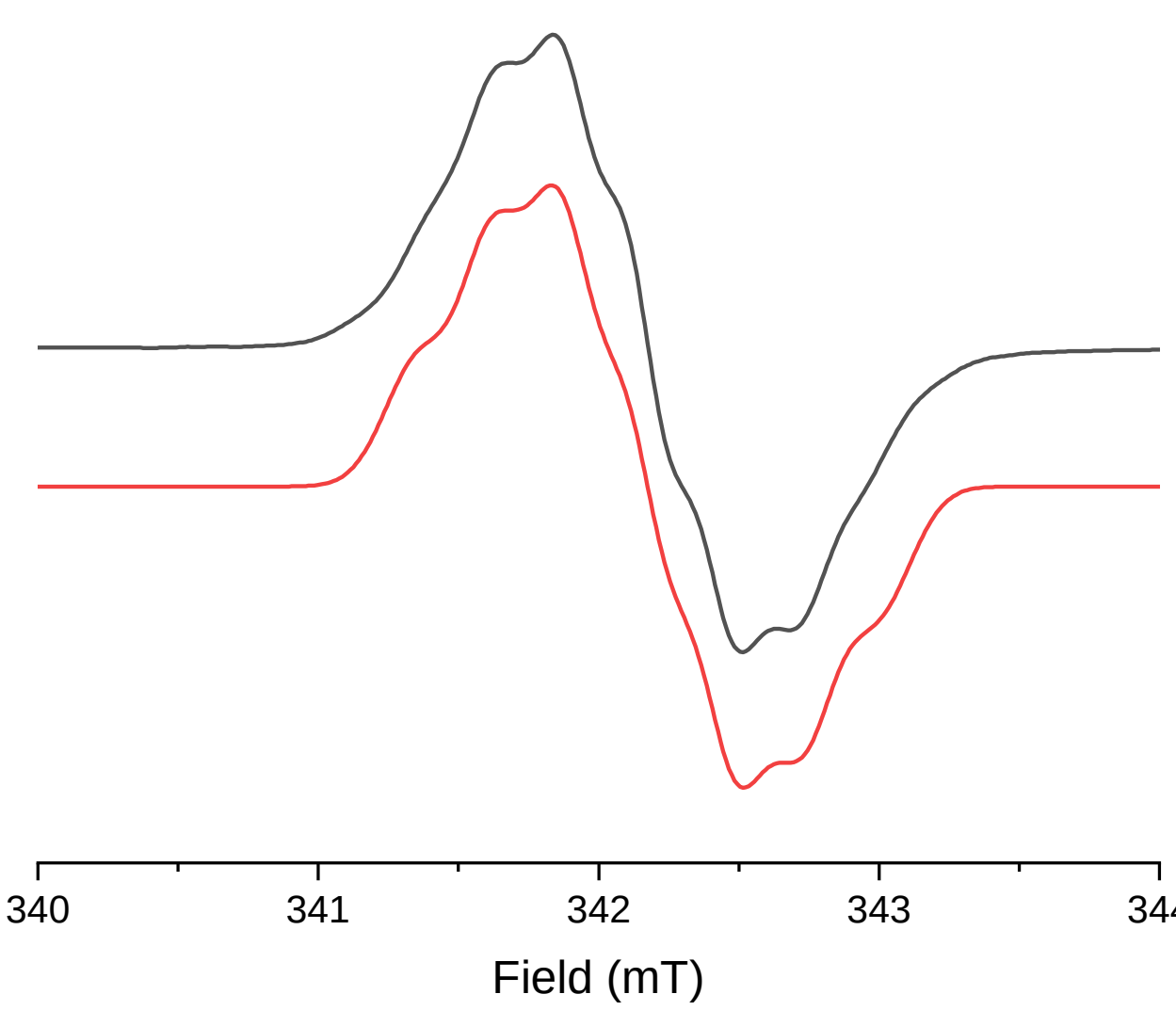

Figure S12: Experimental (black) and simulated (red) CW EPR spectrum of K(mtpy) at ambient temperature.

S4 X-ray Crystallography

The crystals were mounted in hydrocarbon oil on a nylon loop. Low-temperature (110–198 K) data were collected on a Bruker D8 Venture Duo diffractometer coupled to a Photon III CPAD ([**1**][PF_6]$_3$, [**1**][BPh_4]$_2$) or a Bruker D8 Quest Eco diffractometer coupled to a Apex II CCD ((mtpy)$_2$EuI_2·2MeCN and [**1**]^0·2(C_7H_8)) with Mo Kα radiaton (λ = 0.71073 Å) with ϕ- and ω-scans. Data were indexed with Apex II (Difference Vectors method), integrated with Bruker SAINT, and a multi-scan absorption correction was applied using SADABS. The space group was established using XPREP, and the structure was solved by intrinsic phasing methods using SHELXT-2018/2 and refined against F^2 on all data by full-matrix least squares with SHELXL-2019/2 using established methods. All non-hydrogen atoms were refined anisotropically. All hydrogen atoms were included in the model at geometrically calculated positions and refined using a riding model unless otherwise noted. The isotropic displacement parameters of all hydrogen atoms were fixed to 1.2 times the U_{eq} value of the atoms they are linked to (1.5 times for methyl groups).

S4.1 Refinement Details

Compound (mtpy)$_2$EuI_2 crystallized in the triclinic space group $P\bar{1}$ with one molecule and two MeCN molecules in the asymmetric unit. Two reflections that were either partially or completely obstructed by the beamstop were omitted from the final refinement.

Compound [**1**]^0 crystallized in the triclinic space group $P\bar{1}$ with the entire molecule and two residual toluene molecules in the asymmetric unit. The Eu atom resides on a general position. The phenyl rings of residual toluene molecules were constrained as ideal hexagons. One of the toluene molecules was disordered over two positions; the disorder was modeled using similarity restraints on the methyl carbon. The occupancy ratio between the two sides is 0.755(5):0.245(5). Disordered sites were restrained to have the same atomic displacement parameters. The unusually large U_3/U_1 ratios in the U_{ij} tensors of some carbon atoms might indicate that there may be other unmodeled disorders, but this shall not affect the accuracy of geometric parameter measurements. Six reflections that were either partially or completely obstructed by the beamstop were omitted from the final refinement. Small peaks resembling residual solvent molecules and otherwise unresolvable solvent molecule disorders were removed using PLATON SQUEEZE. [11]

Compound [**1**][BPh_4]$_2$ crystallized in the monoclinic space group $P2/n$ with a molecule of [**1**]$^{2+}$, two BPh_4^- anions, two molecules of THF, and a Et_2O molecule in the asymmetric unit. The Eu atom resides on a 2-fold rotational axis that bisects the cation. The hydrogen atoms of the methyl group residing on the 2-fold rotational axis was modeled using the AFIX 123 command. A methyl group on the mtpy ligand was found disordered over two positions. The disorder was modeled using similarity restraints on 1–2 and 1–3 distances. The occupancy ratio between the two sites is 0.531(14):0.469(14). One phenyl ring on the BPh_4^- anion was disordered over two positions. The phenyl group in each position was constrained as an ideal hexagon and restrained to have the same 1–2 and 1–3 distances. The occupancy ratio between the two sites is 0.658(10):0.342(10). The THF molecule was disordered over two positions. The disorder was modeled using similarity restraints on 1–2 and 1–3 distances. The occupancy ratio between the two sites is 0.625(5):0.375(5). The Et_2O molecule has a methyl group residing near a 2-fold axis and has a disordered ethyl group over two positions. The ethyl group was modeled using similarity restraints on 1–2 and 1–3 distances. The non-disordered part of the molecule was modeled using similar ADP and rigid-bond restraints and was restrained to have near-isotropic displacement parameters. The occupancy ratio of the two sites is 0.281(6):0.219(6). The hydrogen atoms of the Et_2O methyl groups were modeled using the AFIX 33 command. The unusually large U_3/U_1 ratios in the U_{ij} tensors of some carbon atoms suggest that there may be other unmodeled disorders, although this shall not affect the accuracy of geometric parameter measurements. For all modeled disorders, the occupied sites were restrained to have same atomic displacement parameters. Eight reflections that were either partially or completely obstructed by the beamstop were omitted from the final refinement. Small peaks resembling residual solvent molecules were removed using PLATON SQUEEZE. [11]

Compound [**1**][PF_6]$_3$ crystallized in the monoclinic space group $C2/c$ with three PF_6^- anions, and a MeCN molecule in the asymmetric unit. The Eu atom resides on a 2-fold rotational axis, which bisects the cation. The hydrogen atoms of the methyl group residing on the 2-fold rotational axis was modeled using the AFIX 123 command. One of the pyridine rings of mtpy disordered over two positions and was modeled with the help of similarity restraints on 1–2 and 1–3 distances. The displacement parameters of disordered

parts were restrained to be the same. The occupancy ratio between the two sites is 0.823(7):0.177(7). A substitutional disorder was found on one of the PF_6^- anions and the MeCN molecule. The MeCN molecule was refined using fixed 1–2 and 1–3 distance restraints. The carbon and nitrogen atoms in the MeCN molecule were also modeled using similar ADP and rigid-bond restraints as well as near-isotropic displacement parameters. The occupancy ratio between the PF_6^- anion and the MeCN molecule is constrained to be 0.5:0.5. For all modeled disorders, the two occupied sites were restrained to have same atomic displacement parameters. Small peaks resembling residual solvent molecules were removed using PLATON SQUEEZE.[11]

Table S2 summarizes crystallographic data for all europium compounds. Figures S13–S17 were made with ORTEP3[12] for each model. All hydrogen atoms and disorders are omitted for clarity. All ellipsoids are drawn at 30% probability. The following coloring scheme for atoms is used: boron, orange; carbon, white; nitrogen, blue; oxygen, red; fluorine, green; phosphorus, maroon; europium, gray; iodine, purple.

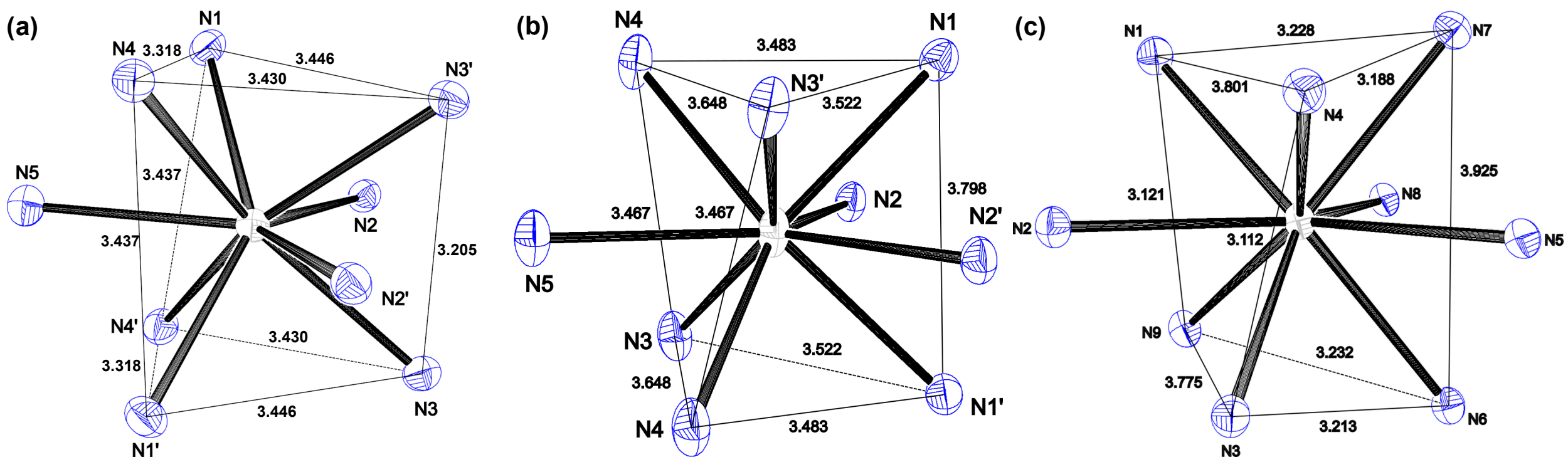

Figure S13: Coordination polyhedra measurements in Å for [**1**]$[PF_6]_3$·MeCN (a), [**1**]$[BPh_4]_2$·2THF·Et_2O (b), and $[\mathbf{1}]^0$·2(C_7H_8) (c). Symmetry-generated atoms are labeled with apostrophes.

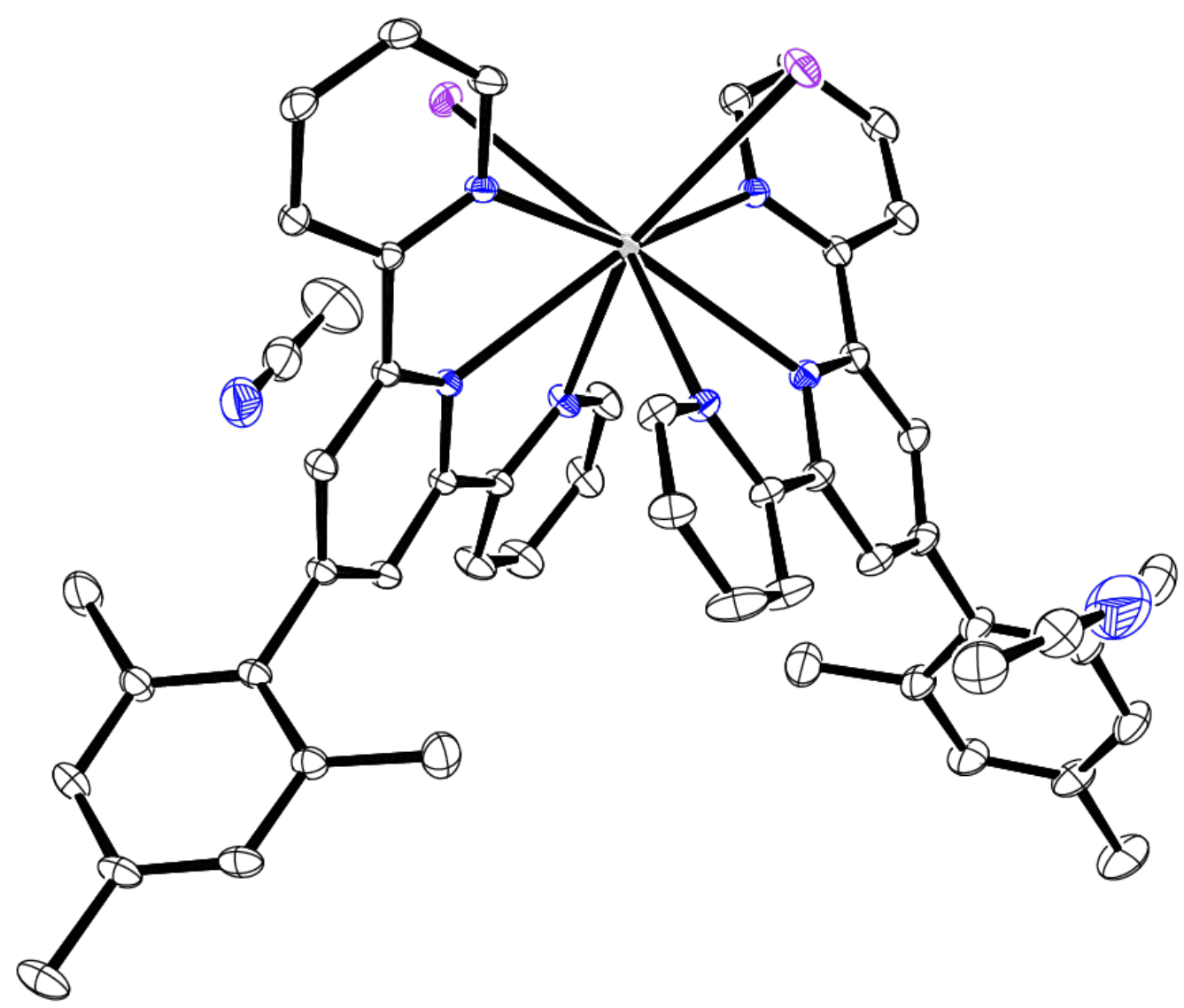

Figure S14: Thermal ellipsoid plot for the crystal structure of $(mtpy)_2EuI_2$·2MeCN.

Table S2: Summary of crystallographic data.

	$(mtpy)_2EuI_2·2MeCN$	**[1]**$^0·2(C_7H_8)$	**[1]**$[BPh_4]_2·2THF·Et_2O$	**[1]**$[PF_6]_3·MeCN$
CCDC	CSD 2550615	CSD 2550618	CSD 2550616	CSD 2550617
Empirical formula	$C_{26}H_{24}Eu_{0.50}IN_4$	$C_{21.50}H_{19.75}Eu_{0.25}N_{2.25}$	$C_{132}H_{129}B_2EuN_9O_3$	$C_{37}H_{33}Eu_{0.5}F_9N_5P_{1.5}$
Formula weight (g/mol)	595.37	347.63	2063.01	841.12
Color / Morphology	purple / block	purple / needle	purple / plate	colorless / block
Crystal size (mm^3)	0.369 × 0.270 × 0.142	0.604 × 0.140 × 0.047	0.628 × 0.409 × 0.230	0.238 × 0.108 × 0.108
Temperature (K)	180(2)	198(2)	119(2)	110(2)
Wavelength (Å)	0.71073	0.71073	0.71073	0.71073
Crystal system, Space group	Triclinic, $P\bar{1}$	Triclinic, $P\bar{1}$	Monoclinic, $P2/n$	Monoclinic, $C2/c$
Unit cell dimensions (Å, °)	$a = 11.6784(4)$, $\alpha = 72.728(2)$	$a = 11.3964(4)$, $\alpha = 69.6800(10)$	$a = 12.7324(13)$, $\alpha = 90$	$a = 23.1484(13)$, $\alpha = 90$
	$b = 11.8541(4)$, $\beta = 88.790(2)$	$b = 17.8939(6)$, $\beta = 75.1550(10)$	$b = 18.7249(17)$, $\beta = 91.479(4)$	$b = 22.9569(15)$, $\beta = 103.914(2)$
	$c = 19.7391(8)$, $\gamma = 73.257(2)$	$c = 19.7774(6)$, $\gamma = 88.5210(10)$	$c = 24.904(2)$, $\gamma = 90$	$c = 16.8016(11)$, $\gamma = 90$
Volume (Å^3)	2492.57(16)	3647.0(2)	5935.4(10)	8666.6(9)
Z	4	8	2	8
Density (calc., g/cm^3)	1.587	1.266	1.154	1.289
Absorption coefficient (mm^{-1})	2.539	0.911	0.582	0.861
$F(000)$	1170	1442	2162	3400
Theta range for data collection (°)	2.250 to 36.442	2.106 to 28.375	2.084 to 26.372	1.774 to 26.431
Index ranges	$-19 \leq h \leq 19$, $-19 \leq k \leq 19$, $-32 \leq l \leq 32$	$-15 \leq h \leq 15$, $-23 \leq k \leq 23$, $-26 \leq l \leq 26$	$-15 \leq h \leq 15$, $-23 \leq k \leq 23$, $-31 \leq l \leq 31$	$-28 \leq h \leq 28$, $-28 \leq k \leq 28$, $-21 \leq l \leq 21$
Reflections collected	263095	177739	183448	98249
Independent reflections, R_{int}	24261, 0.0668	18206, 0.0700	12128, 0.0693	8898, 0.0849
Completeness to $\theta = 25.242°$	99.9	99.9	99.8	99.9
Absorption correction	Multi-scan	Multi-scan	Multi-scan	Multi-scan
Refinement method	Full-matrix least squares on F^2	Full-matrix least squares on F^2	Full-matrix least squares on F^2	Full-matrix least squares on F^2
Data / Restraints / Parameters	24261 / 0 / 576	18206 / 17 / 863	12128 / 92 / 715	8898 / 55 / 542
Goodness-of-fit[a]	1.067	1.044	1.078	1.040
Final R indices[b] $[I > 2\sigma(I)]$	$R_1 = 0.0335$, $wR_2 = 0.0669$	$R_1 = 0.0316$, $wR_2 = 0.0741$	$R_1 = 0.0499$, $wR_2 = 0.1368$	$R_1 = 0.0340$, $wR_2 = 0.0849$
R indices[b] (all data)	$R_1 = 0.0434$, $wR_2 = 0.0704$	$R_1 = 0.0416$, $wR_2 = 0.0784$	$R_1 = 0.0584$, $wR_2 = 0.1484$	$R_1 = 0.0401$, $wR_2 = 0.0890$
Largest diff. peak and hole ($e·Å^{-3}$)	1.389 and −1.019	0.501 and −0.385	1.479 and −1.246	0.983 and −0.526

[a] Goodness-of-fit = $\sqrt{\frac{\sum[w(F_o^2-F_c^2)^2]}{(n-p)}}$ [b] $R_1 = \frac{\sum||F_o|-|F_c||}{\sum|F_o|}$; $wR_2 = \sqrt{\frac{\sum[w(F_o^2-F_c^2)^2]}{\sum[w(F_o^2)^2]}}$; $w = \frac{1}{\sigma^2(F_o^2)+(aP)^2+bP}$; $P = \frac{2F_c^2+\max(F_o^2,0)}{3}$

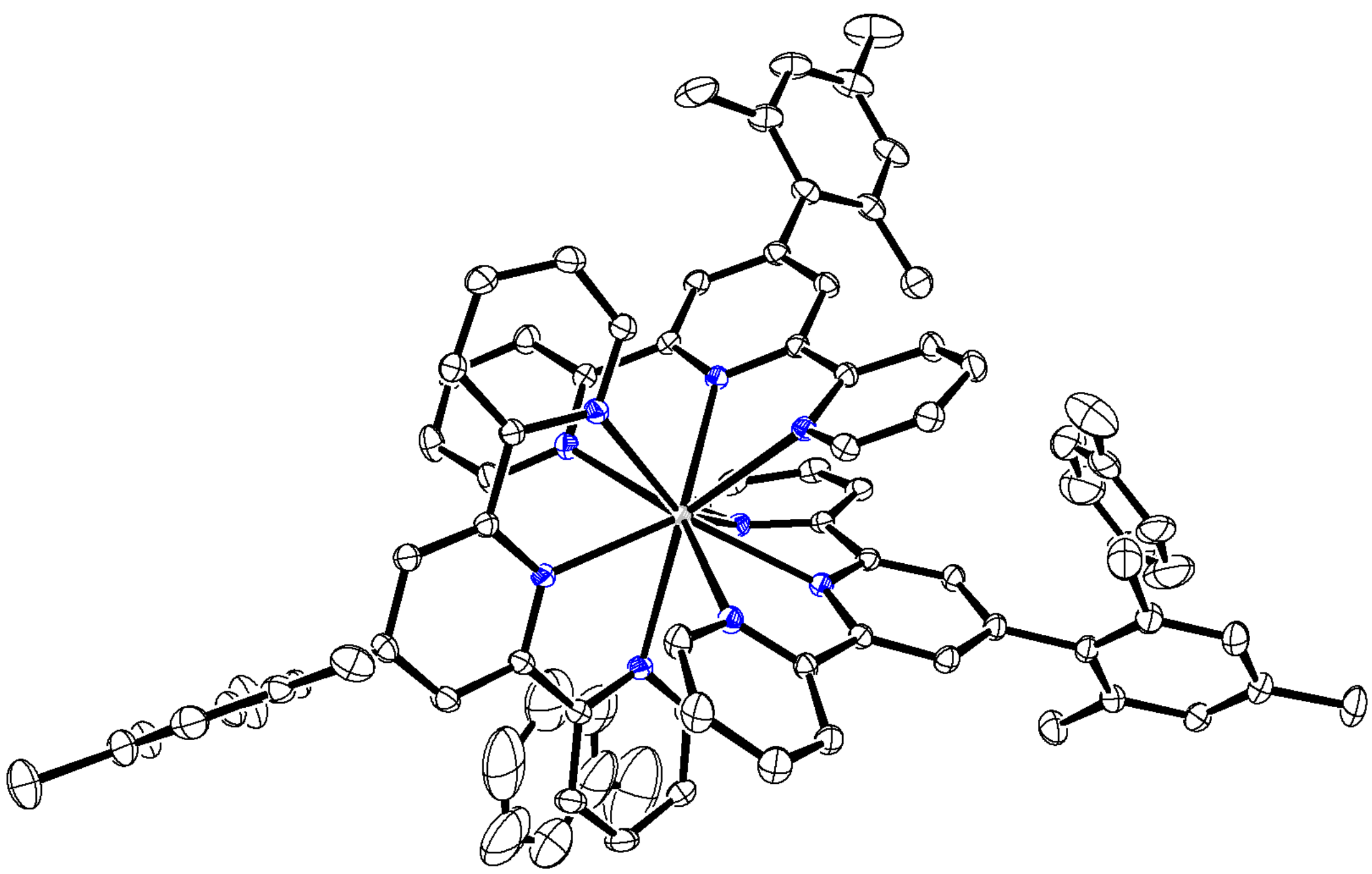

Figure S15: Thermal ellipsoid plot for the crystal structure of $[\mathbf{1}]^0{\cdot}2(C_7H_8)$.

S4.2 Symmetry Analysis

We can explore how appropriate it is to described the central EuN_9 unit of each $[\mathbf{1}]^{n+}$ system using D_{3h} symmetry by calculating the root mean square deviation of the structure from itself before and after application of each D_{3h} symmetry operation. We describe our approach to doing so below.

Approximate C_3 and C_2' axes were estimated from the coordinates of the EuN_9 atoms extracted from the refined crystal structures. To do so, all ten EuN_9 atoms were translated so that the europium center lied at the origin. Transformation matrices were computed for each of the following symmetry operations: C_3, C_3^2, $C_2'(1)$, $C_2'(2)$, and $C_2'(3)$. As an example, the 3×3 transformation matrix associated with the C_3 operation was calculated as

$$\Gamma(C_3) = \begin{pmatrix} x_{\mathrm{Eu}} & y_{\mathrm{Eu}} & z_{\mathrm{Eu}} \\ x_{\mathrm{N1}} & y_{\mathrm{N1}} & z_{\mathrm{N1}} \\ x_{\mathrm{N2}} & y_{\mathrm{N2}} & z_{\mathrm{N2}} \\ x_{\mathrm{N3}} & y_{\mathrm{N3}} & z_{\mathrm{N3}} \\ x_{\mathrm{N4}} & y_{\mathrm{N4}} & z_{\mathrm{N4}} \\ x_{\mathrm{N5}} & y_{\mathrm{N5}} & z_{\mathrm{N5}} \\ x_{\mathrm{N6}} & y_{\mathrm{N6}} & z_{\mathrm{N6}} \\ x_{\mathrm{N7}} & y_{\mathrm{N7}} & z_{\mathrm{N7}} \\ x_{\mathrm{N8}} & y_{\mathrm{N8}} & z_{\mathrm{N8}} \\ x_{\mathrm{N9}} & y_{\mathrm{N9}} & z_{\mathrm{N9}} \end{pmatrix}^{\ominus} \underbrace{\begin{pmatrix} 1&0&0&0&0&0&0&0&0&0 \\ 0&0&1&0&0&0&0&0&0&0 \\ 0&0&0&1&0&0&0&0&0&0 \\ 0&1&0&0&0&0&0&0&0&0 \\ 0&0&0&0&0&1&0&0&0&0 \\ 0&0&0&0&0&0&1&0&0&0 \\ 0&0&0&0&1&0&0&0&0&0 \\ 0&0&0&0&0&0&0&0&1&0 \\ 0&0&0&0&0&0&0&0&0&1 \\ 0&0&0&0&0&0&0&1&0&0 \end{pmatrix}}_{R(C_3)} \begin{pmatrix} x_{\mathrm{Eu}} & y_{\mathrm{Eu}} & z_{\mathrm{Eu}} \\ x_{\mathrm{N1}} & y_{\mathrm{N1}} & z_{\mathrm{N1}} \\ x_{\mathrm{N2}} & y_{\mathrm{N2}} & z_{\mathrm{N2}} \\ x_{\mathrm{N3}} & y_{\mathrm{N3}} & z_{\mathrm{N3}} \\ x_{\mathrm{N4}} & y_{\mathrm{N4}} & z_{\mathrm{N4}} \\ x_{\mathrm{N5}} & y_{\mathrm{N5}} & z_{\mathrm{N5}} \\ x_{\mathrm{N6}} & y_{\mathrm{N6}} & z_{\mathrm{N6}} \\ x_{\mathrm{N7}} & y_{\mathrm{N7}} & z_{\mathrm{N7}} \\ x_{\mathrm{N8}} & y_{\mathrm{N8}} & z_{\mathrm{N8}} \\ x_{\mathrm{N9}} & y_{\mathrm{N9}} & z_{\mathrm{N9}} \end{pmatrix} \quad \text{(S6)}$$

where '$\ominus$' indicates the Moore–Penrose pseudoinverse and $R(C_3)$ is a 10×10 matrix specifying how the atoms interconvert upon application of a C_3 rotation to the system. Although this $\Gamma(C_3)$ matrix is a 3×3 transformation matrix, it is not necessarily an orthogonal one (as any rotation must be). We can find the 'nearest' orthogonal matrix using singular value decomposition: if $\Gamma(C_3) = U\Sigma V^{\mathsf{T}}$ then the nearest

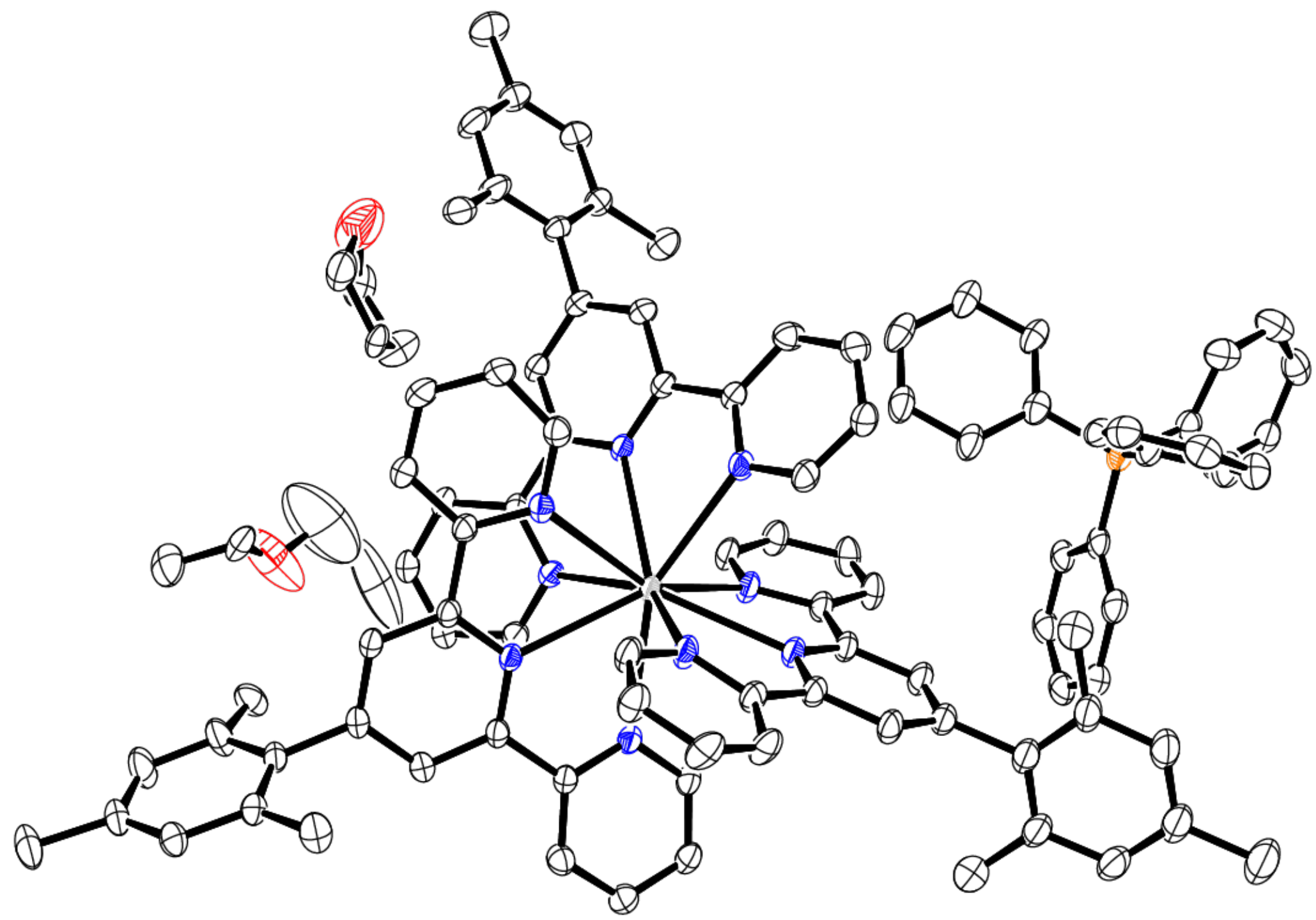

Figure S16: Thermal ellipsoid plot for the crystal structure of [**1**][BPh_4]$_2$·2THF·Et_2O.

orthogonal matrix is $\tilde{\Gamma}(C_3) = UV^{\mathsf{T}}$. This orthogonalized matrix will have two complex eigenvectors and one real eigenvector; the real eigenvector is what we have used as the 'best' C_3 axis of rotation for the given coordinates. Once we had obtained the 'best' axis for each of the proper rotations, these axes were assembled into a 5×3 matrix as follows,

$$M = \begin{pmatrix} x_{C_3} & y_{C_3} & z_{C_3} \\ x_{C_3^2} & y_{C_3^2} & z_{C_3^2} \\ x_{C_2'(1)} & y_{C_2'(1)} & z_{C_2'(1)} \\ x_{C_2'(2)} & y_{C_2'(2)} & z_{C_2'(2)} \\ x_{C_2'(3)} & y_{C_2'(3)} & z_{C_2'(3)} \end{pmatrix} \tag{S7}$$

and singular value decomposition was once again used find an orthogonal transformation. This was accomplished by $M = \bar{U}\bar{\Sigma}\bar{V}^{\mathsf{T}}$ (where macrons have been added to help distinguish from the earlier singular value decomposition), and the columns of $\bar{V}^{\mathsf{T}}$ are three orthonormalized vectors that (in some sense) are the closest one can get to symmetry axes based on the imperfect coordinates of each atom from the crystal structure. The axis closest to the earlier C_3 axis was used as the z axis and whichever of the other two axes lied best along a Eu–N_{eq} bond was used as the x axis. Once the system was rotated into the new coordinate system, we constructed matrices for each symmetry operation using the following generating set:

$$C_3 = \begin{pmatrix} -1/2 & \sqrt{3}/2 & 0 \\ -\sqrt{3}/2 & -1/2 & 0 \\ 0 & 0 & 1 \end{pmatrix}, \quad C_2'(1) = \begin{pmatrix} 1 & 0 & 0 \\ 0 & -1 & 0 \\ 0 & 0 & -1 \end{pmatrix}, \quad \sigma_h = \begin{pmatrix} 1 & 0 & 0 \\ 0 & 1 & 0 \\ 0 & 0 & -1 \end{pmatrix}. \tag{S8}$$

This work allowed us to quantify how 'closely' the input coordinates are described by symmetry operations of the D_{3h} point group. The ten extracted EuN_9 atoms were subjected to each of the symmetry operations generated by Eq. (S8), and for each symmetry operation the distance to the nearest original (unmoved) atom was calculated. The root mean square deviations for each symmetry operation are shown in Table S3.

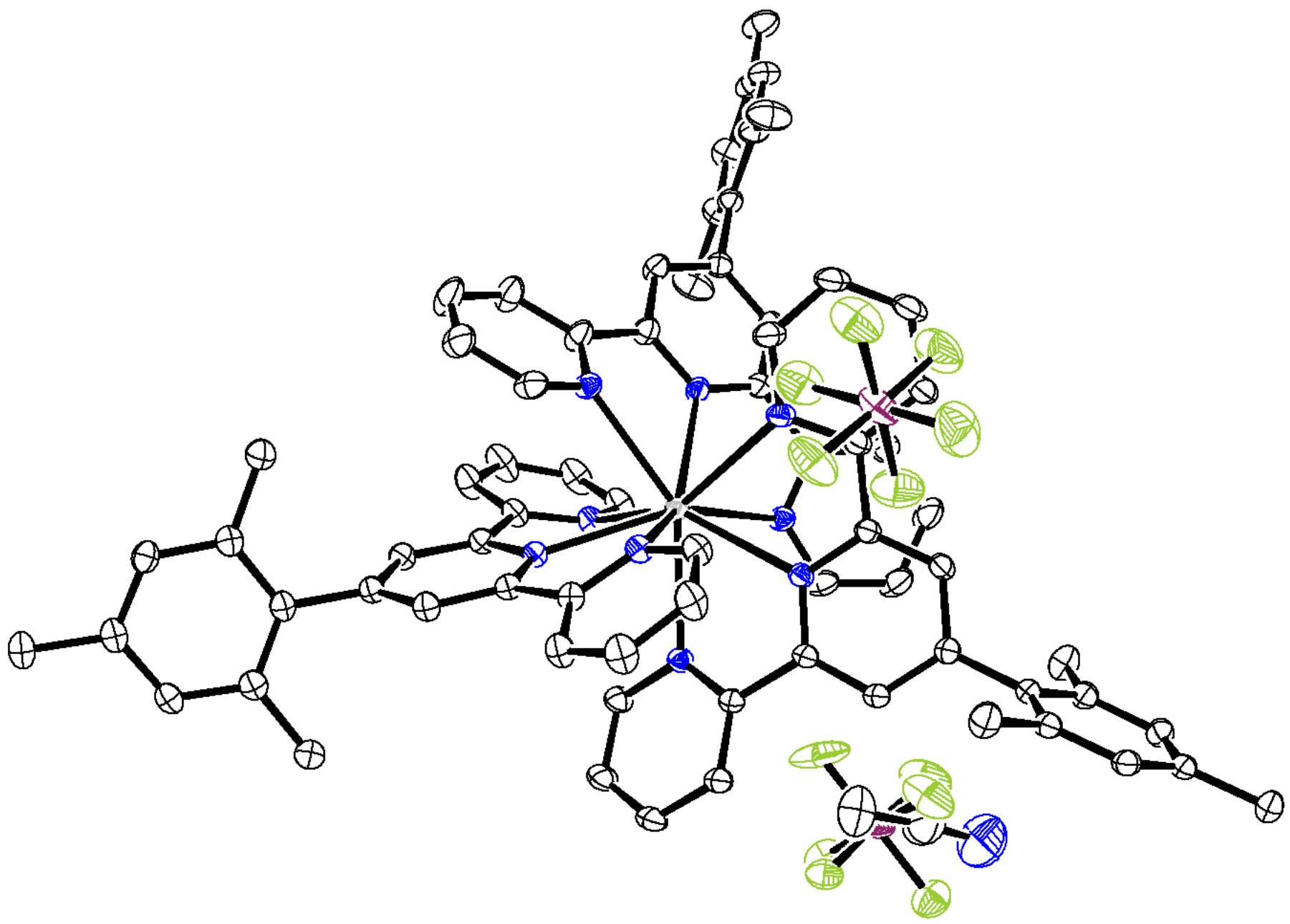

Figure S17: Thermal ellipsoid plot for the crystal structure of [**1**][PF_6]$_3$·MeCN.

Table S3: Root mean square deviation after each symmetry operation in the $D_{3\mathrm{h}}$ point group.

	E	$2C_3$	$3C_2'$	σ_h	$2S_3$	$3\sigma_v$
[**1**]$^{3+}$	0	0.140622	0.093760	0.339517	0.365735	0.356996
[**1**]$^{2+}$	0	0.284878	0.190416	0.599317	0.648996	0.632436
[**1**]^{0}	0	0.595072	0.415946	0.431345	0.718209	0.622731

Values are averaged within each class. The values indicate how 'close' (in Å) the system is to truly exhibiting a symmetry operation by telling us how far off (on average) the atoms are from being exactly interchanged by the symmetry operation. It can be seen that the X-ray structure of [**1**]$^{3+}$ adheres to the proper subgroup D_3 quite well, but also shows relatively close agreement for the improper σ_h, $2S_3$, and $3\sigma_v$ operations. As the oxidation state falls, the structures deviate more from $D_{3\mathrm{h}}$ (and even D_3).

S5 Electrochemistry

Electrochemical experiments were conducted using a CH Instrument 750F bipotentiostat under inert atmosphere. The electrochemical cell consists of a Pt disc working electrode, a Pt wire counter electrode, and a non-aqueous Ag/$AgNO_3$ reference electrode. A 0.1 M tetrabutylammonium hexafluorophosphate solution in MeCN was used as the supporting electrolyte solution. Under these circumstances, the $E_{1/2}$ of the ferrocene/ferrocenium couple, to which all measured potentials were referenced, was found at 0.0475 V versus the Ag/$AgNO_3$ redox couple.

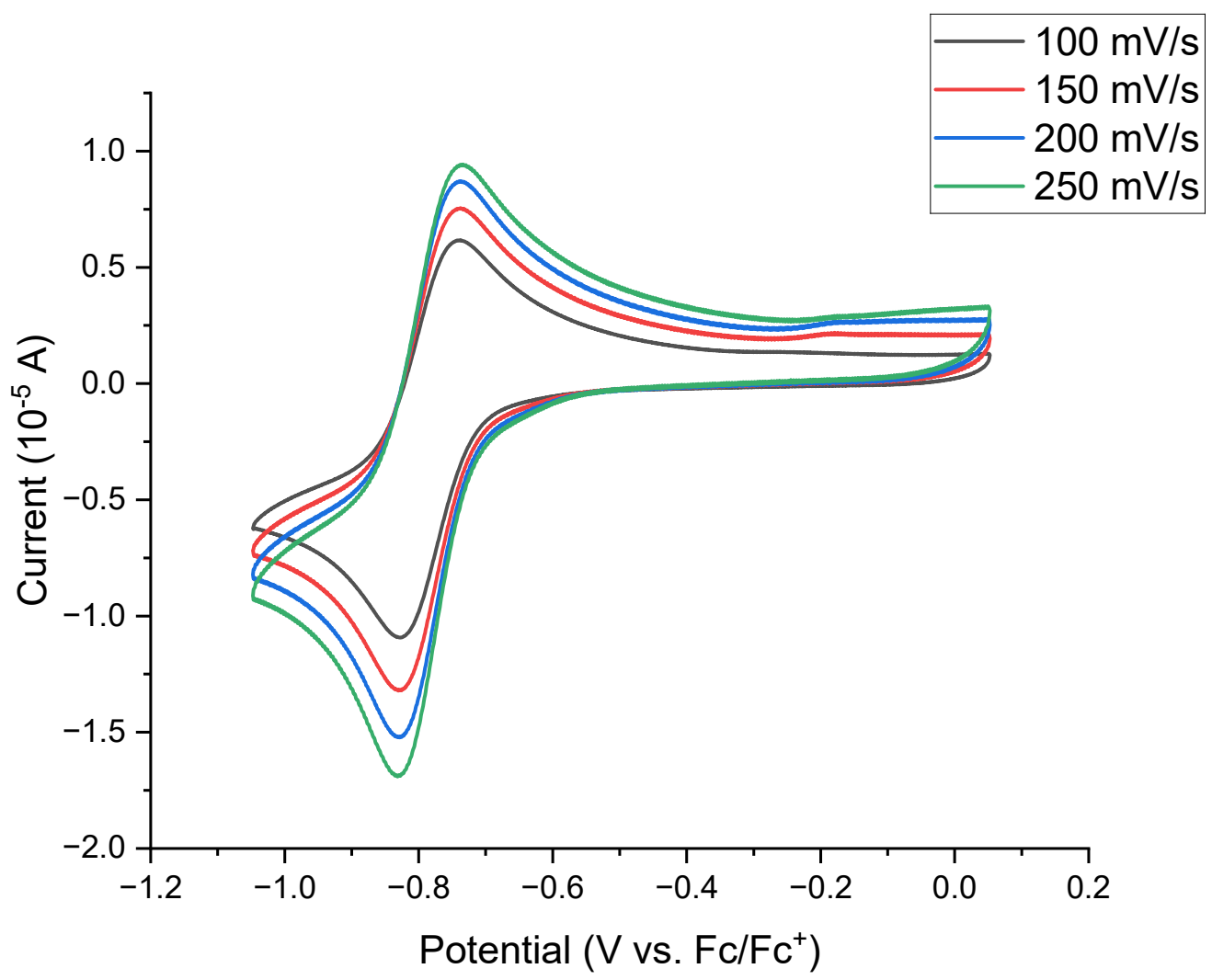

Figure S18: Cyclic voltammograms of [**1**][PF_6]$_3$·MeCN at various scan rates.

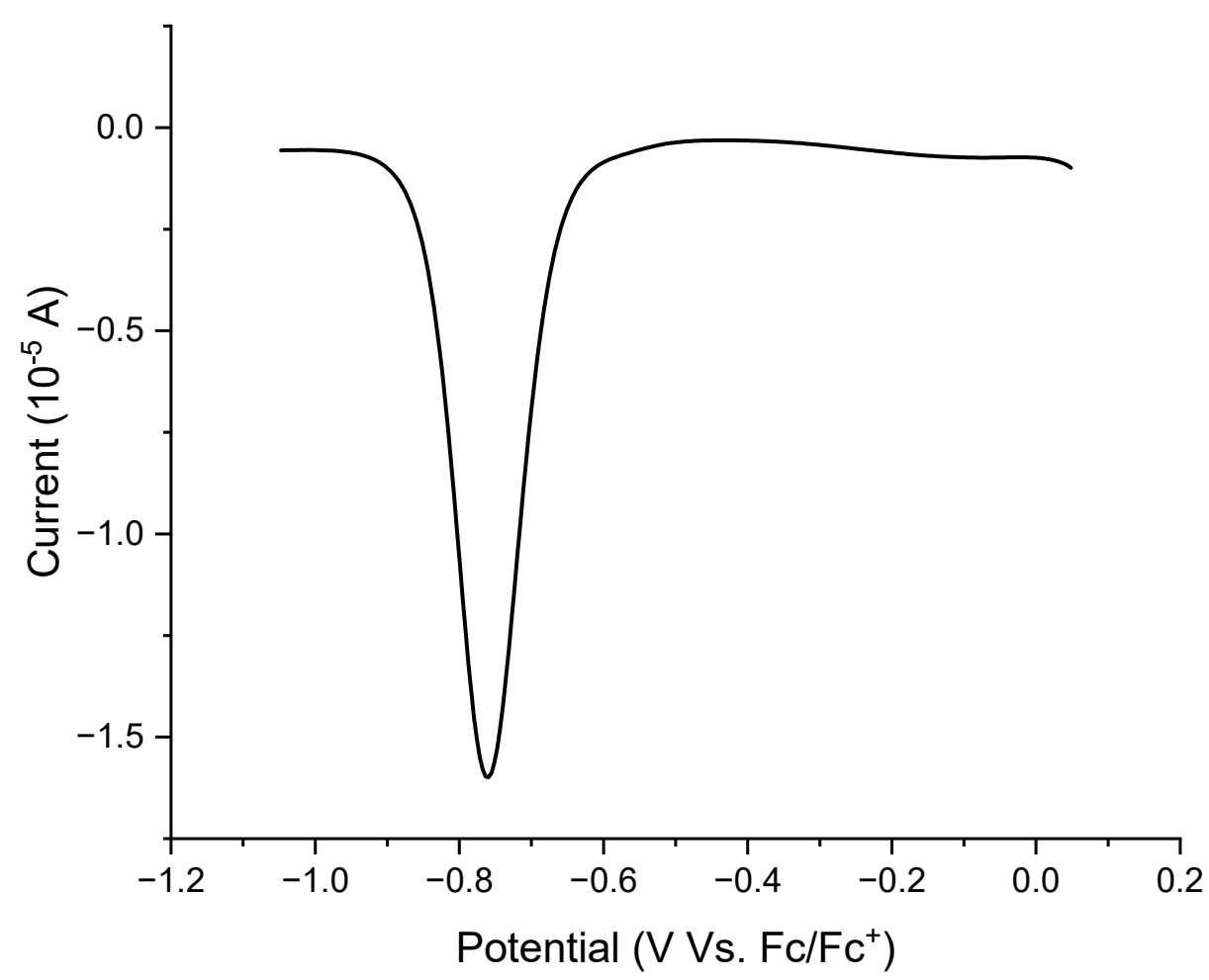

Figure S19: Differential pulse voltammogram of [**1**][PF_6]$_3$·MeCN.

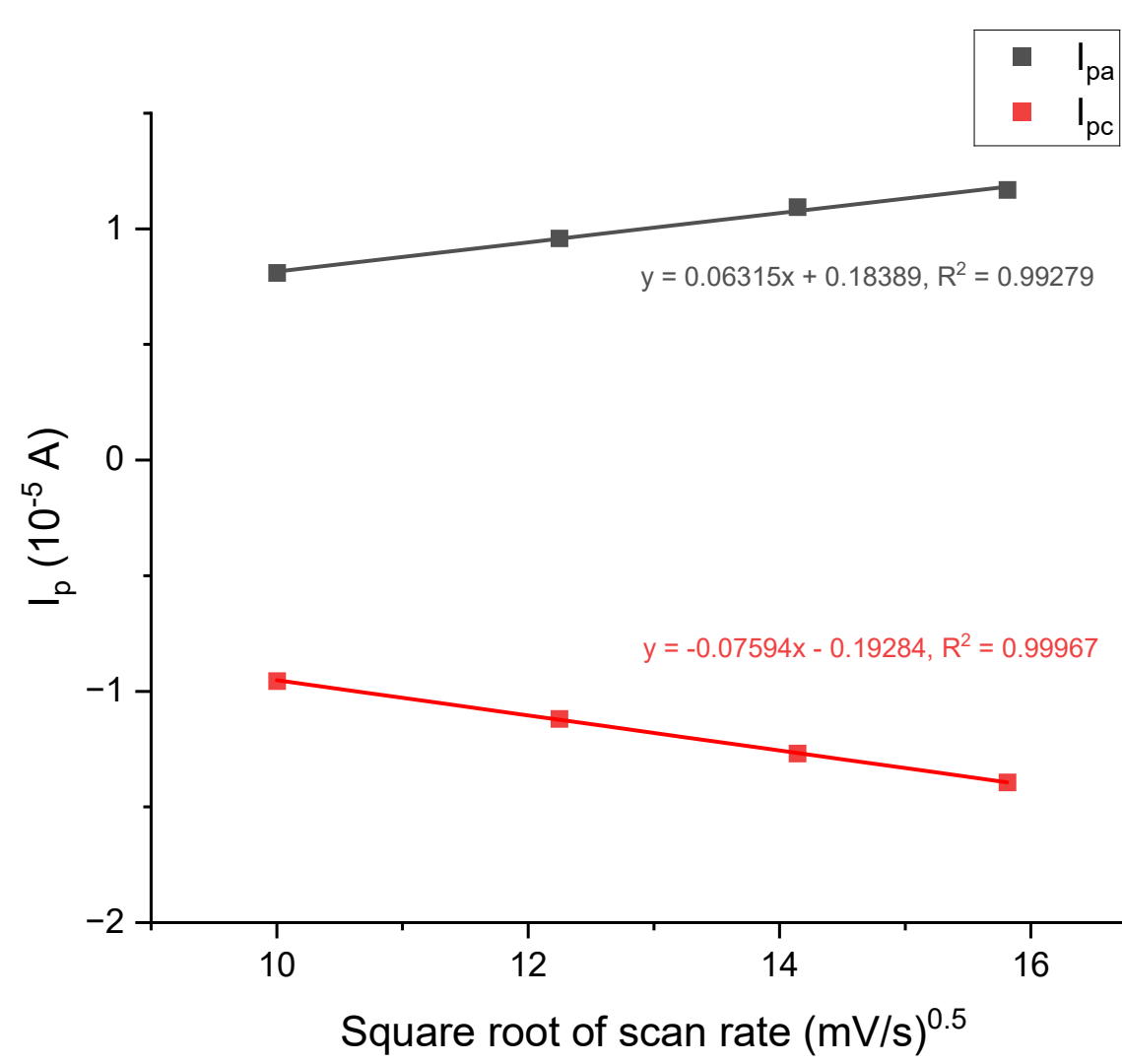

Figure S20: Anodic (I_{pa}) and cathodic (I_{pc}) peak currents measured for [**1**][PF_6]$_3$·MeCN at various scan rates and their linear fitting versus the square root of scan rates.

References

[1] Armarego, W. L. F.; Chai, C. L. L. *Purification of Laboratory Chemicals*, 6th ed.; Butterworth-Heinemann, 2009.

[2] Schwindt, M. A.; Lejon, T.; Hegedus, L. S. Improved Synthesis of (Aminocarbene)Chromium(0) Complexes with Use of C_8K-generated $Cr(CO)_5{}^{2-}$. Multivariant Optimization of an Organometallic Reaction. *Organometallics* **1990**, *9*, 2814–2819.

[3] Zhao, H.; Wijerathna, A. M. S. D.; Dong, Q.; Bai, Q.; Jiang, Z.; Yuan, J.; Wang, J.; Chen, M.; Zirnheld, M.; Li, R.; Liu, D.; Wang, P.; Zhang, Y.; Li, Y. Adjusting the Architecture of Heptagonal Metallo-Macrocycles by Embedding Metal Nodes into the Backbone. *Angew. Chem. Int. Ed.* **2024**, *63*, e202318029.

[4] Yoshihiro, O.; Toshiyuki, I. Characterizations of Divalent Lanthanoid Iodides in Tetrahydrofuran by UV-Vis, Fluorescence and ESR Spectroscopy. *Inorganica Chim. Acta* **1988**, *144*, 143–146.

[5] Watson, P. L.; Tulip, T. H.; Williams, I. Defluorination of Perfluoroolefins by Divalent Lanthanoid Reagents: Activating Carbon-Fluorine Bonds. *Organometallics* **1990**, *9*, 1999–2009.

[6] Fulmer, G. R.; Miller, A. J. M.; Sherden, N. H.; Gottlieb, H. E.; Nudelman, A.; Stoltz, B. M.; Bercaw, J. E.; Goldberg, K. I. NMR Chemical Shifts of Trace Impurities: Common Laboratory Solvents, Organics, and Gases in Deuterated Solvents Relevant to the Organometallic Chemist. *Organometallics* **2010**, *29*, 2176–2179.

[7] Evans, D. F. 400. The Determination of the Paramagnetic Susceptibility of Substances in Solution by Nuclear Magnetic Resonance. *J. Chem. Soc.* **1959**, 2003–2005.

[8] Schubert, E. M. Utilizing the Evans Method with a Superconducting NMR Spectrometer in the Undergraduate Laboratory. *J. Chem. Educ.* **1992**, *69*, 62.

[9] Bain, G. A.; Berry, J. F. Diamagnetic Corrections and Pascal's Constants. *J. Chem. Educ.* **2008**, *85*, 532.

[10] Stoll, S.; Schweiger, A. EasySpin, a Comprehensive Software Package for Spectral Simulation and Analysis in EPR. *J. Magn. Reson.* **2006**, *178*, 42–55.

[11] Spek, A. L. *PLATON* SQUEEZE: A Tool for the Calculation of the Disordered Solvent Contribution to the Calculated Structure Factors. *Acta Crystallogr Sect C* **2015**, *71*, 9–18.

[12] Farrugia, L. J. *WinGX* and *ORTEP for Windows*: An Update. *J. Appl. Crystallogr.* **2012**, *45*, 849–854.
